# Benchmark of cellular deconvolution methods using a multi-assay reference dataset from postmortem human prefrontal cortex

**DOI:** 10.1101/2024.02.09.579665

**Published:** 2024-02-12

**Authors:** Louise A. Huuki-Myers, Kelsey D. Montgomery, Sang Ho Kwon, Sophia Cinquemani, Sean K. Maden, Nicholas J. Eagles, Joel E. Kleinman, Thomas M. Hyde, Stephanie C. Hicks, Kristen R. Maynard, Leonardo Collado-Torres

**Affiliations:** 1. Lieber Institute for Brain Development, Johns Hopkins Medical Campus, Baltimore, MD, 21205, USA; 2. The Solomon H. Snyder Department of Neuroscience, Johns Hopkins School of Medicine, Baltimore, MD, 21205, USA; 3. Department of Biostatistics, Johns Hopkins Bloomberg School of Public Health, Baltimore, MD, 21205, USA; 4. Department of Psychiatry and Behavioral Sciences, Johns Hopkins School of Medicine, Baltimore, MD, 21205, USA; 5. Department of Neurology, Johns Hopkins School of Medicine, Baltimore, MD, 21205, USA; 6. Center for Computational Biology, Johns Hopkins University, Baltimore, MD, 21205, USA; 7. Department of Biomedical Engineering, Johns Hopkins University, Baltimore, MD, 21205, USA; 8. Malone Center for Engineering in Healthcare, Johns Hopkins University, Baltimore, MD, 21218, USA

**Keywords:** Deconvolution, transcriptomics, RNA-seq, snRNA-seq, smFISH, RNAScope, immunofluorescence, benchmark, multi-assay, human brain

## Abstract

**Background:**

Cellular deconvolution of bulk RNA-sequencing (RNA-seq) data using single cell or nuclei RNA-seq (sc/snRNA-seq) reference data is an important strategy for estimating cell type composition in heterogeneous tissues, such as human brain. Several deconvolution methods have been developed and they have been previously benchmarked against simulated data, pseudobulked sc/snRNA-seq data, or cell type proportions derived from immunohistochemistry reference data. A major limitation preventing the improvement of deconvolution algorithms has been the lack of highly integrated datasets with orthogonal measurements of gene expression and estimates of cell type proportions on the same tissue block. The performance of existing deconvolution algorithms has not yet been explored across different RNA extraction methods (e.g. cytosolic, nuclear, or whole cell RNA), different library preparation types (e.g. mRNA enrichment vs. ribosomal RNA depletion), or with matched single cell reference datasets.

**Results:**

A rich multi-assay dataset was generated in postmortem human dorsolateral prefrontal cortex (DLPFC) from 22 tissue blocks. Assays included spatially-resolved transcriptomics, snRNA-seq, bulk RNA-seq across six RNA extraction and RNA-seq library combinations, and orthogonal cell type measurements via RNAScope/Immunofluorescence (RNAScope/IF). The *Mean Ratio* method was developed for selecting cell type marker genes for deconvolution and is implemented in the *DeconvoBuddies* R package. Five extensively benchmarked computational deconvolution algorithms were evaluated in DLPFC across six RNA-seq combinations and predicted cell type proportions were compared to those measured by RNAScope/IF.

**Conclusions:**

We show that *Bisque* and *hspe* are the top performing methods with performance dependent on the RNA-seq library preparation conditions. We provide a multi-assay resource for the development and evaluation of deconvolution algorithms.

## Background

Increasing numbers of bulk RNA-sequencing (RNA-seq) and single cell or nucleus RNA-seq (sc/snRNA-seq) datasets have been generated, sometimes uniformly processed, and publicly shared [1–4]. RNA-seq data historically has been cheaper to generate than sc/snRNA-seq data, leading to a surge in methods that perform cellular deconvolution and estimation of cell type proportions using reference sc/snRNA-seq data [5–11]. Some methods can use these estimated cell type proportions to deconvolve cell type specific gene expression [12–14] to overcome cellular heterogeneity and identify nuanced gene expression signals that would otherwise be masked in bulk RNA-seq data. Some downstream applications include differential expression analysis adjusting for cell type composition confounders [15], cell type specific eQTL discovery [16, 17], and quality control assessment of dissections in heterogeneous tissues.

While each new computational deconvolution method typically compares itself against other leading methods, estimated cell type proportions can be widely variable across methods making it difficult for users to select the appropriate algorithm for a given analysis. Several comprehensive benchmarking efforts have been performed by independent groups [17–20] which evaluated deconvolution methods across a wide range of tissues, simulation scenarios, and normalization methods [17–20]. However, the performance rankings of different deconvolution methods have mostly been inconsistent due to several plausible reasons including 1) the tissue for which they were initially developed did not match the tissue used for evaluation, 2) biases and variability in reference sc/snRNA-seq datasets, 3) variability in the selection of cell type marker genes, 4) the level of cell type heterogeneity in the tissue under study, 5) choice of cell type resolution (fine or broad), 6) factors regarding how the tissue samples were extracted or preserved, 7) differences between the target RNA-seq and reference sc/snRNA-seq data regarding cell compartments profiled and library preparation strategies, and 8) differences in data normalization and processing [17–22].

One main limitation has been the limited availability of “ground truth” or “gold/silver standard” cell type proportions against which deconvolution methods can be benchmarked. In the absence of cell type proportion standards, it has been common to pseudobulk sc/snRNA-seq data or use mixture simulations to generate pseudobulk RNA-seq data, use the same sc/snRNA-seq data as the reference, and compare the deconvolution results against the cell type proportions observed in the sc/snRNA-seq data. However, sc/snRNA-seq library preparation protocols have filtering steps that can introduce biases in the estimated cell type proportions [21], limiting their use as “ground truth” references. Immunohistochemistry in tissue sections can generate orthogonal measurements of cell type proportions and has been used effectively for benchmarking deconvolution algorithms [17, 23]. However, there is a need for more datasets with orthogonal measurements of cell type proportions from the same tissue blocks used for RNA-seq and sc/snRNA-seq [21]. In particular, human brain is a complex heterogeneous tissue which is typically studied from postmortem samples that are fresh frozen to −80°C [21]. Due to this, not all protocols can be applied to study postmortem human brain. For example, flow cytometry cannot serve as a gold standard for deconvolution of human brain tissue because it does not capture all cell types, although it has been successfully used in blood [24].

To add to the complexity, not all bulk RNA-seq studies are equal. Some protocols enrich the cytosolic or nuclear cell fractions [25, 26], while most capture RNAs from the whole cell. In addition to different RNA extraction methodologies, there are several options for RNA-seq library preparation, with two common approaches including poly(A)+ enrichment for mRNA profiling [27] and ribosomal RNA depletion via the Ribo-Zero Gold kit [28] for total RNA profiling. These two library preparations show differences such as polyA having a higher exonic mapping rate, RiboZero/RiboZeroGold having a higher intronic mapping rate, and RiboZero/RiboZeroGold capturing a larger diversity of gene biotypes [29–32]. As RNA-seq and sc/snRNA-seq quantify different RNA populations and sc/snRNA-seq unique molecule identifier (UMI) counts are enriched for zeros [33, 34], these assays present different gene count statistical properties and gene biotype quantification differences. Some deconvolution methods already model statistical differences between sequencing assays [6], while others employ normalization methods. Ultimately, the RNA extraction method and library preparation protocol used for bulk RNA-seq can impact benchmarking results of deconvolution methods [22].

Cellular deconvolution algorithms were originally developed for DNA methylation (DNAm) data, where it is possible to identify CpG sites that are binary markers for a given cell type [35]. In contrast, cell types in sc/snRNA-seq data are defined through cell type marker genes, which can be identified through many different methods [36]. Some methods identify genes with high expression in a target cell type, but these genes may also be expressed in other cell types, making cell type markers identified through sn/scRNA-seq [36–39] noisier than cell type marker CpGs in DNAm data [40, 41]. Improvements in cell type marker gene selection for deconvolution is an active area of development for sn/scRNA-seq data [21].

**Figure 1:**
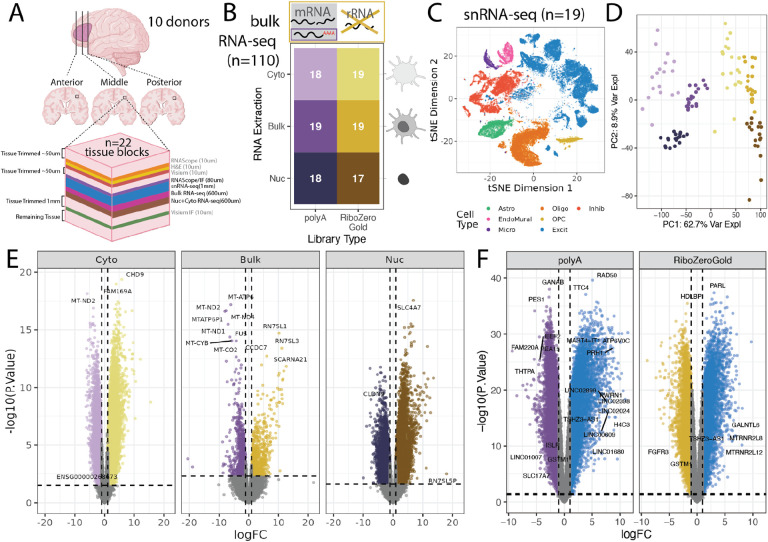
Experimental design overview and exploration of gene detection in different assays. **A.** Human postmortem brain dorsolateral prefrontal cortex (DLPFC) tissue blocks across the anterior to posterior axis from 10 donors were dissected for a total of 19 tissue blocks, these tissue blocks are a subset of the 30 tissue blocks that were used in a previous spatial transcriptomic study [38]. For each block, sequential slides were cut for different assays while maintaining the same white matter vs gray matter orientation. **B.** snRNA-seq data, generated as part of the same spatial transcriptomic study was collected for 19 tissue blocks [38], from which bulk RNA-seq data was also generated across two library preparations (polyA in purple or RiboZeroGold in gold) and three different RNA extractions targeting different cell fractions: cytosolic (Cyto, light color), whole cell (Bulk/Total, intermediate color), or nuclear (Nuc, dark color) in this study. **C.** tSNE plot of the reference snRNA-seq data at the broad cell type resolution. **D.** Scatter plot of bulk RNA-seq principal components (PCs) 1 and 2. PC1 is associated with library type and PC2 with RNA extraction method. Colors are the same as groups in *B*. **E.** Volcano plots for the differential expression analysis between polyA and RiboZeroGold, faceted by RNA extraction method. The colors of the points are the same as *B*. Horizontal dotted line denotes FDR < 0.05 cutoff, vertical dotted lines are logFC = −1 and 1. **F.** Volcano plot for the differential expression analysis between total bulk RNA-seq (point colors same as *E*) and snRNA-seq (blue points). Annotations are the same as *E*.

This study presents a rich multi-assay dataset in postmortem human brain tissue that can be used to rigorously benchmark computational methods for deconvolution of bulk RNA-seq data in heterogeneous tissues. Data from dorsolateral prefrontal cortex (DLPFC) was generated across multiple donors [37, 38], tissue blocks, and modalities. Single molecule fluorescent in situ hybridization (smFISH) and bulk RNA-seq from three RNA extraction protocols and two library types were generated from adjacent tissue sections (Figure 1A), in addition to previously described snRNA-seq and spatial transcriptomics data from the same tissue blocks [38]. In addition to labeling the six most common DLPFC cell types, which served as an orthogonal measurement of cell type proportion, smFISH data also provided experimentally paired measurements of cell size and estimation of total RNA across cell types [42]. This multidimensional dataset from matched tissue blocks is a comprehensive resource for evaluating existing and future deconvolution algorithms in complex tissue with highly organized laminar structure.

In this study, five leading deconvolution algorithms were benchmarked on this multi-assay DLPFC dataset. The methods evaluated were *DWLS* [5], *Bisque* [6], *MuSiC* [7], *BayesPrism* [8], and *hspe* [9] previously known as *dtangle* [43]. These algorithms among the best performers in recent benchmarking studies [17–20, 22] and optimize predictive performance by extension of weighted least squares [5], assay bias correction [6], source bias correction [7], Bayesian methods [8], and high collinearity adjustment [9]. The snRNA-seq served as the reference dataset, using a new method for the selection of cell type marker genes called *Mean Ratio*. *Mean Ratio* identifies cell type marker genes that are expressed in the target cell type with minimal gene expression in non-target cell types. Accuracy of cell type proportion predictions are evaluated against the orthogonal RNAScope/IF cell type proportion measurements from the same tissue block.

## Results

### Transcriptomic Gene Expression Data

To create a multidimensional dataset that can assess the performance of cellular deconvolution methods on a variety of RNA-seq conditions, RNA-seq was performed on 19 tissue blocks across the anterior-posterior axis of the dorsolateral prefrontal cortex (DLPFC) from 10 adult neurotypical control donors with different RNA extraction protocols and RNA-seq library preparations types (Figure 1A, Fig S1B, Table S1). Additionally, combined single molecule fluorescent in situ hybridization (smFISH) and immunofluorescence (IF) data using RNAScope/IF technology was generated on consecutive tissue sections to estimate the proportions of 6 broad cell types as well as the nuclear size and total RNA content for individual cells. Single cell and spatial transcriptomics data were also collected on these same tissue blocks in a previous study by Huuki-Myers et al. [38] (Figure 1A, Fig S1B).

For bulk RNA-seq data, total and fractionated RNA was extracted from 19 DLPFC tissue blocks using adjacent tissue cryosections. Fractionated RNA contained the nuclear (Nuc, n = 38) and cytoplasmic cellular fractions (Cyto, n = 37 as one sample failed). Total, or Bulk, RNA included RNA from whole cells (Bulk/Total, n=38). For each aliquot of RNA, two libraries were prepared using either polyA (n=56) or RiboZeroGold library preparation techniques (n=57) [27, 28]. After sequencing, alignment, and quality control data processing, 110 RNA-seq samples were included in the study (Figure 1B, Fig S2, Fig S3).

For benchmarking deconvolution algorithms, a previously analyzed snRNA-seq dataset taken from the same tissue blocks was leveraged, which serves as a paired snRNA-seq reference dataset [38] (n=19, Figure 1A, Fig S1). This snRNA-seq dataset contains gene expression profiles from 56k nuclei representing 6 broad cell type populations in the DLPFC including, Astrocytes (Astro), Endothelial/Mural cells (EndoMural), Microglia (Micro), Oligodendrocytes (Oligo), Oligodendrocyte Precursor Cells (OPC), Excitatory neurons (Excit), and Inhibitory (Inhib) neurons (Figure 1C).

Principal Component Analysis (PCA) of Bulk/Total and fractionated RNA-seq samples showed that there were large differences in gene quantification between the library preparation types and RNA extraction methods. PC1, which explained the largest percent (62.7%) of variation (62.7%) showed a clear split between polyA and RiboZeroGold library types. PC2, the next largest percent of variation (8.9%), showed separation between Bulk/Total, Cyto, and Nuc RNA extractions (Figure 1D).

Differential gene expression analysis further revealed large differences in gene quantification when RNA libraries from the same tissue block were prepared with polyA or RiboZeroGold RNA library types, and also when prepared with different cell fractions (Cyto, Nuc, or Bulk/Total) (Figure 1E, Fig S4). Between library types in the Bulk/Total RNA extraction, out of 21,745 genes, 996 (4.58%) of genes were over-quantified in RiboZeroGold, and 1,005 (4.62%) in polyA (FDR<0.05). The other cellular fractions showed differences in quantification between library types; Cyto had 7,109 (32.7%) in RiboZeroGold, and 4,949 (22.8%) in polyA; and Nuc had 5,821 (26.8%) in RiboZeroGold, and 4,084 (18.8%) in polyA (Figure 1E). Between RNA extractions in the same library preparation there were also notable differences in gene quantification. For RiboZeroGold the largest number of differences was between the Cyto and Nuc extractions (1,887 [8.68%], and 2,775 [12.8%] respectively), for polyA the largest differences was between the Bulk/Total and Cyto extractions (3,269 [15.0%], and 2,639 [12.1%] respectively, Fig S4).

There were also large differences in gene expression quantification between the snRNA-seq data (pseudobulked by tissue block) compared to the corresponding Bulk/Total extraction RNA-seq data (Figure 1F). Out of 17,660 common genes, in RiboZeroGold 4,423 (25.0%) were over-quantified in Bulk/Total RNA-seq, and 4,335 (24.5%) in snRNA-seq, in polyA 5,509 (31.2%) were over-quantified in Bulk/Total RNA-seq, and 5,749 (32.6%) in snRNA-seq (FDR<0.05). This showed that RNA library preparation and assay type has a significant impact on gene expression datasets.

### RNAScope/IF Imaging Data

**Figure 2:**
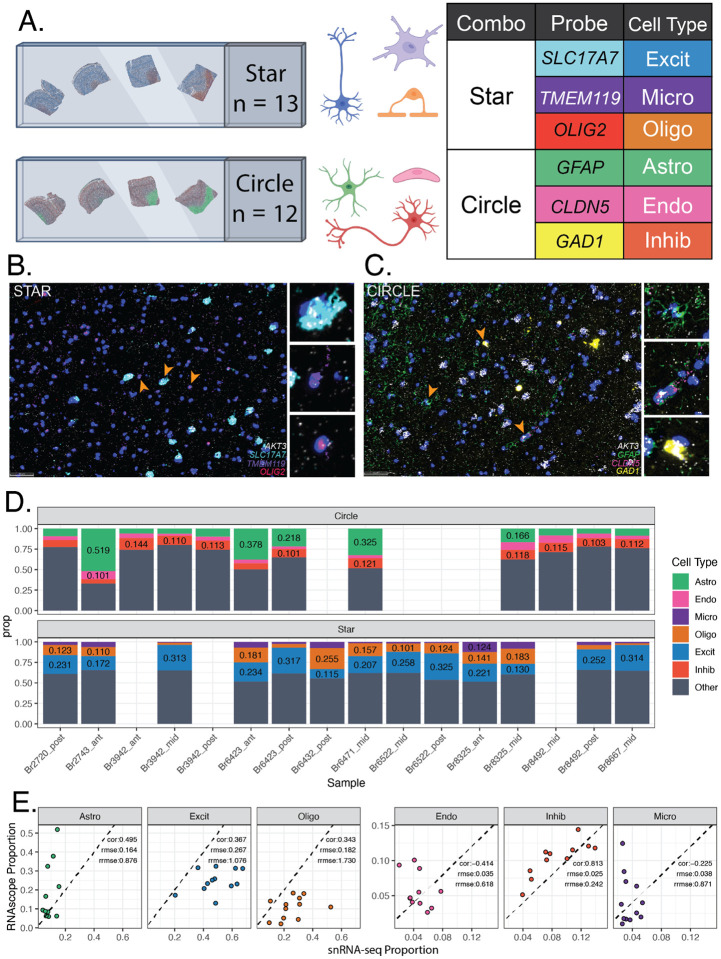
Estimated cell type proportions in tissue sections labeled with RNAScope/Immunofluorescence. **A.** Schematic of experimental design for combined single molecule fluorescent in situ hybridization (smFISH) with immunolabeling using RNAScope/immunofluorescence (IF). The “Star” combination of probes/antibodies marked Excitatory Neurons [Excit], Microglia [Micro], and Oligodendrocyte [Oligo] cell types in 13 tissue blocks. The “Circle” combination marked Astrocytes [Astro], Endothelial cells [Endo], and Inhibitory Neuron [Inhib] cell types in 12 tissue blocks. The total RNA expression gene (TREG) [42], *AKT3*, was also included in each combination to estimate RNA abundance. **B.** Representative fluorescent image of a single field for the “Star” combination showing expression of *SLC17A7*, TMEM119, OLIG2*, AKT3* and the nuclear marker DAPI. **C.** Representative fluorescent image of a single field for the “Circle” combination showing expression of GFAP, CLDN5, and *GAD1*. *SLC17A7*, *GAD1*, and *AKT3* are labeled with RNA probes while TMEM119, OLIG2, GFAP, CLDN5 with antibodies. **D.** Barplots of estimated cell type proportions from RNAScope/IF data for “Circle” and “Star” experiments. **E.** Scatter plots of cell type proportions estimated from snRNA-seq data (x-axis) vs. RNAScope/IF, annotated with the Pearson correlation (cor), root mean squared error (rmse), and relative rmse (rrmse) against the mean RNAScope/IF proportions.

To estimate cell type proportions of six major cell types in the DLPFC using an orthogonal assay, multiplex single molecule fluorescent in situ hybridization (smFISH) combined with immunofluorescence (IF) was employed. Across 21 tissue blocks, two probe combinations were designed including three cell type markers, a total RNA expression marker gene *AKT3* [42], and a nuclear marker DAPI. One RNAScope/IF marker combination “Star” included *SLC17A7* (marking Excit), *TMEM119* (Micro), *OLIG2* (Oligo), and *AKT3*. The second combination “Circle” included *GFAP* (Astro), *CLDN5* (Endo), and *GAD1* (Inhib), and *AKT3* (Figure 2A, Fig S1B, Table S2). Following staining and imaging, cells were segmented and classified with HALO (Indica Labs). Quality control analysis showed “Star” tissue sections (n=12) had a median of 37,553 cells (range 28,093:53,709), whereas “Circle” (n=13) tissue sections had a median of 44,835 cells (range 32,425:57,674, Methods). Cells were phenotyped based on expression of cell type marker probes/antibodies using HALO (Indica Labs, Figure 2B–C, Methods). In a given tissue section, proportions were calculated for each labeled cell type. Across tissue sections, the median proportions for each cell type were Astro=0.09, Endo=0.04, Inhib=0.11, Excit=0.23, Micro=0.03, and Oligo=0.12 (Figure 2D, Fig S5, Table S3).

The cell type proportions measured by RNAScope/IF varied from the proportions measured by snRNA-seq for the same sample (Star n=12, Circle n=11). The two proportion estimates were compared with Pearson correlation (cor) and relative root mean squared error (rrmse) to the mean of the RNAScope/IF-derived proportions. Inhib had the closest proportion values to the snRNA-seq with the highest correlation (0.813) and lowest rrmse (0.242). Other cell types were inconsistent between assays with Endo showing the lowest cor (-0.414) and Oligo showing the highest rrmse (1.73) (Figure 2E).

### Selection of Deconvolution Marker Genes: *Mean Ratio* Method

**Figure 3:**
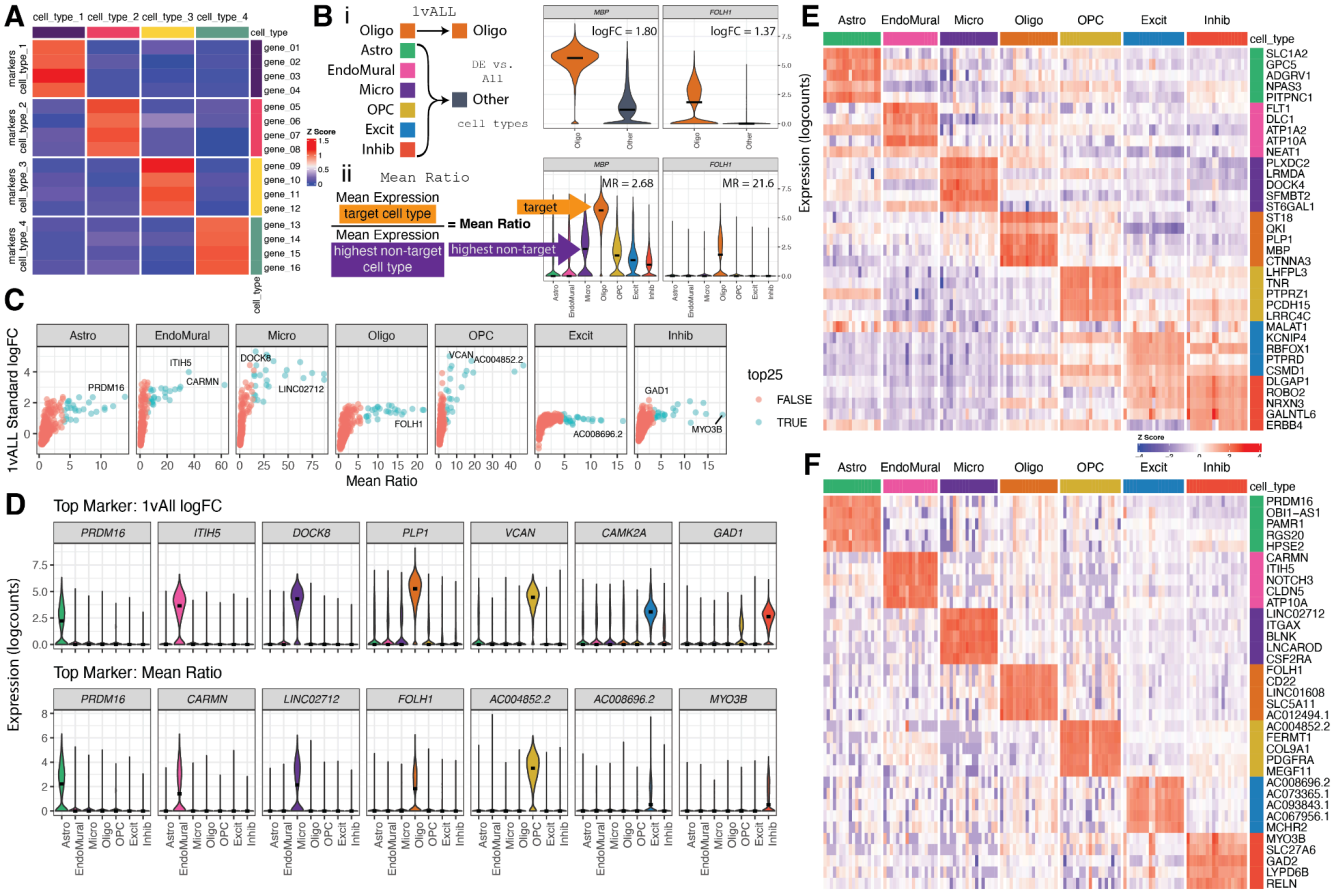
Establishing cell type marker genes in snRNA-seq reference data. **A.** Schematic of an ideal deconvolution cell type marker gene heatmap, where the marker genes for a target cell type (rows) only have high expression in the target cell type (columns), and low expression for all other cell types. **B.** Illustration of marker gene selection strategies for an example target cell type, Oligo. **i.** *1vALL* combines the non-target cell types into one group and identifies differentially expressed genes between the two groups. **ii.** *Mean Ratio* maintains all the cell type groups, finds the ratio between the mean expression of the target cell type, and the highest mean expression from a non-target cell type. **C.** Scatter plots of the *Mean Ratio* value vs. *1vALL* standard log fold change, for all genes by cell type. The top 25 ranked by *Mean Ratio* are indicated by point color, and the top gene from both methods is annotated with the gene symbol. **D.** Violin plots of the gene expression over cell types for the top gene from *1vALL* (top row) and *Mean Ratio* (bottom row) methods. **E.** Heat map of the top 5 marker genes from *1vALL* logFC. Rows are genes, columns are pseudobulked samples by cell type and tissue block. Color is the Z-score of logcount expression scaled by gene. **F.** Heat map (similar to *E*) of the top 5 *Mean Ratio* marker genes.

To eliminate noise in deconvolution analyses, cell type marker genes should have cell type-specific expression characterized by high expression in the target cell type and low expression in other cell types (Figure 3A). There are several currently available methods to identify cell type marker genes, such as “1 vs. All” differential expression analysis (*1vALL*) [36, 39]. However, cell type marker genes identified by this approach may also be expressed in other cell types. Therefore, the *Mean Ratio* method was developed to identify marker genes with cell type-specific expression that are more ideal for deconvolution analyses. *Mean Ratio* is defined by calculating the ratio of the mean expression of a gene in a target cell type over the highest mean expression of the non-target cell types. Genes with the highest *Mean Ratio* above 1 are the best maker genes for the target cell type (Figure 3B). Genes with the highest *Mean Ratios* often had the highest log fold changes compared to the *1vALL* method, whereas the opposite was not true as *1vALL* was more permissive of expression in non-target cell types (Figure 3C). In the DLPFC snRNA-seq data [38], the *Mean Ratio* method identified cell type marker genes with less noisy signal among non-target cell types compared to *1vALL*. In contrast, the top marker genes selected by *1vALL* showed some expression in non-target cell types (i.e., *PLP1*, an Oligo marker gene, had some expression in several non-Oligo cell types), (Figure 3D). Heatmaps of the top sets of marker genes from each method showed more cell type specific expression for marker genes selected by *Mean Ratio* (Figure 3E–F). For subsequent deconvolution analyses, a final set of 151 marker genes were selected; genes in this set were both in the top 25 genes ranked by the *Mean Ratio* value for each of the 7 cell types and also present in the filtered RNA-seq data (Table S4, Fig S6).

### Benchmark of Selected Deconvolution Methods

To test the performance of different approaches to computational deconvolution of RNA-seq data (Table 1), five deconvolution methods (*DWLS*, *Bisque*, *MuSiC*, *BayesPrism*, and *hspe*) were run on this dataset of 110 bulk/fractionated RNA-seq samples [5–9]. The reference snRNA-seq dataset was subset to the top 25 *Mean Ratio* cell type marker genes for deconvolution. Cell type proportion predictions for each sample varied for each method, and over RNA extraction and RNA-seq library type (Fig S7).

**Figure 4:**
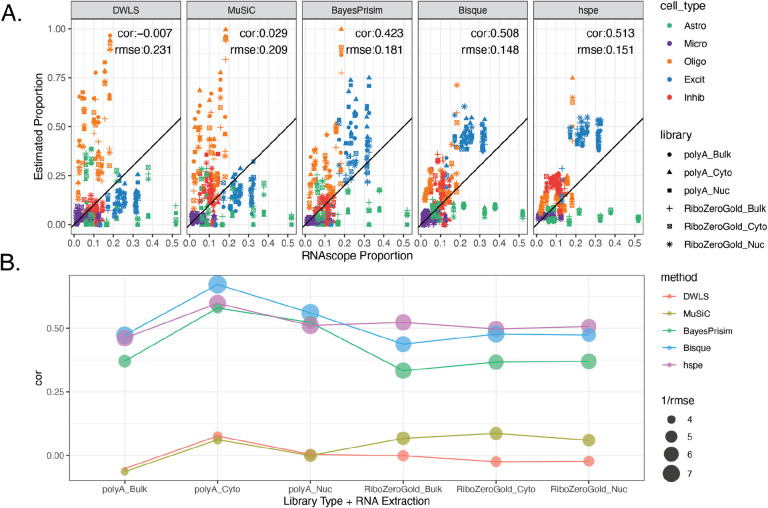
Deconvolution methods performance with *Mean Ratio* marker genes. **A.** Scatter plot of cell type proportions estimated by RNAScope/IF (x-axis) vs. the predicted cell type proportions by the deconvolution methods. Points are colored by the cell type and shaped by the combination of bulk RNA-seq RNA extraction method and library type. The annotation lists the overall Pearson’s correlation (cor) and root mean squared error (rmse). **B.** Correlation (color) between the predicted proportions by deconvolution methods and the estimated RNAScope/IF proportions across RNA extraction method and library type combinations. The 1/rmse values are shown by dot size, where rmse values of 0.25, 0.2, 0.167, and 0.143 respectively correspond to 1/rmse of 4, 5, 6, and 7.

To evaluate the accuracy of these deconvolution methods, predicted cell type proportions were compared to estimated cell type proportions from RNAScope/IF data and evaluated by Pearson’s correlation (cor) and root mean squared error (rmse) (Figure 2D). Across all six RNA-seq variations for each tissue block, *hspe* had the highest correlation with RNAScope/IF proportions (cor=0.513, rmse=0.151), followed closely by *Bisque* (cor=0.508, rmse=0.148) (Figure 4A). Other methods ranked: third *BayesPrism* (cor=0.423, rmse=0.181), fourth *MuSiC* (cor=0.029, rmse=0.209), and fifth *DWLS* (cor=-0.007, rmse=0.231) (Figure 4A).

Over the six RNA extraction and library type combinations, performance of the five deconvolution algorithms varied. While *Bisque* had the highest correlation across polyA library types, *hspe* was the top performer for RiboZeroGold, although differences in both cases was marginal (Figure 4B, Fig S8). *BayesPrism* performed similarly well to *Bisque* and *hspe* in polyA_nuc and polyA_cyto (Figure 4B, Fig S8). *MuSiC* and *DWLS* were the poorest performers across the data types (Figure 4B, Fig S8). Cell type proportion predictions between *Bisque* and *hspe* were similar (cor = 0.939). Similarly, correlation was boosted between *MuSiC* and *DWLS* (cor = 0.890), likely related to their adjustment on multi-reference bias sources*. Bisque* had the highest correlation with the snRNA-seq proportions (cor = 0.738) (Fig S9). Overall *Bisque* and *hspe* produced similar cell type proportion predictions for this DLPFC dataset, and they had the highest accuracy measured by correlation against cell type proportions derived from the RNAScope/IF data.

## Discussion

This study presents a comprehensive reference dataset across bulk RNA-seq, snRNA-seq, and RNAScope/IF data from the DLPFC from postmortem human brain samples that can be used for multi-assay data integration (Figure 1A). In particular, this dataset can be used to benchmark the performance of RNA-seq deconvolution algorithms based on reference snRNA-seq data and address challenges presented when computationally deconvolving heterogeneous tissue [21]. A unique aspect of this study is that RNAScope/IF is used to label the six broad cell types of the DLPFC providing estimates of cell type proportions that circumvent some of the pitfalls of measuring cell type proportions with snRNA-seq, which are driven by flow sorting and other quality control steps [21]. Given the spatially-resolved transcriptomics data from the same tissue blocks and from adjacent tissue slices [38], this rich dataset presents many opportunities for multi-assay integration studies.

Most RNA-seq deconvolution methods and benchmark studies have overlooked the fact that not all bulk RNA-seq datasets are generated using the same methodology. While most publicly available RNA-seq datasets have been generated with Illumina sequencers, different RNA extraction kits and RNA-seq library types can be utilized. Comparison of data from different RNA extraction methods and library types showed large differences in gene quantification. For a deconvolution algorithm to be applicable across diverse datasets, it should perform well across different RNA-seq data types. In this regard, this multi-assay dataset is a useful benchmarking tool. Of the deconvolution algorithms that were evaluated, *hspe* [9] and *Bisque* [6] performed similarly and were the top two performers (Figure 4). Across bulk RNA-seq library types, *Bisque* and *hspe* were marginally the best for bulk RNA-seq data generated with polyA and RiboZeroGold library kits, respectively (Figure 4B). Identification of *Bisque* and *hspe* (an update on *dtangle* [43]) as the most accurate deconvolution algorithms was consistent with other independent benchmark findings using human brain data [17].

As has been noted previously [17], immunohistochemistry and sc/snRNA-seq proportions do not always match. Differences in cell type proportions were observed between RNAScope/IF and snRNA-seq assays across all cell types. Astrocytes were significantly undercounted while oligodendrocytes were overcounted in snRNA-seq compared to RNAScope/IF (Figure 2E). As some bulk RNA-seq deconvolution methods have a tendency to infer cell type proportions similar to those observed in the reference snRNA-seq data [6], this discrepancy in cell type proportions between assays likely drove some inaccuracies observed when benchmarking RNA-seq deconvolution methods against RNAScope/IF-derived cell type proportions. Most evaluated methods underestimated the proportion of astrocytes and frequently overestimated the proportion of oligodendrocytes (Figure 4A), matching the discrepancies observed between the snRNA-seq and RNAScope/IF proportions. This is important to keep in mind when choosing a deconvolution computational algorithm as global performance across all cell types could be misaligned with performance for an individual cell type of interest.

This study design also highlighted some challenges with using RNAScope/IF data as reference for cell type proportions. Even with the utmost care while generating RNAScope/IF images, artifacts can occur that affect a large part of the tissue section. These imaging artifacts can skew the cell type proportions on spatially arranged tissue and cell types such as in the DLPFC [37, 38] (60% passed QC checks, Fig S1B). Imaging experiments performed were also limited to select broad cell types given the limitation on the number of probes that can be used in a single RNAScope/IF image and the scope of this study. Future studies could investigate rarer cell types and finer cell type resolutions. Despite limitations noted for RNAScope/IF-based cell type labeling, sc/snRNA-seq assays also have their own challenges such as dissociation bias and a partial capture of all cells affecting the accuracy of cell type proportion estimates [21, 44, 45]. Ignoring limitations of sc/snRNA-seq protocols and using pseudobulked versions of the data to benchmark deconvolution algorithms can potentially lead to misleading conclusions on the performance of deconvolution computational algorithms. Thus, generating orthogonal measurements across technologies can be informative, despite the associated challenges.

An often overlooked challenge for applying computational deconvolution algorithms is the selection of cell type marker genes. Unlike cell type deconvolution using DNA methylation data, where one can find CpGs that are either fully methylated or unmethylated in a given cell type [35, 46, 47], finding binary (on/off) cell type marker genes in RNA-seq data is challenging. There are many statistical methods for finding sc/snRNA-seq cell type marker genes that have different properties [36], with findMarkers() from *scran* [39] implementing many options which are commonly used in Bioconductor-based analysis workflows [48]. One option is to perform a *t*-Student test comparing a given cell type of interest and all other cell types combined into a single category (*1vAll*), which can help identify genes that have significantly higher expression in the target cell type. However, the *1vAll* strategy does not penalize genes that have high expression in outlier cells among the non-target cell type. The *Mean Ratio* method developed here was designed to find cell type marker genes that are not only more highly expressed in the target cell type, but also have the cleanest signal compared to the second highest cell type (Figure 3). *Mean Ratio* cell type marker genes can help provide more specific inputs to cell type deconvolution algorithms. However, using a small set of marker genes per cell type is prone to overfitting given the variability in gene expression measurements for the same gene across bulk RNA-seq and sc/snRNA-seq assays. Future work can explore how algorithms for cell type marker gene selection affect deconvolution estimation accuracy as well as evaluate the impact of different marker gene set sizes for a given marker gene selection method.

As sc/snRNA-seq assays have matured, the number and scale of publicly available datasets has increased in recent years [1, 2]. Specifically for human brain, large efforts such as the BRIAN Initiative Cell Census Network (BICCN) and the PsychENCODE Consortium (PEC) have expanded the understanding of cell clusters, states, and types in neurotypical donors as well as those affected by different psychiatric disorders [49–51]. In some scenarios, it may be more important to have access to a large and diverse sc/snRNA-seq reference dataset for deconvolution. For instance, the leave-one-out cross-validation performance across 8 donors in Jew et al. revealed large performance gains for *Bisque* when increasing the reference size from 2 to 4 donors [6]. Some computational deconvolution methods such as *SPLITR* have already proposed how to incorporate variability across donors in the reference sc/snRNA-seq data, such as age, sex, and disease status [10]. Using this new dataset, it will be useful to assess the impact on deconvolution accuracy from 1) improvements in the reference datasets, 2) integrating multiple input references or 3) adjusting for reference donor covariates.

RNAScope/IF can be used to estimate cell size and total RNA expression [42]. This is important as cell size has different associations with total RNA expression across cell types [42], which can potentially impact how cell size and total RNA scaling factors are implemented in deconvolution methods. For example, due to differences in cell sizes, it is likely that current cellular deconvolution algorithms recover the fraction of RNA in different cells rather than estimating cell composition directly [21, 47]. Adjusting cell size differences as well as the RNA content of different cell types could potentially improve the accuracy of RNA-seq deconvolution methods [47].

Benchmarking in general is a challenging endeavor as new technological improvements and software implementations can impact accuracy in different ways [21]. Benchmarking studies face the complication that a “ground truth” is most commonly not available or might be biased. This is why many sc/snRNA-seq studies initially pseudobulked sc/snRNA-seq data, deconvolved the pseudobulk data with the same reference data, and evaluated the results against the cell type proportions on the same sc/snRNA-seq reference data. Variability across tissues, or brain regions as evaluated in Figure 2 by Park et al. [10], can provide a qualitative guide on the accuracy of the results when cross-referenced with experts in the expected cell type proportions on a given tissue. Removing a cell type as well as using a thousand pseudobulk mixtures of reference datasets [18] are great complimentary evaluation strategies of deconvolution methods to the ones presented in this study that can be used to further evaluate the effects of different normalization strategies [18, 19]. The dataset from this study enriches the options available for benchmarking computational deconvolution algorithms by providing multi-assay data from adjacent tissue slices on a heterogenous tissue and by investigating the effects from RNA-seq extraction and library type preparations. The RNAScope/IF-derived cell size and RNA total expression data provided will enable comparisons of the performance of methods that can adjust for or incorporate these variables given that cell size and total RNA present heterogenous relationships across cell types [42]. Deconvolution algorithms already can go beyond estimating cell type proportions and estimate cell type specific gene expression values [12–14], which can be very useful for cell type specific eQTL analyses and other applications [17]. However, accurate cell fractions estimates are still needed as they are the input for these methods [17], and inaccurate estimated cell type proportions will likely lead to inaccurate downstream results.

## Conclusion

Estimation of cell type proportions in bulk RNA-seq data using snRNA-seq reference-based deconvolution methods presents many challenges. Here we provide a resource for addressing these challenges by generating a multi-assay dataset from adjacent tissue sections across a set of tissue blocks. Different bulk RNA-seq library types and RNA extraction kits were surveyed. Broad cell type proportions using RNAScope/IF were generated, and other data types such as cell sizes, total RNA, and spatially-resolved transcriptomics data are available as well. This data-rich study can serve as the basis for benchmarking the performance of deconvolution algorithms in heterogeneous tissues, such as the human brain. Of the deconvolution algorithms that were evaluated, *hspe* and *Bisque* were the top performers across different RNA extraction kits and RNA-seq library preparation types. These results were computed using an improved set of cell type marker genes identified by the *Mean Ratio* method that maximizes the difference between the target cell type and the second transcriptionally closest cell population. The highly integrated orthogonal datasets generated here are an important building block for further benchmarking and developing computational deconvolution methods for RNA-seq data.

## Methods

### Cryosectioning and Tissue Sample Collection of Orthogonal Datasets

Assays were completed using 22 individual blocks of postmortem human DLPFC tissue collected across anterior (Ant), middle (Mid), and posterior (Post) positions (Fig S1). These were a subset of the same tissue blocks used for Visium (10x Genomics) and 3’ gene expression snRNA-seq assays described in Huuki-Myers et al. [38]. Tissue sections for the majority of assays were cryosectioned on the same day for each run (i.e. a round of 3–4 tissue blocks balanced across anterior-posterior DLPFC axis) to minimize tissue loss that occurs when obtaining a flat cutting face on the tissue block (Figure 1A, Fig S1A). In a Leica 3050 cryostat, blocks were allowed to equilibrate for 30 minutes prior to mounting with optimal cutting temperature (OCT) medium. Excess OCT was scored from each side of the block using a chilled razor blade to minimize interference of OCT in RNA extraction. Each block was trimmed to achieve a flat cutting face and then several 10um serial sections were collected for RNAScope/immunofluorescence (IF) assays across the 3–4 tissue blocks in that round. In particular, eight slides containing 4 tissue sections each were collected per round, with one tissue section from each block on a given slide. Following collection of serial sections for RNAScope/IF, the cutting thickness was adjusted to 100um, and ten serial sections (~1mm of tissue) were collected for single nucleus RNA sequencing (snRNA-seq). Processing and analysis of snRNA-seq data was reported in Huuki-Myers et al. [38]. Immediately following tissue collection for snRNA-seq, six 100um serial sections (~600um tissue) were collected for bulk RNA extraction. Six additional 100um serial sections were collected for fractionated (nuclear/cytosolic) RNA extraction (Fig S1B). Collected tissue was stored in Eppendorf tubes at −80°C until use.

### Bulk/Total RNA Extraction & Sequencing

Total RNA was extracted from tissue aliquots (2 extractions per block for 19 tissue blocks, n=38, Fig S1B) using the Qiagen RNeasy mini kit (RNeasy Mini Kit, Cat No. 74104, Qiagen, Hilden, Germany) . A modified version of Qiagen’s “Purification of Total RNA from Animal Tissues” protocol from the 2020 version of the RNeasy Mini Handbook was used. Briefly, tissue cryosections were homogenized via wide bore pipette in 0.7 mL of Trizol. Next, 0.14 mL of chloroform was added, and the aqueous phase of the gradient was removed and transferred to a new tube. An equal volume of 70% ethanol was added, and then the mixture was put onto an RNeasy mini column. At this point, RNA was extracted according to the manufacturer’s instructions with DNAse digestion treatment. RNA quantity was measured using a Qubit 4 fluorometer (Qubit 4 fluorometer; Qubit dnDNA HS Assay Kit, Cat No. Q32854 Invitrogen, Eugene, OR, United States). RNA quality was assessed using an Agilent RNA Nano kit on a BioAnalyzer instrument (RNA 6000 Nano Kit, Agilent, Santa Clara, CA, United States). Libraries were subsequently prepared and sequenced at Psomagen. For each sample, 100–500ng of RNA from the same tube was used to prepare a “RiboZeroGold” library with the TruSeq Stranded Total RNA with Ribo-Zero Gold Library Prep kit (Illumina) and “PolyA” library with TruSeq Stranded mRNA Library Prep Kit (Illumina) according to manufacturer’s instructions. Libraries were sequenced on an Illumina Novaseq 6000 targeting 80 million reads per sample. ERCC spike in sequences were included in all samples except the initial pilot round (n = 24).

### Cytoplasmic/Nuclear RNA Extraction

Fractionated RNA extraction was performed on tissue aliquots using the Cytoplasmic and Nuclear RNA Purification kit (Norgen Biotek, Cat. No. 21000, ON, Canada) (Cyto n=38, Nuc n=37, Fig S1B) according to the “Animal Tissues” protocol in the manufacturer’s manual (PI21000–19, section B). Briefly, reagent J was added to the tissue, which was homogenized via a wide bore pipette. Lysate was spun resulting in a supernatant and a pellet. The supernatant was removed and used for the cytoplasmic fraction, and the pellet was retained for the nuclear fraction. For cytoplasmic RNA purification, buffer SK and 100% ethanol were added to the supernatant, which was then transferred to a spin column, and centrifuged. Flow through was discarded and on column DNA removal was completed (RNase-Free DNase I Kit, Cat No. 25710, Norgen Biotek, ON, Canada) For nuclear RNA purification, the pellet was resuspended in buffer SK and 100% ethanol was added. Lysate was then passed through a 25 gauge needle 5 times and added to a different spin column. Following centrifugation, the flow through was discarded. For both fractions, the spin columns were washed twice with Wash Solution A before drying the membranes. Cytoplasmic and nuclear RNA were eluted from each column in Elution Buffer E. Both fractions of RNA were stored at −80 degrees Celsius. RNA quality and quantity was measured as described above for Bulk/Total RNA extraction. RiboZeroGold and PolyA libraries were subsequently prepared and sequenced at Psomagen as described above. In total 113 RNA-seq samples were generated (19 * 2 * 3 = 114 minus one) as a sample failed during library preparation due to an insufficient amount of starting material (Fig S1B).

### Bulk RNA-seq Data Processing & Quality Control

FASTQ files were aligned to Gencode v40 using *SPEAQeasy* [52]. The settings were: --sample “paired” ,--reference “hg38”, --strand “reverse”, --strand_mode ‘accept’ --ercc. This resulted in 4.6 to 155.9 million reads mapped per RNA-seq sample (median 84.2, mean 89.9) with an overall mapping rate (overallMapRate) of 0.2148 to 0.9910 per sample (median 0.9737, mean 0.9586). The dataset contained 61,544 genes.

Sample quality was evaluated to exclude samples with low concordMapRate, numMapped, numReads, overallMapRate, totalAssignedGene, and totalMapped or high mitoRate. See https://research.libd.org/SPEAQeasy/outputs.html#quality-metrics for the definition of these QC metrics. Samples were classified as “drop” (n=2), “warn”(n=10), or “pass” (n=101) based on their relationship to cutoffs for each metric determined by 3 median absolute deviations (MADs) from the median value for ether polyA or RiboZeroGold library type samples as calculated using isOutlier() from *scran* [39]. All *SPEAQeasy* metrics and QC classifications are available (Table S1).

Two samples were flagged to drop: 2107UNHS-0293_Br2720_Mid_Nuc (low concordMapRate, numMapped, overallMapRate, totalAssignedGene), and AN00000904_Br2743_Ant_Cyto (low numMapped, numReads, and totalMapped) and excluded from downstream analysis (Fig S1B, Fig S2).

### Bulk RNA-seq Dimension Reduction & Expression Filtering

Principal component analysis (PCA) was performed on the n=111 samples that passed sequencing quality control checks. PCs were computed using prcomp()on log2(RPKM+1) gene expression values, filtered for mean RPKM > 0.1. One sample AN00000906_Br8492_Mid_Nuc, previously classified as “warn” based on QC metrics, was identified as an outlier for PC2 and PC5 (Fig S3). This sample was excluded from downstream analysis, bringing the total bulk RNA-seq samples to n=110 (Figure 1B, Fig S1B). Genes with low expression were excluded from the dataset with expression_cutoff(log2(RPKM+1))from *jaffelab* v0.99.32 (https://github.com/LieberInstitute/jaffelab); 21,745 genes remained after filtering. The PCA analysis was repeated on the filtered data set (Figure 1D).

### Single Nucleus RNA-seq Data

Single nucleus RNA-seq data collection and analysis from these same tissue blocks (n=19) is described in Huuki-Myers et al. [38] (Figure 1B, Fig S1B). Only “broad” cell type resolution was considered in this study (Figure 1C).

### Bulk RNA-seq Differential Expression

Differential Gene Expression (DGE) was performed between the two library types (PolyA vs. RiboZeroGold) for the three RNA extraction protocols (Cyto, Bulk/Total, and Nuc) (Figure 1E), as well as between the RNA extractions (ex. Bulk/Total vs. Nuc) for both library types (Fig S4). *DREAM* was to leverage the increased power and reduction of false positives in DGE in experiments with multiple samples per donor, as in this experiment’s design [53]. DGE was performed with calcNormFactors() from *edgeR* v3.42.4 [54], voomWithDreamWeights() and dream() from *variancePartition* v1.30.2 [53], and eBayes() and topTable() from *limma* v3.56.2 [55]. To test between library types, samples were separated by RNA extraction, the model used was ~library_type + (1|BrNum) + mitoRate + rRNA_rate +totalAssignedGene, where BrNum is the donor identifier. To test between RNA extractions samples were separated by RNA library type and compared in pairwise fashion between the three extractions (Bulk/Total vs. Cyto, Bulk/Total vs. Nuc, and Cyto vs. Nuc), the model used was ~library_prep + (1|BrNum) + mitoRate + rRNA_rate + totalAssignedGene.

### Bulk/Total vs. snRNA-seq Differential Expression

To explore gene expression quantification differences between snRNA-seq and Bulk/Total RNA-seq data, DGE with *DREAM* was performed (same methodology above) between the Bulk/Total RNA-seq samples (polyA and RiboZeroGold, Bulk/Total RNA extraction) [53], and the corresponding pseudobulked snRNA-seq samples (Figure 1F). The snRNA-seq samples were pseudobulked with registration_pseudobulk(var_registration= “BrNum”, var_sample_id = “Sample”) from *spatialLIBD* v1.12.0 [56]. The model used was ~data_type + (1|BrNum)where data_type was bulk or snRNA-seq. QC metrics could not be accurately computed for the pseudobulked snRNA-seq samples and were excluded from this DGE analysis.

### RNAScope/Immunofluorescence data generation and HALO analysis

To quantify six broad cell types across tissue sections (n=16 sections), multiplex single molecule fluorescent in situ hybridization (smFISH) with RNAScope technology (Advanced Cell Diagnostics) was performed in combination with immunofluorescence (IF) using the RNAScope Fluorescent Multiplex Kit v.2, 4-plex Ancillary Kit, and RNA-Protein co-detection ancillary kit (Advanced Cell Diagnostics ACD, Cat No. 323100, 323120, and 323180, Newark, CA. United States) as previously described [57] (Fig S1). Briefly, the combined RNAScope/IF protocol involved fixing tissue sections in chilled 10% neutral buffered formalin (NBF), dehydrating in a series of graded alcohols, treating with hydrogen peroxide, and incubating overnight with primary antibodies for GFAP (Thermofisher, Cat No.13–0300, Waltham, MA. United States), Claudin 5 (Thermofisher, Cat No. 35–2500, Waltham, MA. United States), TMEM119 (Sigma Aldrich, Cat No. HPA051870–100UL, St. Louis, MO. United States), and OLIG2 (R&D systems, Cat No. AF2418-SP, Minneapolis, MN. United States) (Table S2). Sections were fixed again in 10% NBF, permeabilized with protease IV, and hybridized with probes for *AKT3*, *GAD1*, and *SLC17A7* (Table S2; ACD, Cat No. 434211, 404031-C2, and 415611-C3, Newark, CA. United States). According to manufacturer’s instructions, probes were amplified using AMPs 1–3 and labeled with Opal dye 520, 570, 620, or 690 (Table S2, Akoya Biosciences, Cat No. FP1487001KT, FP1488001KT, FP1495001KT, and FP1497001KT, Marlborough, MA. United States, respectively). Antibodies were labeled with appropriate host species secondary antibodies (Table S2): donkey anti-mouse IgG conjugated to Alexa 488 (Thermofisher, Cat No. A-21202), donkey anti-rabbit IgG conjugated to Alexa 555 (Thermofisher, Cat No. A-31572, Waltham, MA. United States), donkey anti-rat IgG conjugated to Alexa 594 (Thermofisher, Cat No. A-21209, Waltham, MA. United States), or donkey anti-goat IgG conjugated to Alexa 647 (Thermofisher, Cat No. A-21447, Waltham, MA. United States). Finally, sections were stained with DAPI and mounted with FluromountG (Southern Biotechnology, Cat No. 0100–01, Birmingham, AL. United States). Slides were imaged using a Polaris slide scanner (Akoya Biosciences, Marlborough, MA. United States) (Figure 2B). The final QPTIFF files were further pre-processed to generate spectrally unmixed slide image dataset, using Phenochart (Akoya Biosciences, Marlborough, MA. United States), inForm (Akoya Biosciences, Marlborough, MA. United States), and HALO^®^ image analysis platform (Indica labs, Albuquerque, NM. United States), respectively.

As previously described [57], images were analyzed with HALO software (Indica Labs) using the FISH-IF module to quantify 1) the number of cells for each broad cell type in a tissue section, 2) cell size measured by nuclear area, and 3) number of *AKT3* puncta per nucleus, with reference to the manufacturer’s guidelines: HALO 3.3 FISH-IF Step-by-Step guide (Indica labs, Version 2.1.4 July 2021) and Digital Quantitative RNAScope Image Analysis Guide (Indica labs). Segmentation was optimized for accurate identification of punctate *AKT3* RNAScope/IF signals as well as each cellular object for excitatory neurons (*SLC17A7*), inhibitory neurons (*GAD1*), astrocytes (GFAP), microglia (*TMEM119*), oligodendrocytes (OLIG2), and endothelial/mural cells (Claudin 5: CLDN5), using user-defined size and intensity thresholds. Two RNAScope/IF probe combinations were used: Star (*SLC17A7*, TMEM119, OLIG2, *AKT3*, DAPI) and Circle (GFAP, CLDN5, *GAD1*, *AKT3*, DAPI, Figure 2A–C, Table S2). To estimate RNA abundance in each cell type, *AKT3* was included in each combination as a representative total RNA expression gene (TREG) [42]. A thorough visual inspection was performed to verify the quality of the segmentation outputs regarding object size and shape. The same thresholds were applied to a given cell type across all tissue samples, regardless of the donors, including the size thresholds for nucleus and cytoplasm. Only the copy intensity parameter was adjusted by individual tissue sections to address staining variations of *AKT3* transcripts between samples, using their average cell intensity of RNAScope/IF signals. In the Circle sections, *GAD1* and DAPI were classified as IF probes, while GFAP, *AKT3*, and CLDN5 were classified as FISH probes. In the Star sections, *SLC17A7*, OLIG2, and DAPI were classified as IF dyes, while *AKT3* and TMEM119 were classified as FISH probes. For Circle sections, 7 phenotypes were used: DAPI/*AKT3*, *GAD1*/*AKT3*, GFAP/*AKT3*, CLDN5/*AKT3*, *GAD1*, GFAP, and CLDN5. For STAR sections, 7 phenotypes were used: DAPI/*ATK3*, OLIG2/*AKT3*, *SLC17A7*/*AKT3*, TMEM119/*AKT3*, OLIG2, *SLC17A7*, and TMEM119. Detailed information on the parameters used for segmentation, phenotyping, and quantification by the HALO algorithms can be accessed through the HALO settings files, which are available on GitHub at https://github.com/LieberInstitute/Human_DLPFC_Deconvolution/tree/main/raw-data/HALO/settings_files [58]. Output files with additional cell measurements are also located on GitHub https://github.com/LieberInstitute/Human_DLPFC_Deconvolution/tree/main/raw-data/HALO [58].

Due to the fragility of tissue sections following the RNAScope/IF procedure, the quality of tissue morphology and IF staining for each tissue section was evaluated by three microscopy experts using a scale of Low, Okay, or High. Low samples contained tears, folds, or missing segments of tissue. A subset of these samples also showed poor staining quality and overexposure during imaging leading to inaccurate segmentation. Okay samples contained some imperfections in morphology or staining quality but were overall intact tissue sections. High samples displayed superior morphology and staining quality. For the Star combination, this resulted in 8 Low, 9 Okay, and 3 High; and for Circle combination 9 Low, 3 Okay, and 9 High. Samples with Low overall quality were excluded from the analysis. The final number of tissue sections that passed morphological and staining quality control was n=12 for Circle and n = 13 for Star combinations (Fig S1B). In the High quality, a total of 1,077,136 cells were segmented. Based on the average expected size of cell, cells were excluded if their radius exceeded 5μm. This excluded 31,539 cells (2.3%). In the filtered dataset, the median number of cells in a tissue section was 40,035 (range 28,093:57,674).

### Cell Type Proportion Calculation

RNAScope/IF cell type proportions were calculated by dividing the number of nuclei for a given cell type by the total number of nuclei segmented in that tissue section (Circle = 32,425:57,674, Star 28,093:53,709) (Figure 2D, Table S3). Cell type proportions were calculated the same way for snRNA-seq data. RNAScope/IF proportions were compared to snRNA-seq proportions when a sample had data for both assays (Star n=12, Circle n=11) with Pearson correlation and root mean squared error (rmse) from *Metrics* v0.1.4 [59], as well as relative-rmse to the RNAScope/IF proportions (rrmse: rmse/mean( RNAScope/IF prop)) (Figure 2E).

### Marker Genes

*Mean Ratio* statistics were calculated with getMeanRatio2() and genes were selected with the rank_ratio metric. 1vALL statistics were calculated by findMarkers_1vAll(), both functions are from *DeconvoBuddies* v0.99.0 [60]. findMarkers_1vAll() is a wrapper function for findMarkers() from *scran* v1.26.2 [39] that for each cell type performs *t*-Student tests between a target cell type and all other cell types (Figure 3, Fig S6, Table S4). The top 25 *Mean Ratio* genes for each cell type were selected as the set of cell type marker genes for deconvolution.

### Gene Expression Visualization

Violin plots were created with plot_gene_express() from *DeconvoBuddies* v0.99.0 (Figure 3D). Heatmaps were plotted with *ComplexHeatmap* v2.18.0 (Figure 3E–F, Fig S6) [61].

### Deconvolution

Deconvolution was completed on the 110 bulk RNA-seq samples, using the snRNA-seq data as the reference at the broad cell type level. The reference dataset was subset to the top 25 *Mean Ratio* cell type marker genes that were common with the bulk RNA-seq dataset (151 genes, Table S4). The following deconvolution methods were applied to the data using the following software packages and functions. Unless noted, default parameters were used (Fig S7).

- *DWLS* v0.1.0, [5]
  - Functions: buildSignatureMatrixMAST(), trimData(),olveDampenedWLS
- *BisqueRNA* v1.0.5, [6]
  - Functions: ReferenceBasedDecomposition(use.overlap = FALSE)
- *MuSiC* v1.0.0, [7]
  - Functions: music_prop()
- *BayesPrism* v2.1.1, [8]
  - Functions: cleanup.genes(), select.gene.type(), get.exp.stat(), select.marker(), new.prism(), run.prism()
- *hHspe* v0.1, [9]
  - Function: hspe()

Correlation accuracy was evaluated against RNAScope/IF quantification for the tissue blocks with available data (n=12 or 13, see Methods: *RNAScope/Immunofluorescence data generation and HALO analysis*). Pearson’s correlation (cor) calculated with cor from *stats* [62], and root mean squared error (rmse), calculated by rmse() from *Metrics* v0.1.4 [59], were computed for all RNA-seq samples (Figure 4A), and for samples grouped by RNA extraction and library preparation (Fig S8, Figure 4B).

To compare the output from all five deconvolution methods to each other, and to RNAScope/IF plus snRNA-seq proportions, a pairwise scatter plot matrix was created using ggpairs() from *GGally* v2.2.0 [63] (Fig S9).

### Software

*ggplot2* v3.4.3 and earlier versions [64], *R* versions 4.2, and 4.3 [62], and *Bioconductor* versions 3.14, 3.16, and 3.18 were used for the analyses [65].

## Supplementary Material

Supplement 1

2

## Figures and Tables

**Table 1:** Selected Deconvolution Methods.

Method	Citation	Approach	Marker Gene Selection	Availability	Top Benchmark Performance
**DWLS** (Dampened weighted least-squares)	Tsoucas et al, Nature Comm, 2019 [5]	weighted least squares	-	R package on CRAN	Cobos et al. [18]
**Bisque**	Jew et al, Nature Comm, 2020 [6]	Bias correction: Assay	-	R package on GitHub	Dai et al. [17]
**MuSiC** (Multi-subject Single-cell)	Wang et al, Nature Communications, 2019 [7]	Bias correction: Source	Weights Genes	R package GitHub	Jin et al. [20]
**BayesPrism**	Chu et al., Nature Cancer, 2022 [8]	Bayesian	Pairwise t-test	Webtool R package on GitHub	-
**hspe** (**dtangle**) (hybrid-scale proportion estimation)	Hunt and Gagnon-Bartsch, Ann. Appl. Stat. 2021 [9, 43]	High collinearity adjustment	Multiple options-default “ratio” 1vALL mean expression ratio	R package on GitHub	Dai et al. [17]

The five reference based deconvolution methods selected for the benchmark analysis rely on different mathematical approaches, and marker gene selection strategies. Also noted is the software availability and other benchmark studies where these methods were noted as top performers.

## Data Availability

As documented previously [38], snRNA-seq FASTQ files are available via Globus endpoint ‘jhpce#DLPFC_snRNAseq’ endpoint listed at http://research.libd.org/globus as well as the PsychENCODE Knowledge Portal (https://PsychENCODE.synapse.org/) through https://www.synapse.org/#!Synapse:syn51032055/datasets/ [66]. Bulk RNA-seq FASTQ files and HALO raw images are available via the Globus endpoints ‘jhpce#humanDeconvolutionBulkRNAseq’ and ‘jhpce#humanDeconvolutionRNAScope’, respectively. The HALO exported setting files and data CSV files are available at https://github.com/LieberInstitute/Human_DLPFC_Deconvolution/tree/main/raw-data/HALO. The combined HALO output data is available into an R object is available at https://github.com/LieberInstitute/Human_DLPFC_Deconvolution/blob/main/processed-data/03_HALO/halo_all.Rdata. Source code for this project is available at https://github.com/LieberInstitute/Human_DLPFC_Deconvolution [58]. The *Mean Ratio* method is implemented in the R package *DeconvoBuddies* and is available at https://github.com/LieberInstitute/DeconvoBuddies [60].
