# Supplementary material for: Benchmark of cellular deconvolution methods using a multi-assay reference dataset from postmortem human prefrontal cortex
Source: bioRxiv. 2024 Apr 7:2024.02.09.579665. Originally published 2024 Feb 12. Preprint. [Version 2] doi: 10.1101/2024.02.09.579665 (PMC10888823; doi:10.1101/2024.02.09.579665)
Supplement: 1 [file NIHPP2024.02.09.579665V2-supplement-1.pdf]

bioRxiv preprint doi: https://doi.org/10.1101/2024.02.09.579665; this version posted April 7, 2024. The copyright holder for this preprint (which was not certified by peer review) is the author/funder, who has granted bioRxiv a license to display the preprint in perpetuity. It is made available under aCC-BY 4.0 International license.

Supplemental Figures

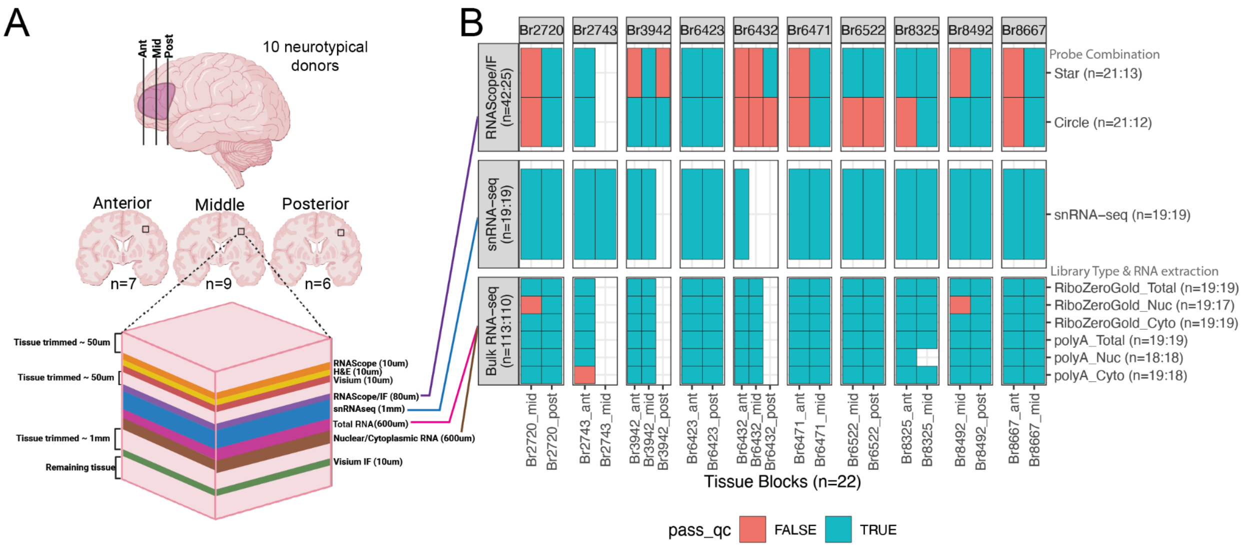

Fig S1: Schematic of assays performed on each tissue block. A. Schematic of DLPFC dissections and DLPFC tissue block position depicting order of assays completed (n=10 donors; n=22 tissue blocks, including 7 Anterior, 9 Middle, 6 Posterior). Approximately 50 µm of tissue was trimmed to achieve a flat surface for cryosectioning. Next, several ~10 µm sections were collected for anatomical validation (RNAScope/IF, H&E) and Visium experiments [40]. Blocks were stored at -80°C until completion of these assays. At the next cryostat session, blocks were trimmed and ~1 mm of tissue was collected for snRNA-seq (n=19) [40], ~600 µm of tissue was collected for Total RNA extraction for bulk-RNAseq, and ~600 µm of tissue was collected for fractionated RNA extraction for nuclear (Nuc) and cytoplasmic (Cyto) RNA-seq. Finally, four tissue blocks were placed back on the cryostat and trimmed again to obtain a flat surface prior to collecting a ~10 µm Visium-spatial proteogenomics (SPG) tissue section [40]. **B**. Tile plot illustrating which assays and configurations (probe/antibody combination for RNAScope/IF and library type/RNA extraction for RNA-seq) were performed on each tissue block and the sample size for that assay before and after quality control (qc) in the format “(n=before:after)”. The tile is blank if an assay configuration was not performed on the tissue block. The tile is blue if the sample passed qc checks and was included in the analysis. Red tiles are not included in the study.

bioRxiv preprint doi: https://doi.org/10.1101/2024.02.09.579665; this version posted April 7, 2024. The copyright holder for this preprint (which was not certified by peer review) is the author/funder, who has granted bioRxiv a license to display the preprint in perpetuity. It is made available under aCC-BY 4.0 International license.

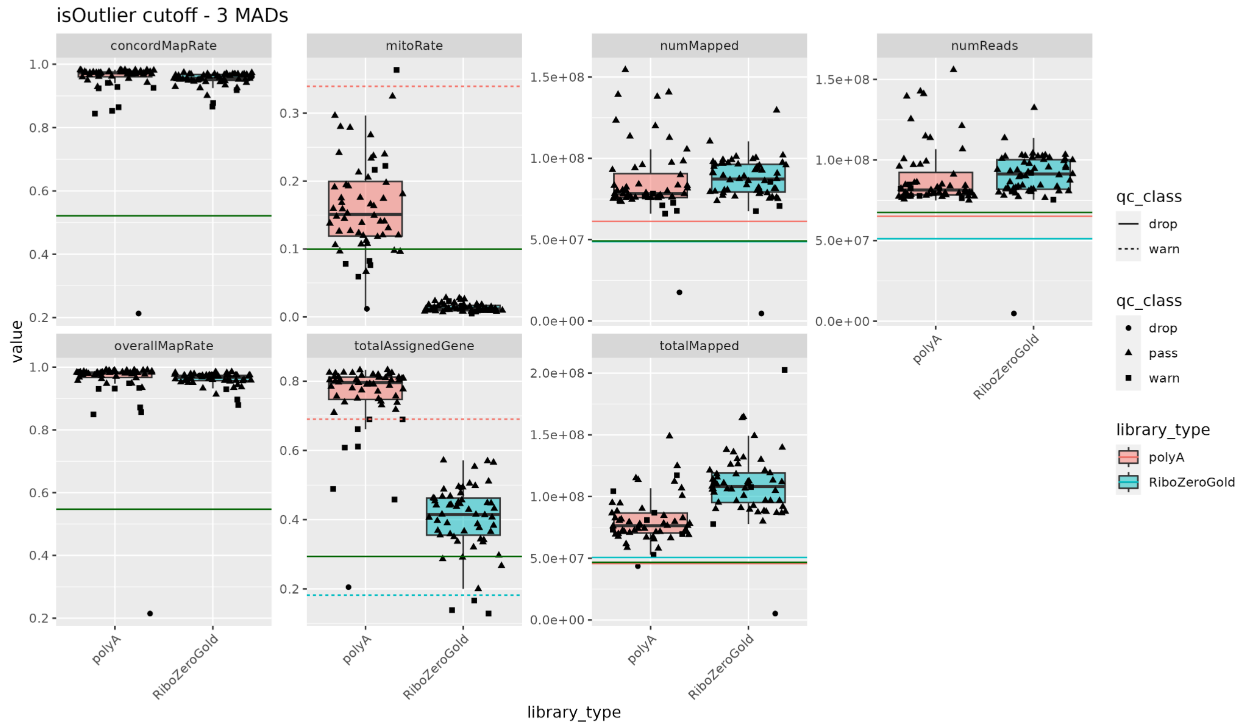

Fig S2: Bulk RNA-seq data Quality Control. Samples are evaluated for low `concordMapRate`, `numMapped`, `numReads`, `overallMapRate`, `totalAssignedGene`, and `totalMapped` or high `mitoRate`. See the *SPEAQeasy* [61] documentation at https://research.libd.org/SPEAQeasy/outputs.html#quality-metrics for the definition of these variables. Cutoffs (horizontal lines) were determined by a 3 median absolute deviations from the mean (3 MADs) from the distributions for the polyA or RiboZeroGold samples (line color) using isOutlier() from *scran* [41], as well as historic cutoffs from previous LIBD bulk RNA-seq projects (green lines). Based on the distribution of the values, and logic with the QC metrics some were "warning" cutoffs vs. "drop" cutoffs (line type). RNA-seq samples were classified as "drop", "warn", or "pass" based on their relationship to the cutoffs.

bioRxiv preprint doi: https://doi.org/10.1101/2024.02.09.579665; this version posted April 7, 2024. The copyright holder for this preprint (which was not certified by peer review) is the author/funder, who has granted bioRxiv a license to display the preprint in perpetuity. It is made available under aCC-BY 4.0 International license.

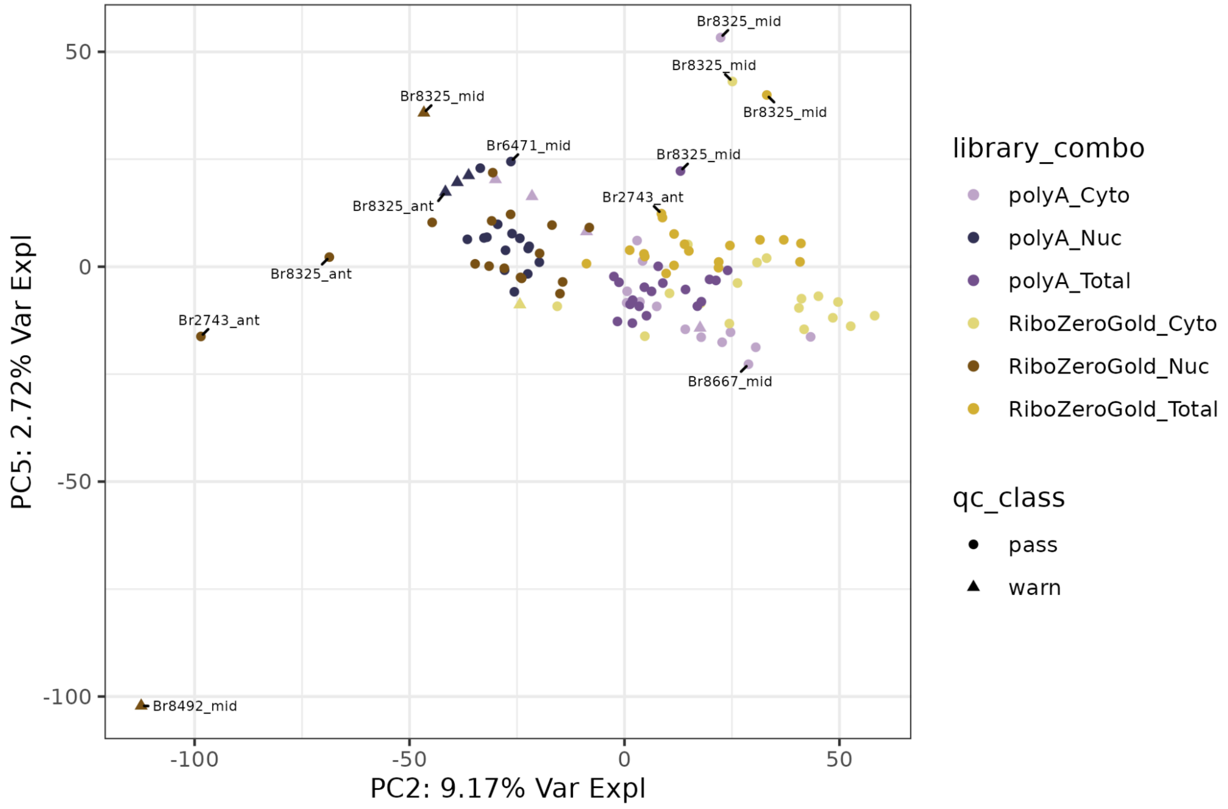

Fig S3: Bulk RNA-seq data Quality Control Principal Component Analysis (PCA). PC2 versus 5 colored by "library combo" (library type + RNA extraction). The sample `AN00000906_Br8492_Mid_Nuc` was identified as an outlier and removed from downstream analysis.

bioRxiv preprint doi: https://doi.org/10.1101/2024.02.09.579665; this version posted April 7, 2024. The copyright holder for this preprint (which was not certified by peer review) is the author/funder, who has granted bioRxiv a license to display the preprint in perpetuity. It is made available under aCC-BY 4.0 International license.

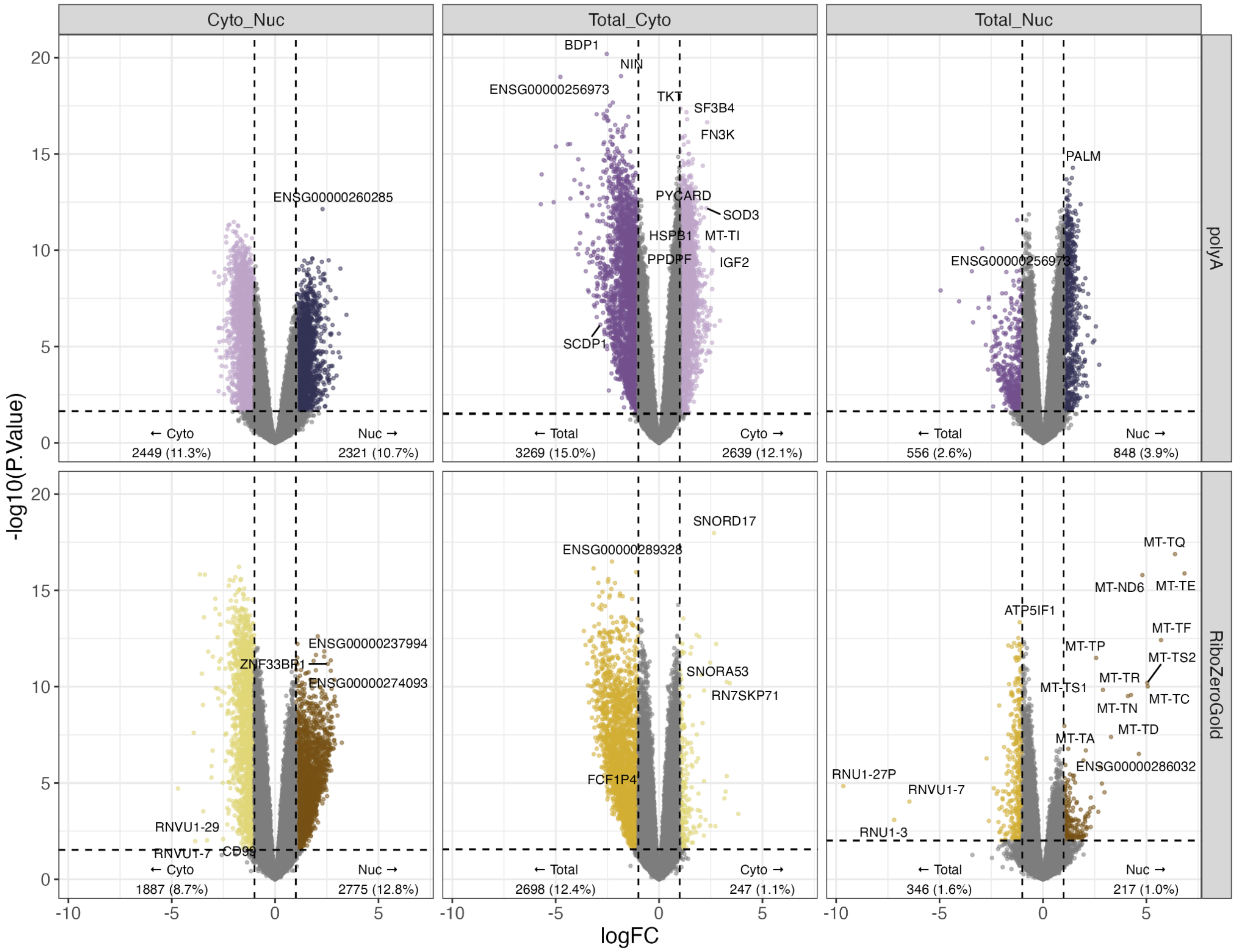

Fig S4: Volcano plots for RNA extraction Differential Gene Expression analysis. Samples were separated by library types (rows) and the RNA extractions: cytosolic (Cyto, light color), total cell (Total, intermediate color), or nuclear (Nuc, dark color) samples were compared by differential expression in a pair-wise fashion (columns). Related to **Figure 1E**.

bioRxiv preprint doi: https://doi.org/10.1101/2024.02.09.579665; this version posted April 7, 2024. The copyright holder for this preprint (which was not certified by peer review) is the author/funder, who has granted bioRxiv a license to display the preprint in perpetuity. It is made available under aCC-BY 4.0 International license.

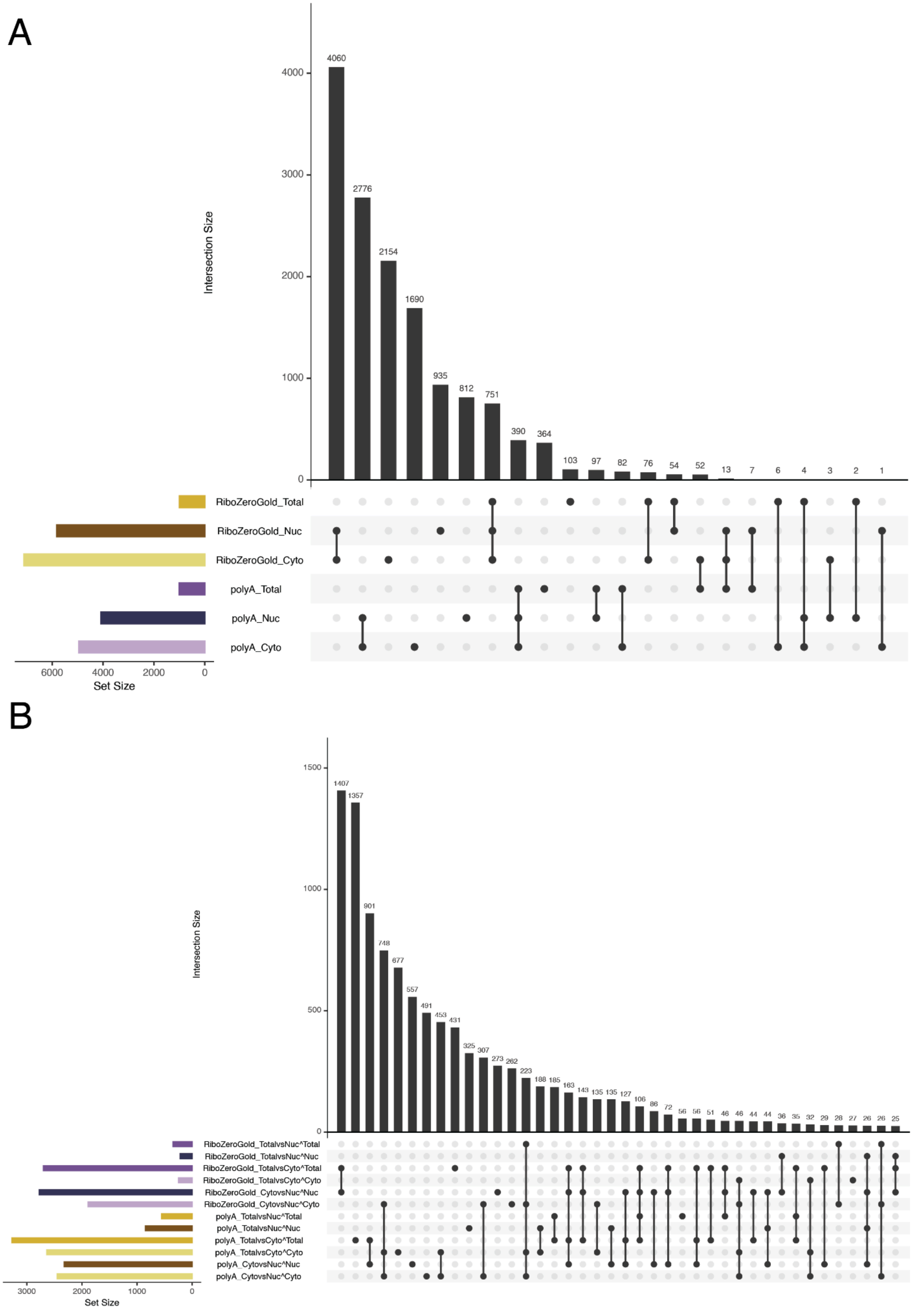

bioRxiv preprint doi: https://doi.org/10.1101/2024.02.09.579665; this version posted April 7, 2024. The copyright holder for this preprint (which was not certified by peer review) is the author/funder, who has granted bioRxiv a license to display the preprint in perpetuity. It is made available under aCC-BY 4.0 International license.

Fig S5: Upset plots for Differentially Quantified Genes. Displays the overlap between sets of differentially quantified genes for tests between **A.** library type (ex. RiboZeroGold_Total vs. polyA_Total) or **B.** RNA extraction (ex. RiboZeroGold_Total vs. RiboZeroGold_Cyto, where over quantified genes in Total would be notated as RiboZeroGold_TotalvsCyto^Total). Left barplots are colored by the combination of the library preparation and RNA extraction the set of genes are over quantified in. Related to **Figure 1E**, **Fig S4**.

bioRxiv preprint doi: https://doi.org/10.1101/2024.02.09.579665; this version posted April 7, 2024. The copyright holder for this preprint (which was not certified by peer review) is the author/funder, who has granted bioRxiv a license to display the preprint in perpetuity. It is made available under aCC-BY 4.0 International license.

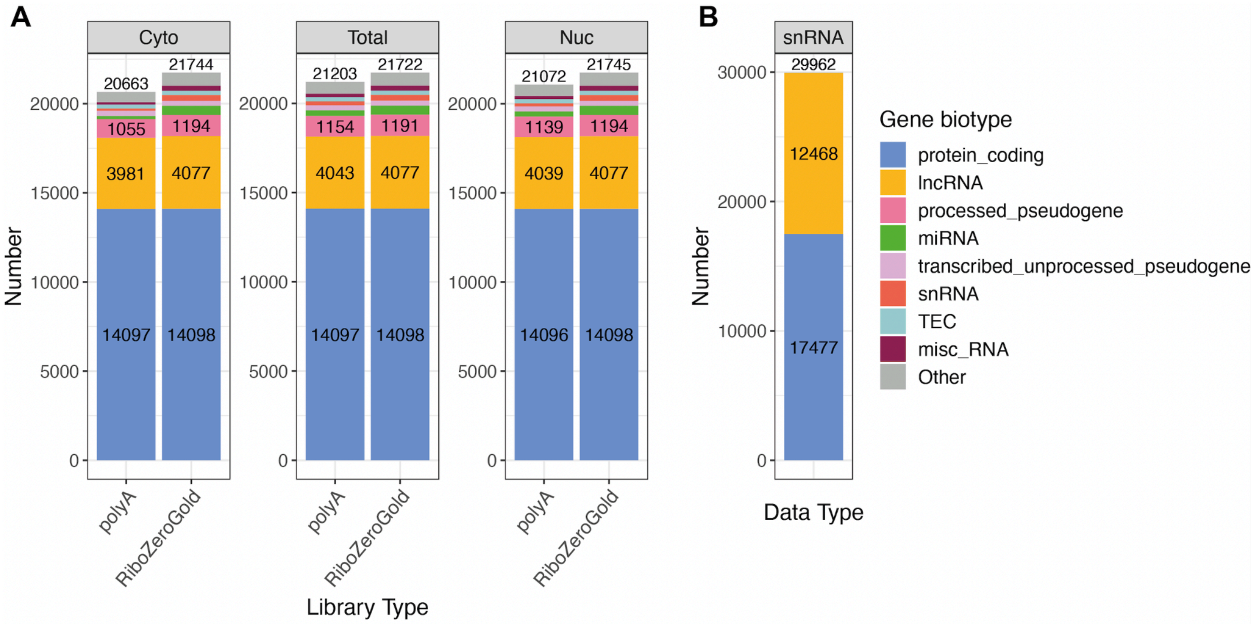

Fig S6: Biotypes of expressed genes in bulk and snRNA-seq datasets. The total number of expressed genes (after removing lowly expressed genes) and their biotypes in **A**. each of the 6 bulk RNA-seq libraries, comparing polyA vs RiboZeroGold in cytoplasmic, total, and nuclear RNA samples, and in **B**. snRNA-seq samples. These are the genes used as input for the DQG analysis. Related to **Figure 1**.

bioRxiv preprint doi: https://doi.org/10.1101/2024.02.09.579665; this version posted April 7, 2024. The copyright holder for this preprint (which was not certified by peer review) is the author/funder, who has granted bioRxiv a license to display the preprint in perpetuity. It is made available under aCC-BY 4.0 International license.

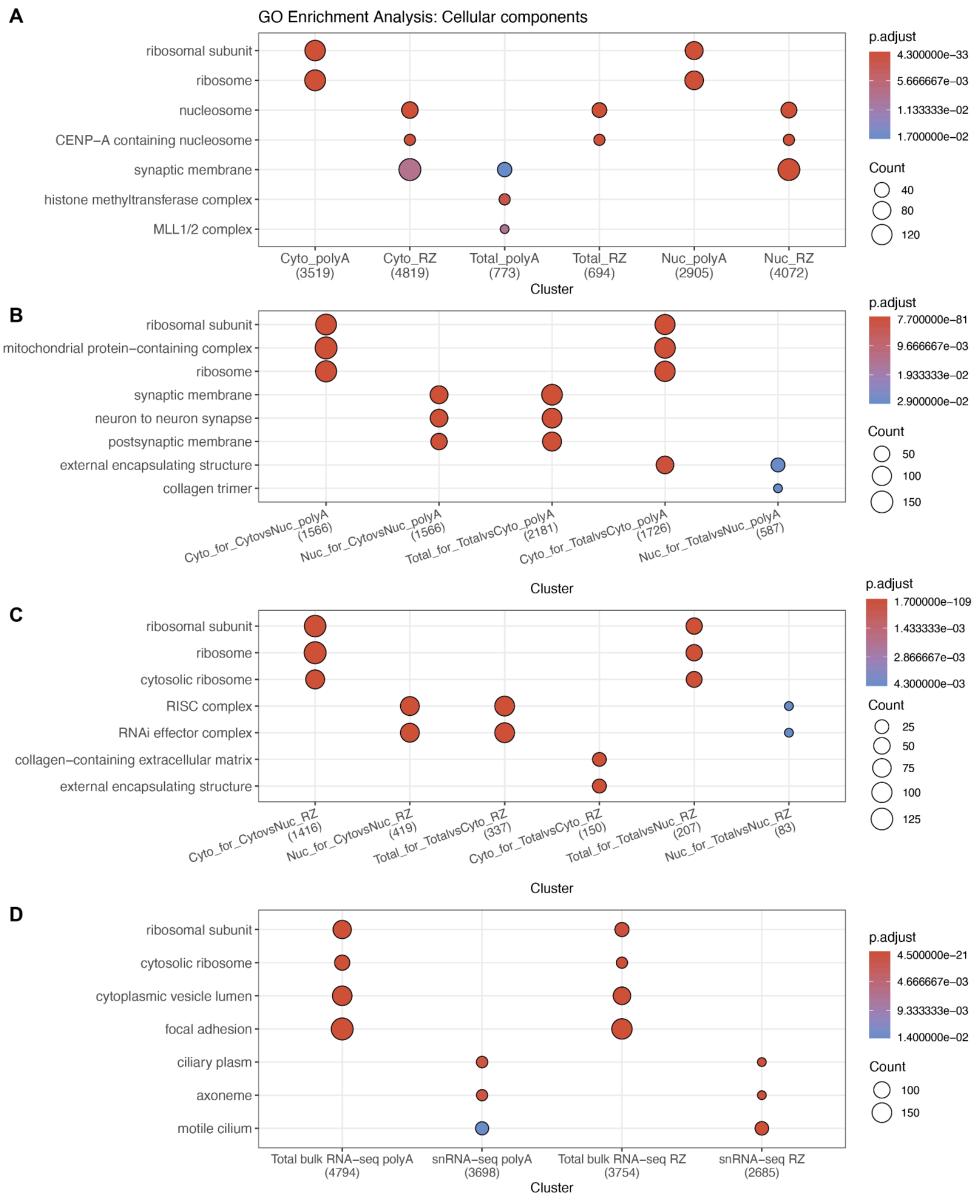

Fig S7: Enrichment of gene ontology cellular component terms in DQGs. Comparison of gene sets defined by either (1; Y-axis) having common cellular components (CC) in the Gene Ontology (GO) knowledgebase, or (2; X-axis) being significantly differentially quantified (FDR<0.05) between **A**. library types in the same RNA fractions, RNA extractions in the same library types for **B**. polyA and **C**. RiboZeroGold, respectively, and **D**. sequencing assay types (snRNA-seq compared against Total RNA-seq). Gene clusters without significant enrichments are excluded. The numbers below each DQG group (X-axis) correspond to the number of genes in each group that are also annotated in the GO knowledgebase. Count is the size of the overlap between genes annotated in each CC GO term and the DQG groups. Only the top 2 most significant enriched CC GO terms per DQG group are shown; some of the CC GO terms overlap. Related to **Figure 1E-F**, **Fig S4**.

bioRxiv preprint doi: https://doi.org/10.1101/2024.02.09.579665; this version posted April 7, 2024. The copyright holder for this preprint (which was not certified by peer review) is the author/funder, who has granted bioRxiv a license to display the preprint in perpetuity. It is made available under aCC-BY 4.0 International license.

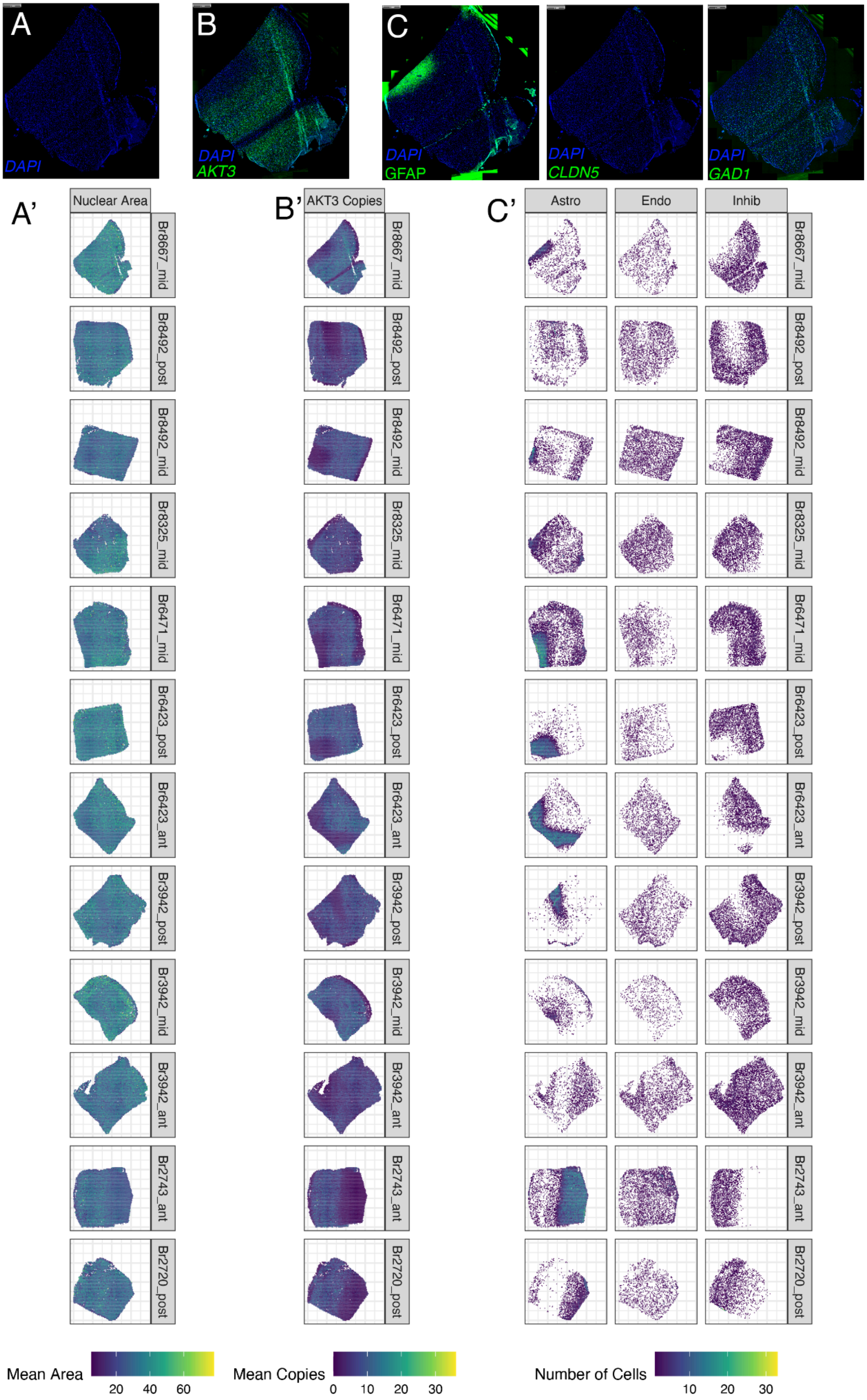

bioRxiv preprint doi: https://doi.org/10.1101/2024.02.09.579665; this version posted April 7, 2024. The copyright holder for this preprint (which was not certified by peer review) is the author/funder, who has granted bioRxiv a license to display the preprint in perpetuity. It is made available under aCC-BY 4.0 International license.

Fig S8: Representative fluorescence images and corresponding hex plots of all RNAScope/IF circle combination samples. Raw fluorescence for representative sample Br8667_mid: **A.** nuclear DAPI signals, **B.** DAPI and *AKT3*, **C.** DAPI and cell type probes/antibodies GFAP, CLDN5, and *GAD1*. Hex plots (`bins = 100`) from all circle samples summarizing: **A'.** mean nuclear area, **B'.** mean copies of *AKT3*, and **C'.** number of cells from the tagged cell types (Astro, Endo, and Inhib). Related to **Figure 2**.

bioRxiv preprint doi: https://doi.org/10.1101/2024.02.09.579665; this version posted April 7, 2024. The copyright holder for this preprint (which was not certified by peer review) is the author/funder, who has granted bioRxiv a license to display the preprint in perpetuity. It is made available under aCC-BY 4.0 International license.

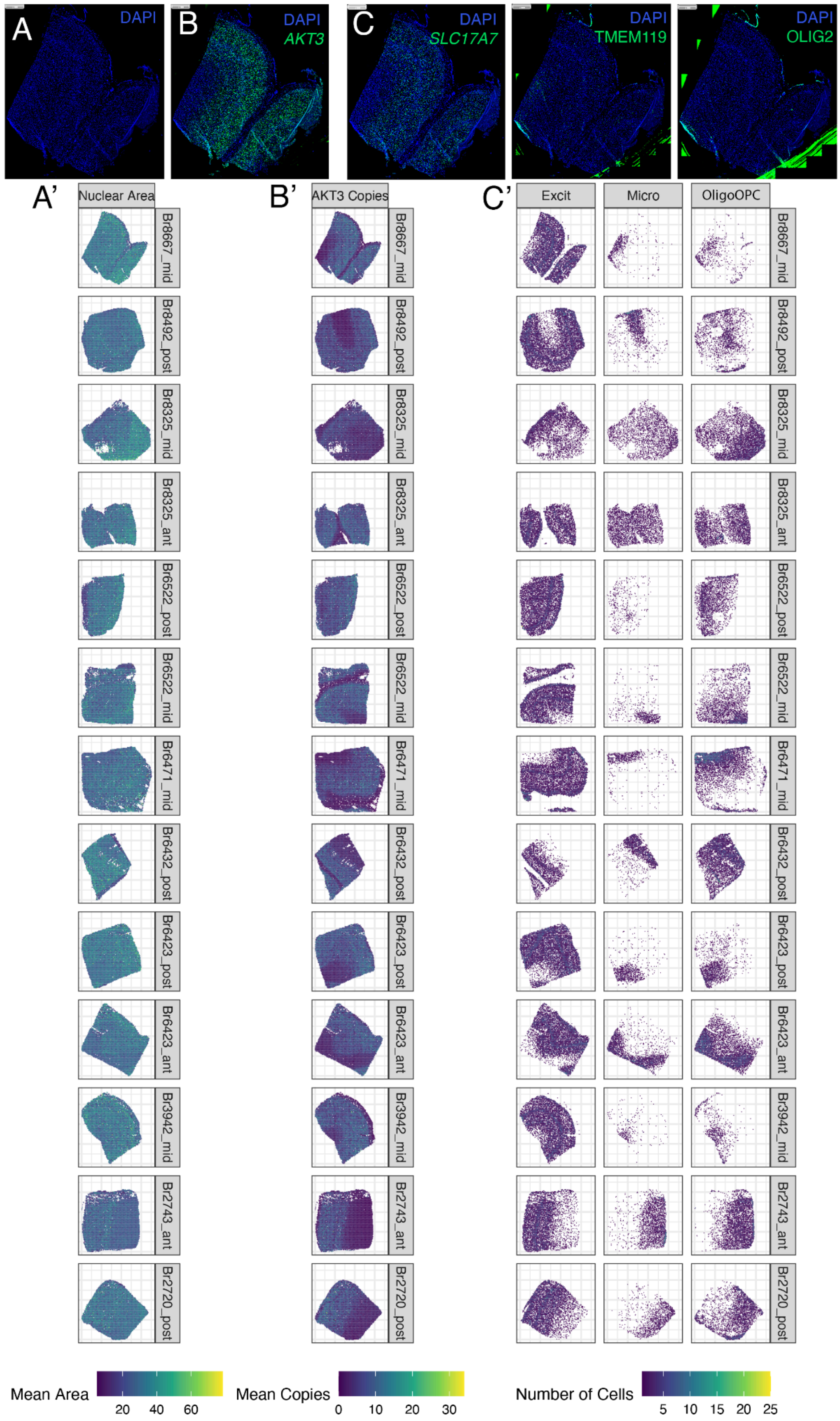

bioRxiv preprint doi: https://doi.org/10.1101/2024.02.09.579665; this version posted April 7, 2024. The copyright holder for this preprint (which was not certified by peer review) is the author/funder, who has granted bioRxiv a license to display the preprint in perpetuity. It is made available under aCC-BY 4.0 International license.

Fig S9: Representative fluorescence images and corresponding hex plots of all RNAScope/IF star combination samples. Raw fluorescence for representative sample Br8667_mid: **A.** nuclear DAPI signals, **B.** DAPI and *AKT3*, **C.** DAPI and cell type probes/antibodies *SLC17A7*, TMEM119, and OLIG2. Hex plots (`bins = 100`) from all circle samples summarizing: **A'.** mean nuclear area, **B'.** mean copies of *AKT3*, and **C'.** number of cells from the tagged cell types (Excit, Micro, and OligoOPC). Related to **Figure 2**.

bioRxiv preprint doi: https://doi.org/10.1101/2024.02.09.579665; this version posted April 7, 2024. The copyright holder for this preprint (which was not certified by peer review) is the author/funder, who has granted bioRxiv a license to display the preprint in perpetuity. It is made available under aCC-BY 4.0 International license.

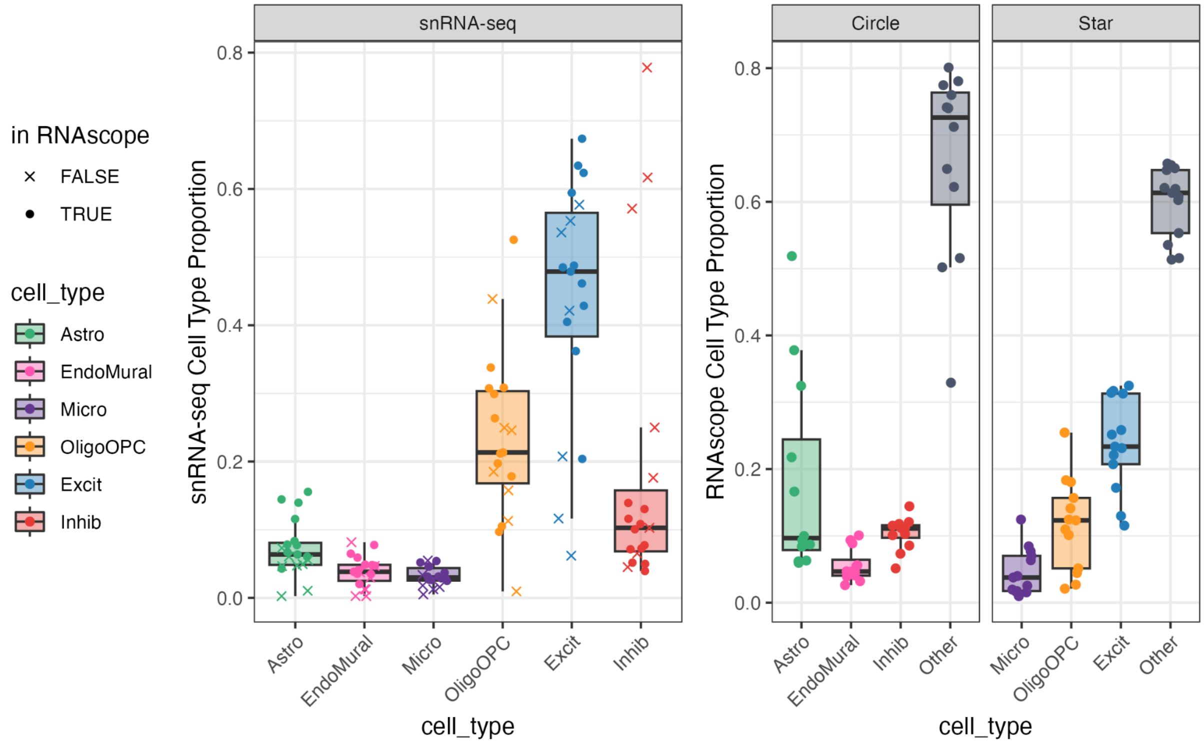

Fig S10: Boxplots of cell type proportions calculated from snRNA-seq and RNAScope/IF data. (**Left Side**) This side shows the snRNA-seq cell type proportions at the broad cell type resolution for all 19 tissue blocks for which snRNA-seq data was previously generated [40]. A filled dot marks tissue blocks for which RNAScope/IF data was also generated and passed quality control checks. Those absent are labeled with an “x”. (**Right Side**) This side shows the RNAScope/IF broad cell type proportions derived from the RNAScope/IF experiments for the Circle and Star combination of cell type markers. Cell type identities on the RNAScope/IF images were assigned using HALO (Indica Labs). Related to **Figure 2**.

bioRxiv preprint doi: https://doi.org/10.1101/2024.02.09.579665; this version posted April 7, 2024. The copyright holder for this preprint (which was not certified by peer review) is the author/funder, who has granted bioRxiv a license to display the preprint in perpetuity. It is made available under aCC-BY 4.0 International license.

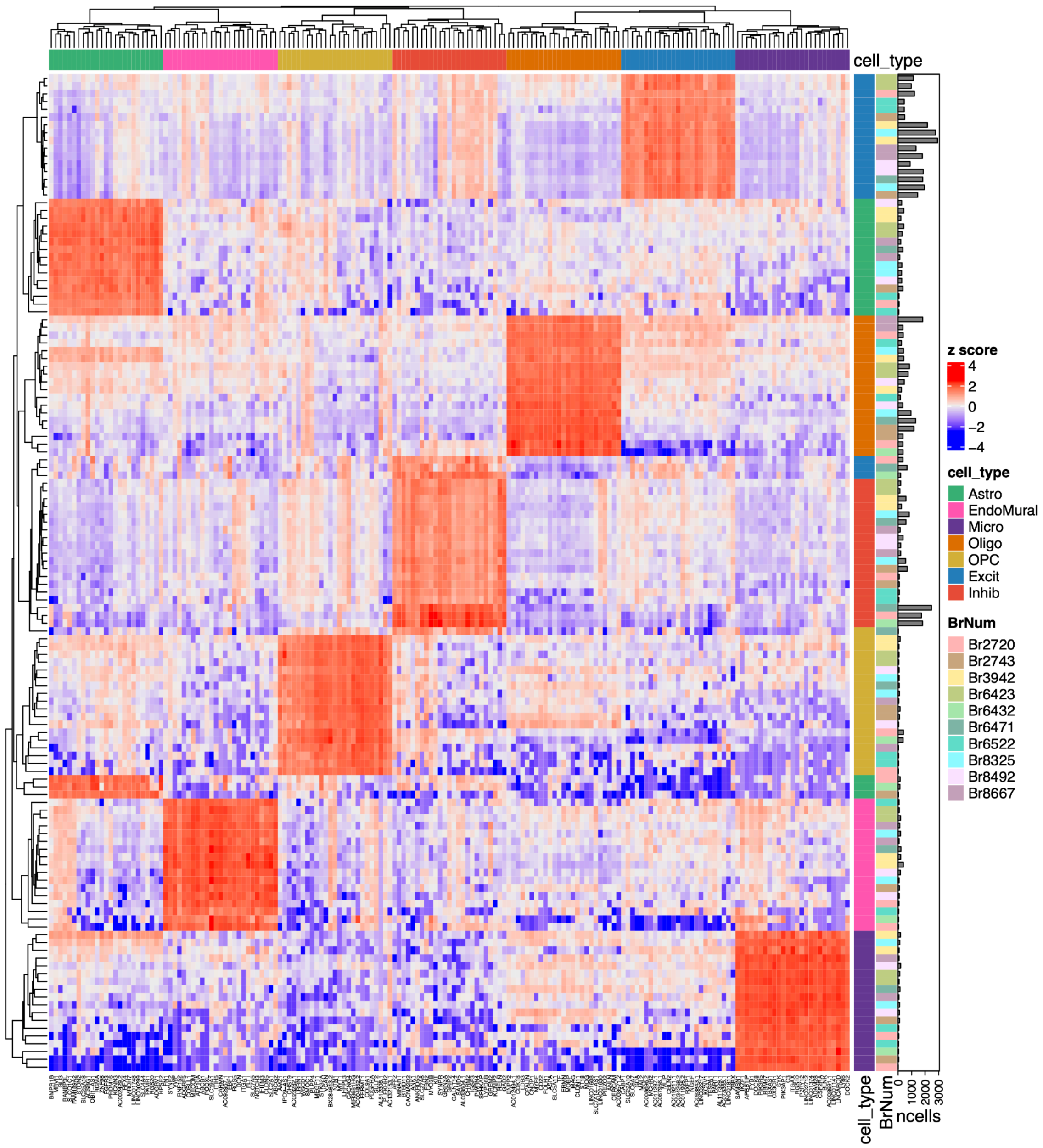

Fig S11: Heatmap of the *Mean Ratio top25* marker genes for deconvolution. Normalized snRNA-seq counts (logcounts) were centered and scaled by gene to compute Z scores. Brain donor identifiers (`BrNum`) and cell types were used to annotate this heatmap made with *ComplexHeatmap* [72]. Genes are shown in the columns and nuclei on the rows, with the total number of nuclei (ncells) visualized as side barplots. Related to **Figure 3F**.

bioRxiv preprint doi: https://doi.org/10.1101/2024.02.09.579665; this version posted April 7, 2024. The copyright holder for this preprint (which was not certified by peer review) is the author/funder, who has granted bioRxiv a license to display the preprint in perpetuity. It is made available under aCC-BY 4.0 International license.

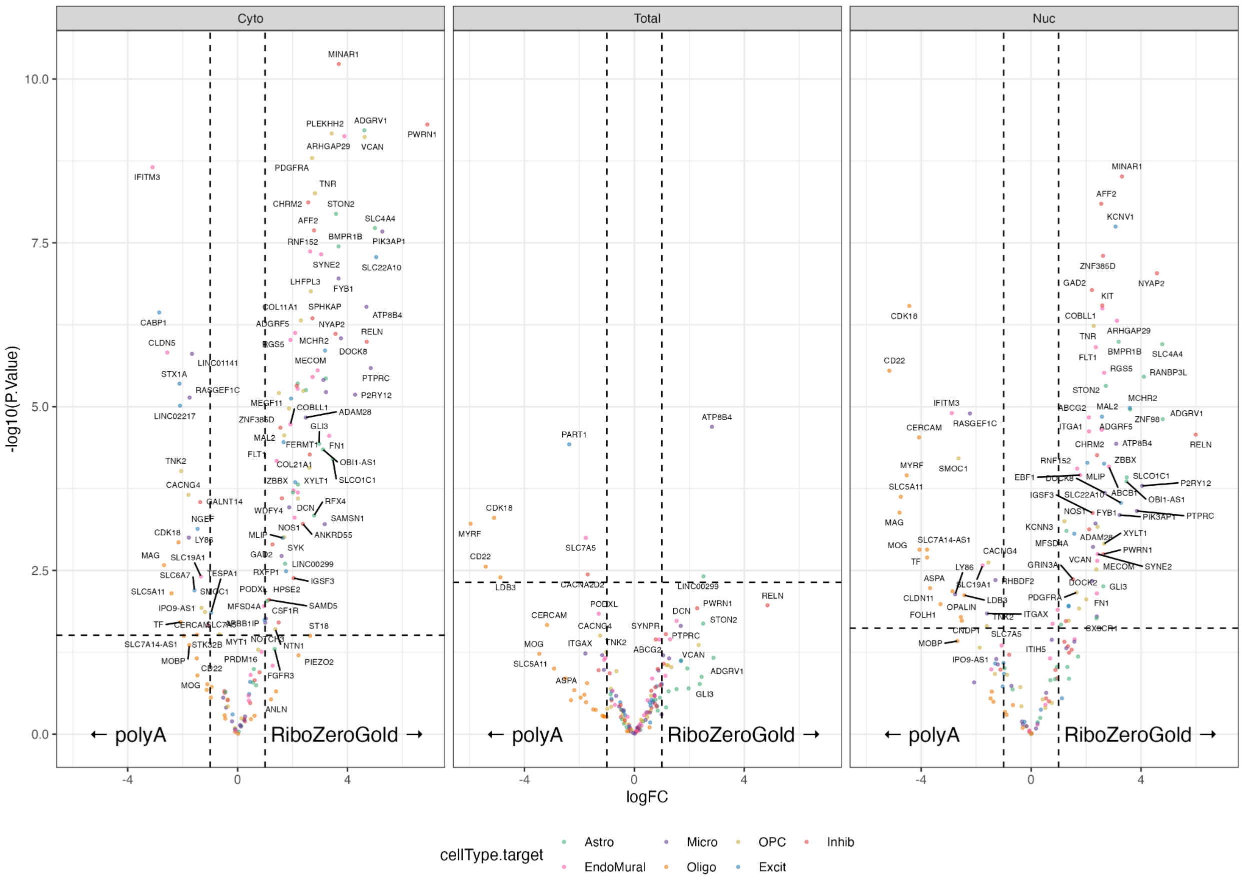

Fig S12: Volcano plots for library type Differential Gene Expression analysis filtered to *Mean Ratio top25* marker genes. Plots are faceted by RNA extraction methods. Horizontal dotted line denotes FDR < 0.05 cutoff, vertical dotted lines are logFC = -1 and 1. Related to **Figure 1E** and **Figure 3**.

bioRxiv preprint doi: https://doi.org/10.1101/2024.02.09.579665; this version posted April 7, 2024. The copyright holder for this preprint (which was not certified by peer review) is the author/funder, who has granted bioRxiv a license to display the preprint in perpetuity. It is made available under aCC-BY 4.0 International license.

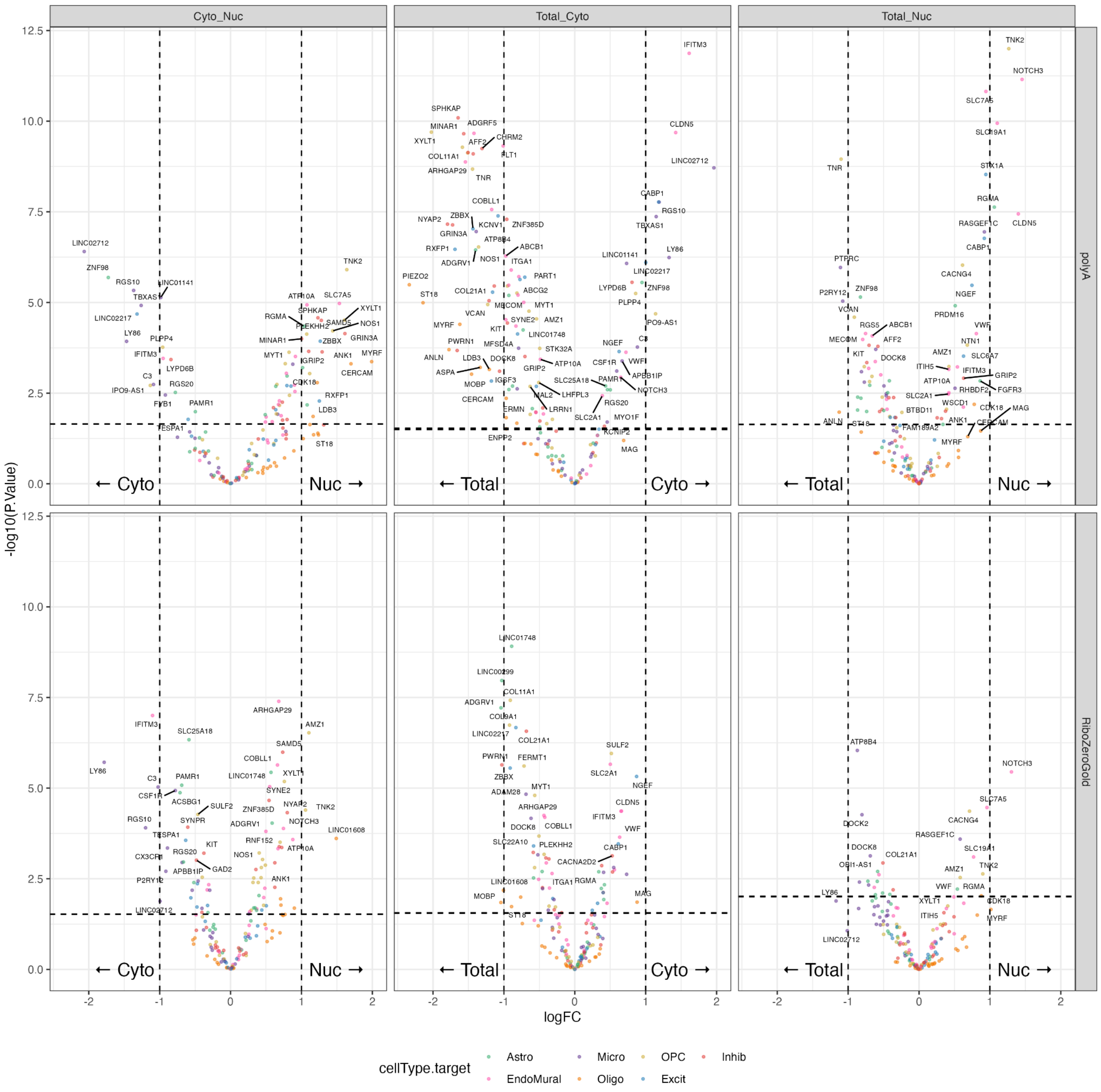

Fig S13: Volcano plots for RNA extraction Differential Gene Expression analysis filtered to Mean Ratio top25 marker genes. Plots are faceted by library preparation (rows), and pairwise comparisons (columns). Horizontal dotted line denotes FDR < 0.05 cutoff, vertical dotted lines are logFC = -1 and 1**.** Related to **Fig S4** and and **Figure 3**.

bioRxiv preprint doi: https://doi.org/10.1101/2024.02.09.579665; this version posted April 7, 2024. The copyright holder for this preprint (which was not certified by peer review) is the author/funder, who has granted bioRxiv a license to display the preprint in perpetuity. It is made available under aCC-BY 4.0 International license.

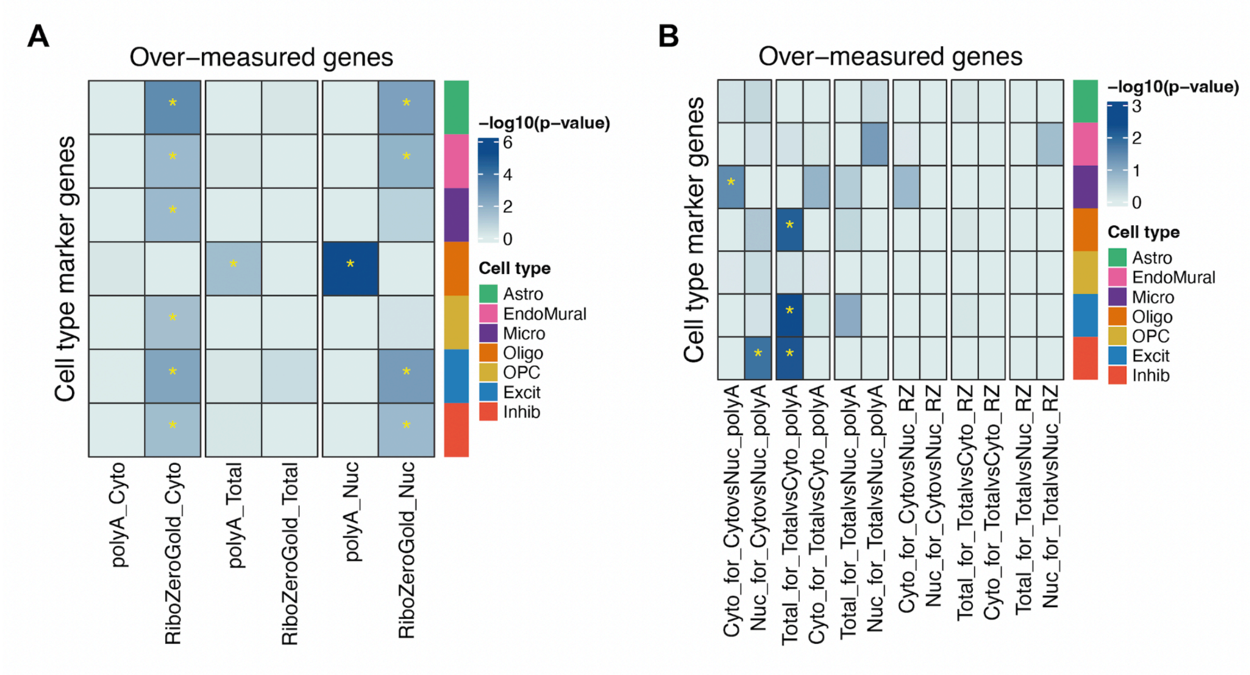

Fig S14: Over-quantification of cell type marker genes in library type and RNA fraction RNA-seq libraries. The over-representation of top 25 *Mean Ratio* cell type marker genes among DQG groups between (**A**) polyA and RibZeroGold RNA library types, and (**B**) between Cyto, Nuc, and Total RNA extractions. Over-representation was assessed with one-sided Fisher's exact tests. The *p*-values for such enrichments are shown in the heat maps in a negative log10 scale. Significant associations (*p*-value <0.05) are indicated with a yellow "*". Related to **Figure 1E, Fig S4**, and **Figure 3**.

bioRxiv preprint doi: https://doi.org/10.1101/2024.02.09.579665; this version posted April 7, 2024. The copyright holder for this preprint (which was not certified by peer review) is the author/funder, who has granted bioRxiv a license to display the preprint in perpetuity. It is made available under aCC-BY 4.0 International license.

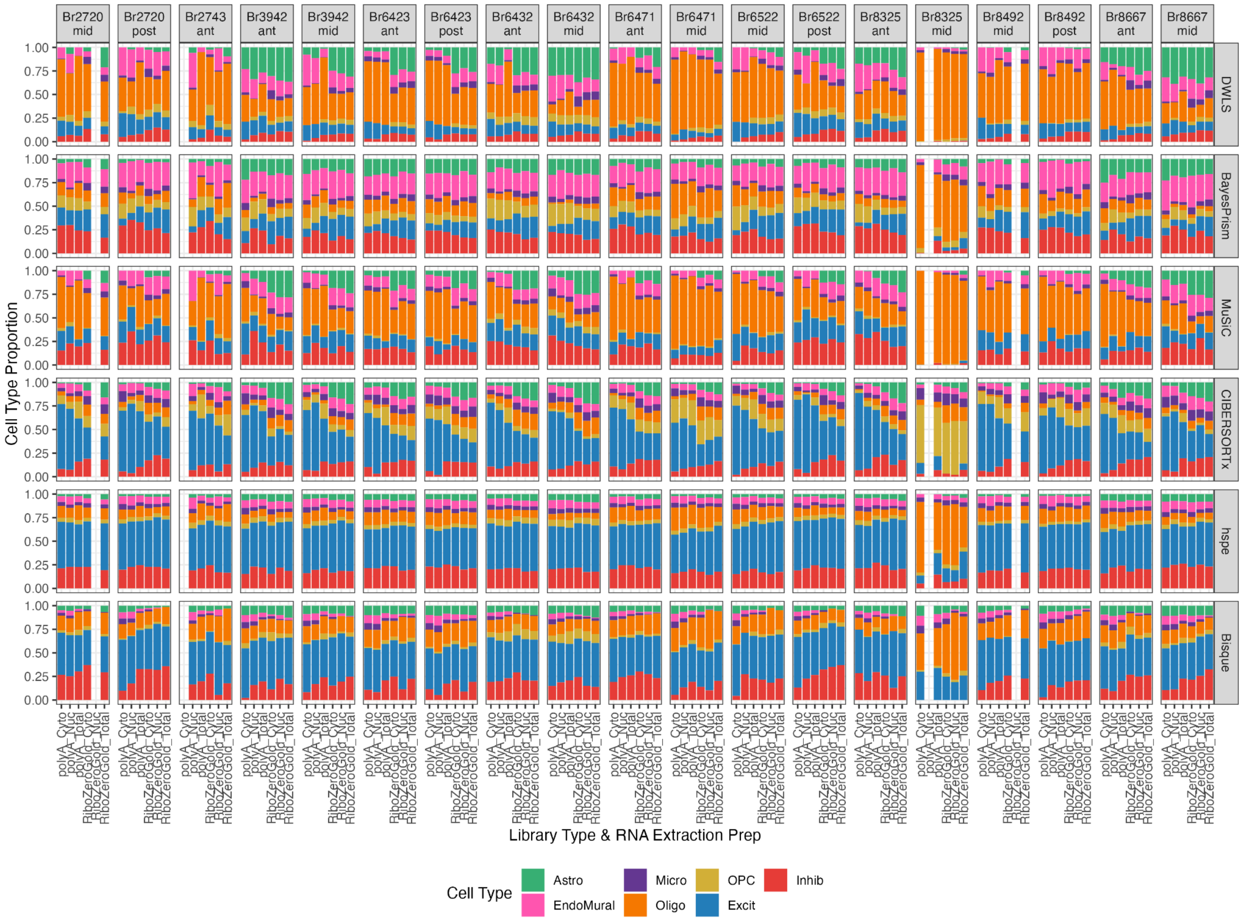

Fig S15: Barplots of estimated cell type proportions from deconvolution methods. Each tissue block is a column, the x-axis categories are the six RNA extraction and library type combinations for the 110 bulk RNA-seq samples. The rows are the predictions from each of the six deconvolution methods. Columns are labeled by the tissue block, which is a combination of the brain donor identifier (`BrNum`) and the anterior-posterior axis location of the tissue block (anterior: ant, middle: mid, or posterior: post). Related to **Figure 4**.

bioRxiv preprint doi: https://doi.org/10.1101/2024.02.09.579665; this version posted April 7, 2024. The copyright holder for this preprint (which was not certified by peer review) is the author/funder, who has granted bioRxiv a license to display the preprint in perpetuity. It is made available under aCC-BY 4.0 International license.

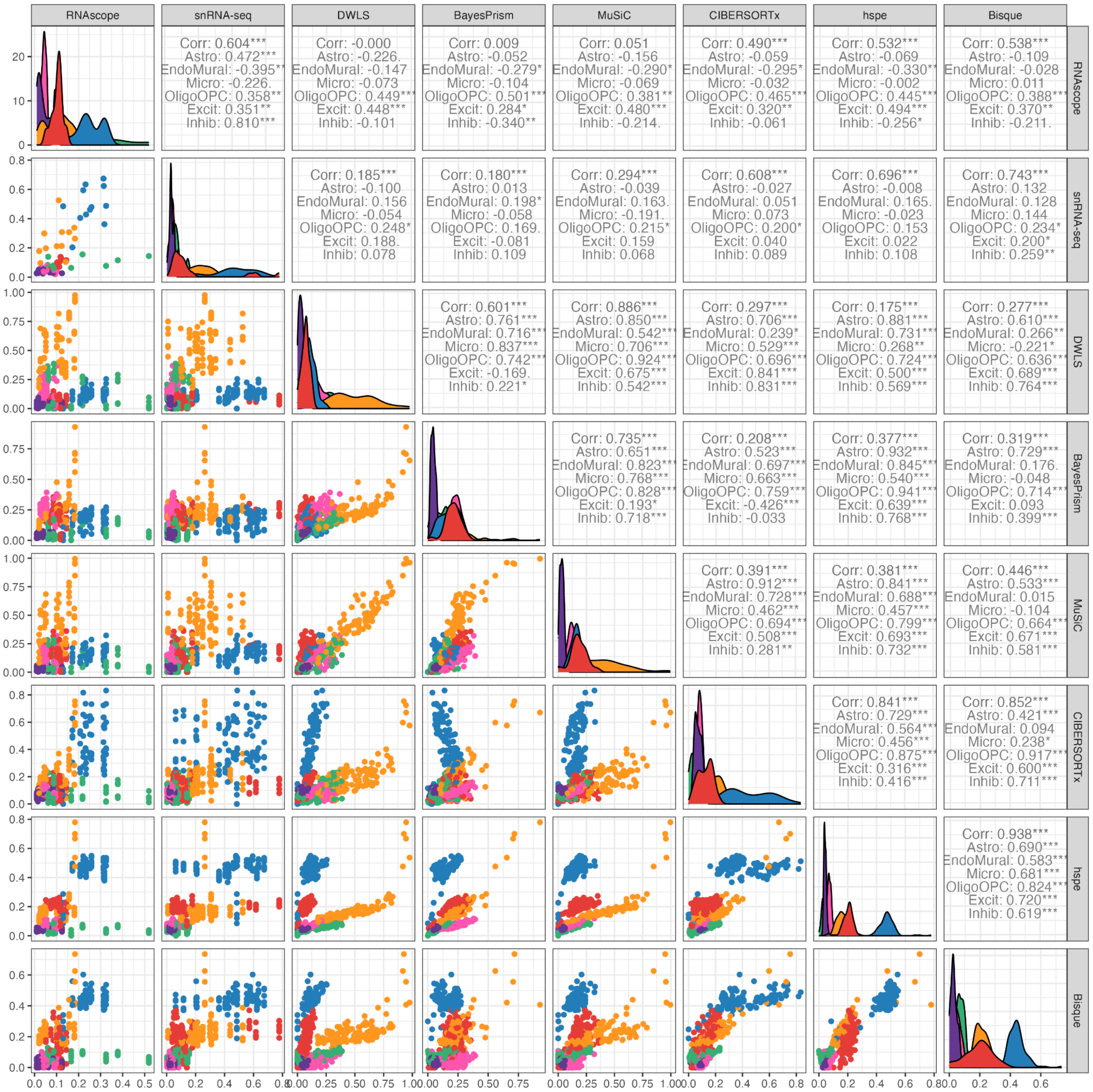

Fig S16: Cell composition comparison for Mean Ratio top25 results. Pairwise scatter plots of measured and estimated cell type proportions from the RNAScope/IF experiments, snRNA-seq data, and deconvolution methods using Mean Ratio top25 marker genes. Cell type proportions are colored by cell type and are shown in the lower triangle. Pearson correlation values (cor) calculated by `ggpairs()` from *GGally* [74] for each cell type are shown in the upper triangle. Density plots of the proportions are shown in the diagonal panels. Related to **Figure 4**.

bioRxiv preprint doi: https://doi.org/10.1101/2024.02.09.579665; this version posted April 7, 2024. The copyright holder for this preprint (which was not certified by peer review) is the author/funder, who has granted bioRxiv a license to display the preprint in perpetuity. It is made available under aCC-BY 4.0 International license.

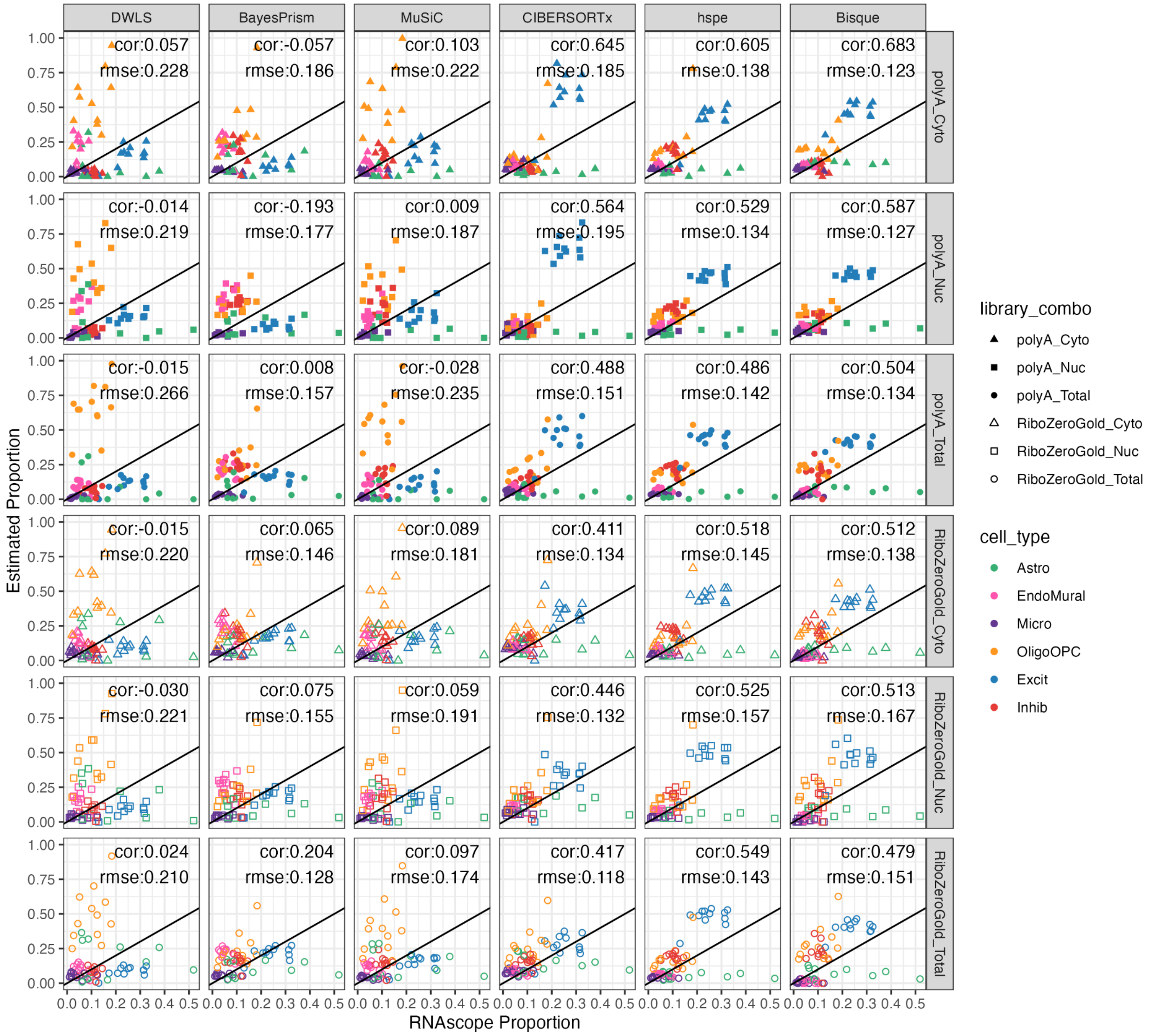

Fig S17: Cell composition results against RNAScope/IF across bulk RNA library type and RNA extractions. Scatter plots of cell type proportions by RNA library combinations (columns) and deconvolution methods (rows). Points are colored by cell type and shaped by the RNA library combination. Pearson correlation (cor) and root mean squared error (rsme) values are shown for each panel. Related to **Figure 4**.

bioRxiv preprint doi: https://doi.org/10.1101/2024.02.09.579665; this version posted April 7, 2024. The copyright holder for this preprint (which was not certified by peer review) is the author/funder, who has granted bioRxiv a license to display the preprint in perpetuity. It is made available under aCC-BY 4.0 International license.

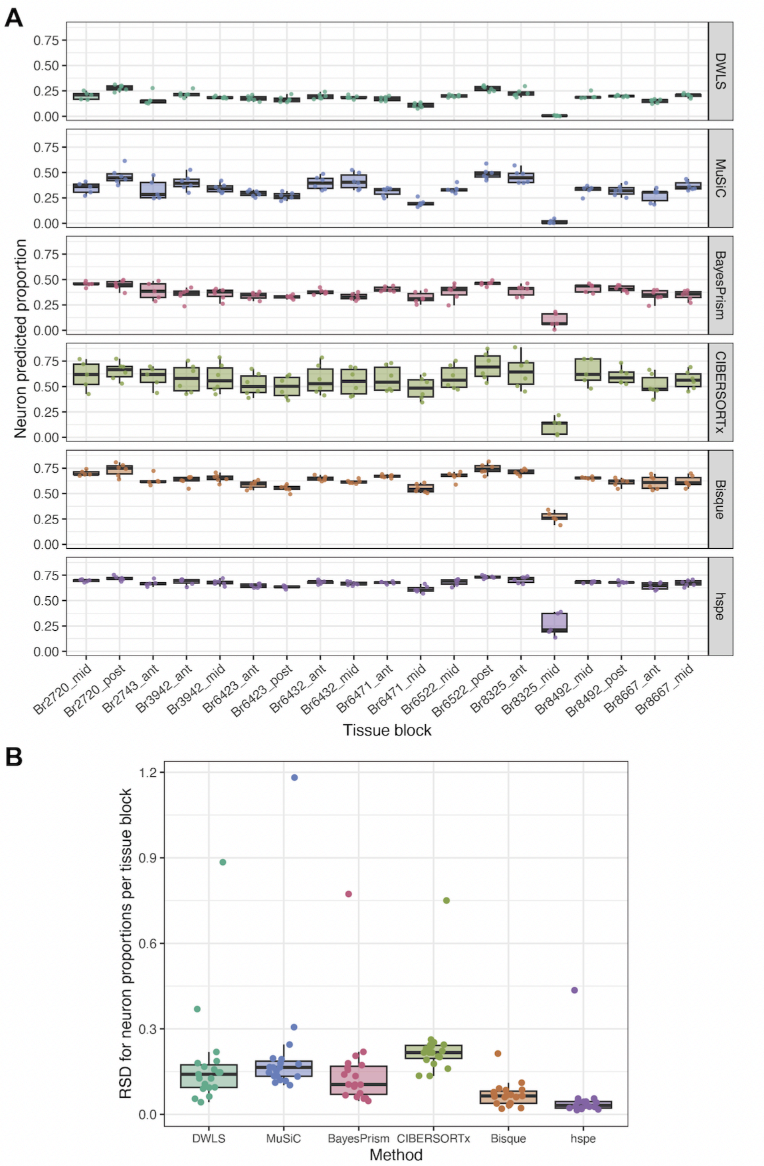

Fig S18: Variation in estimated neuron proportions across bulk RNA-seq samples from each tissue block. A. Boxplots for the proportion of inhibitory and excitatory neurons (neuron predicted proportion) estimated by each deconvolution method across the six combinations of RNA-seq library preparation types and RNA extraction. **B**. Boxplots of relative standard deviation (RSD, also known as coefficient of variation; RSD = CV = σ / μ) in each tissue block for the neuron predicted proportions by each deconvolution method. The high outlier value corresponds to the `Br8325_mid` tissue block RSD, for which more variable and lower neuron proportions were predicted by the methods (A). Related to **Figure 5** and **Fig S15**.

bioRxiv preprint doi: https://doi.org/10.1101/2024.02.09.579665; this version posted April 7, 2024. The copyright holder for this preprint (which was not certified by peer review) is the author/funder, who has granted bioRxiv a license to display the preprint in perpetuity. It is made available under aCC-BY 4.0 International license.

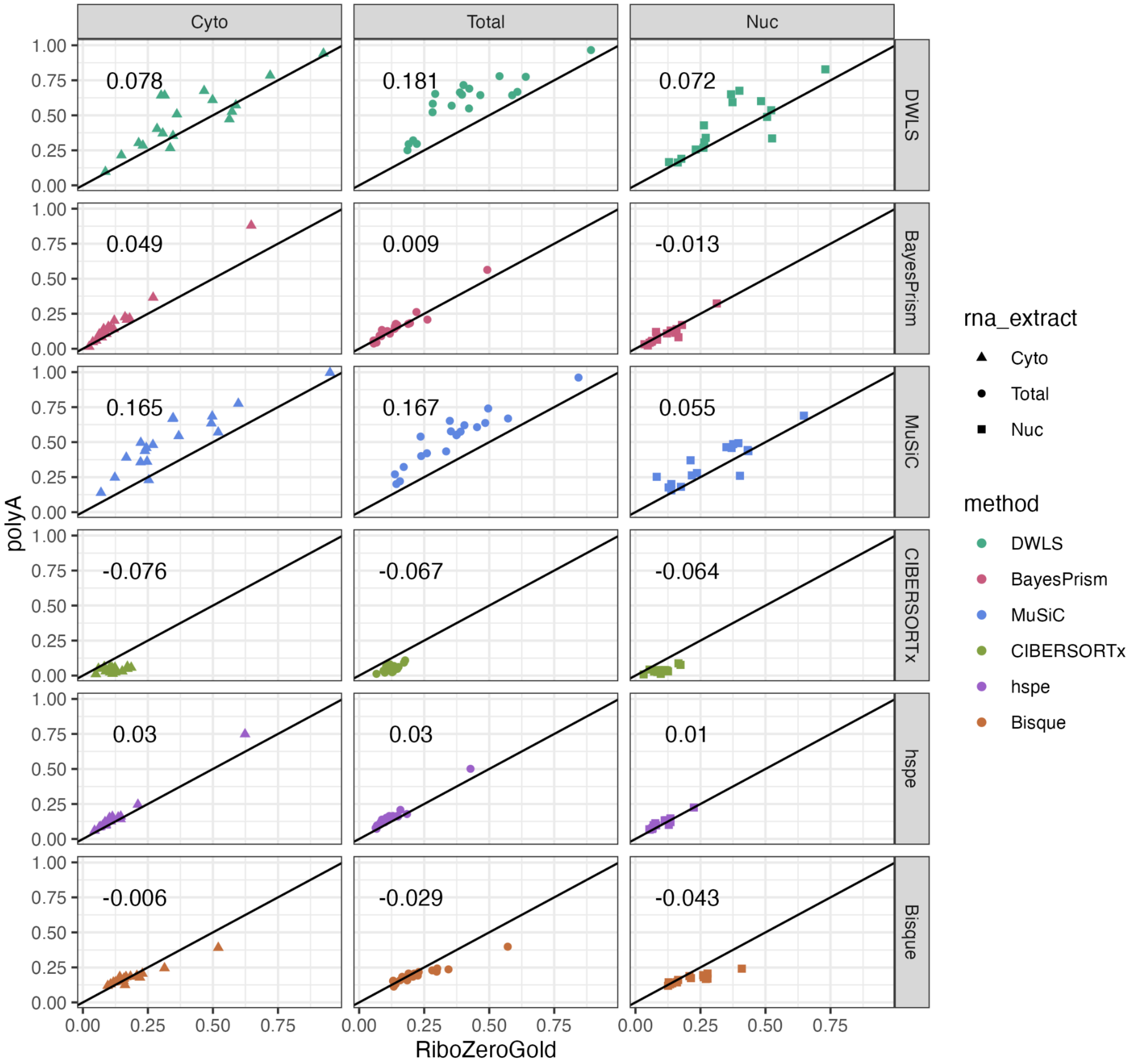

Fig S19: Oligodendrocyte estimated proportion consistency across polyA and RiboZeroGold. Estimated oligodendrocyte proportion by the evaluated deconvolution methods (rows) using the *Mean Ratio top25* cell type marker genes as input. Proportions are compared between polyA and RiboZeroGold by RNA extraction (columns and shape). The $y = x$ line is shown as a black solid line. The mean difference (PolyA- RiboZeroGold) of estimated proportion Oligo is annotated in each plot. Related to **Figure 4**, **Fig S12**, **Fig S14**.

bioRxiv preprint doi: https://doi.org/10.1101/2024.02.09.579665; this version posted April 7, 2024. The copyright holder for this preprint (which was not certified by peer review) is the author/funder, who has granted bioRxiv a license to display the preprint in perpetuity. It is made available under aCC-BY 4.0 International license.

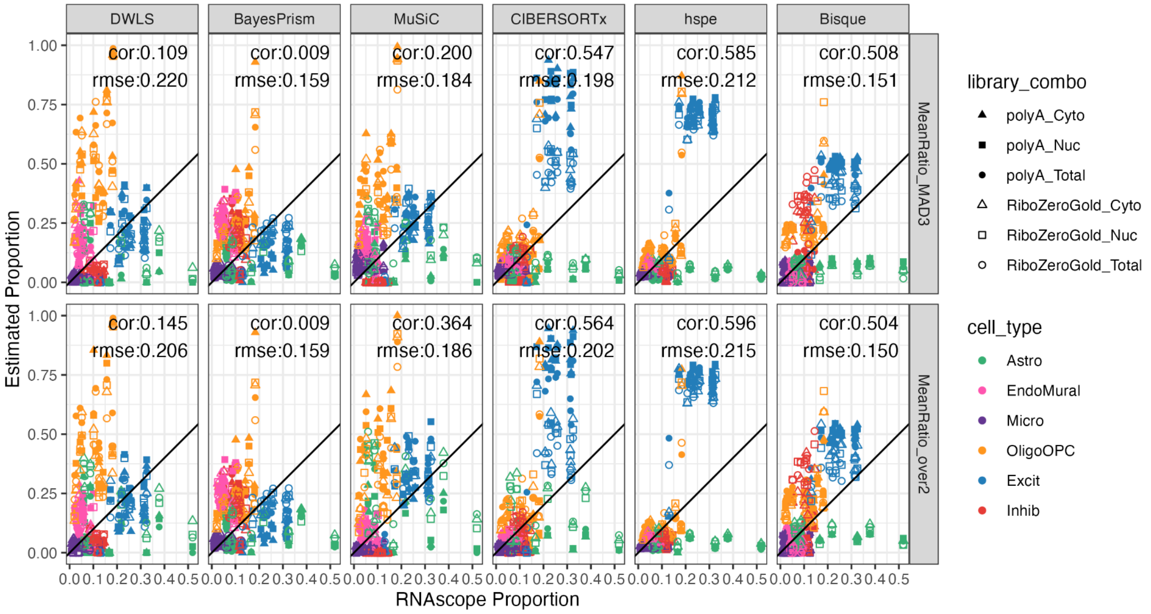

Fig S20: Cell composition results Mean Ratio over 2 and MAD3. Scatter plot of cell type proportions estimated by RNAScope/IF (x-axis) vs. the predicted cell type proportions by the deconvolution methods for Mean Ratio over 2 and Mean ratio MAD3 marker sets. Points are colored by cell type and shaped by the combination of the bulk RNA-seq sample's library type and RNA extraction. Pearson correlation (cor) and root mean squared error (rsme) values are shown for each panel. Related to **Figure 5**.

bioRxiv preprint doi: https://doi.org/10.1101/2024.02.09.579665; this version posted April 7, 2024. The copyright holder for this preprint (which was not certified by peer review) is the author/funder, who has granted bioRxiv a license to display the preprint in perpetuity. It is made available under aCC-BY 4.0 International license.

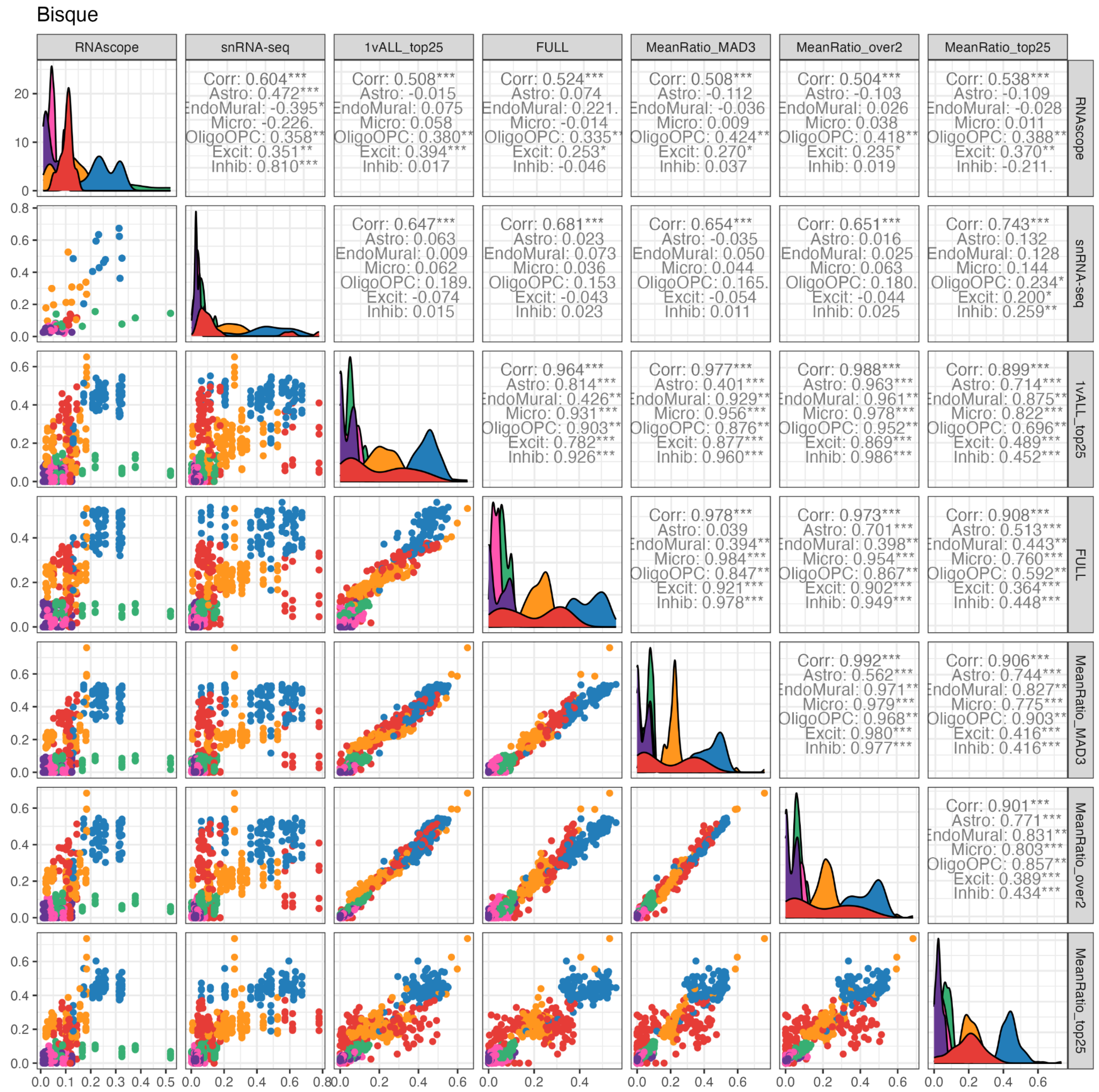

Fig S21: Cell composition comparison for *Bisque* results across marker gene selection methods. Pairwise scatter plots of measured and estimated cell type proportions from the RNAScope/IF experiments, snRNA-seq data, and *Bisque* across five marker gene sets. Cell type proportions are colored by cell type and shown in the lower triangle. Pearson correlation values (cor) calculated by `ggpairs()` from *GGally* [74] for each cell type are shown in the upper triangle. Density plots of the proportions are shown in the diagonal panels. Related to **Figure 5**.

bioRxiv preprint doi: https://doi.org/10.1101/2024.02.09.579665; this version posted April 7, 2024. The copyright holder for this preprint (which was not certified by peer review) is the author/funder, who has granted bioRxiv a license to display the preprint in perpetuity. It is made available under aCC-BY 4.0 International license.

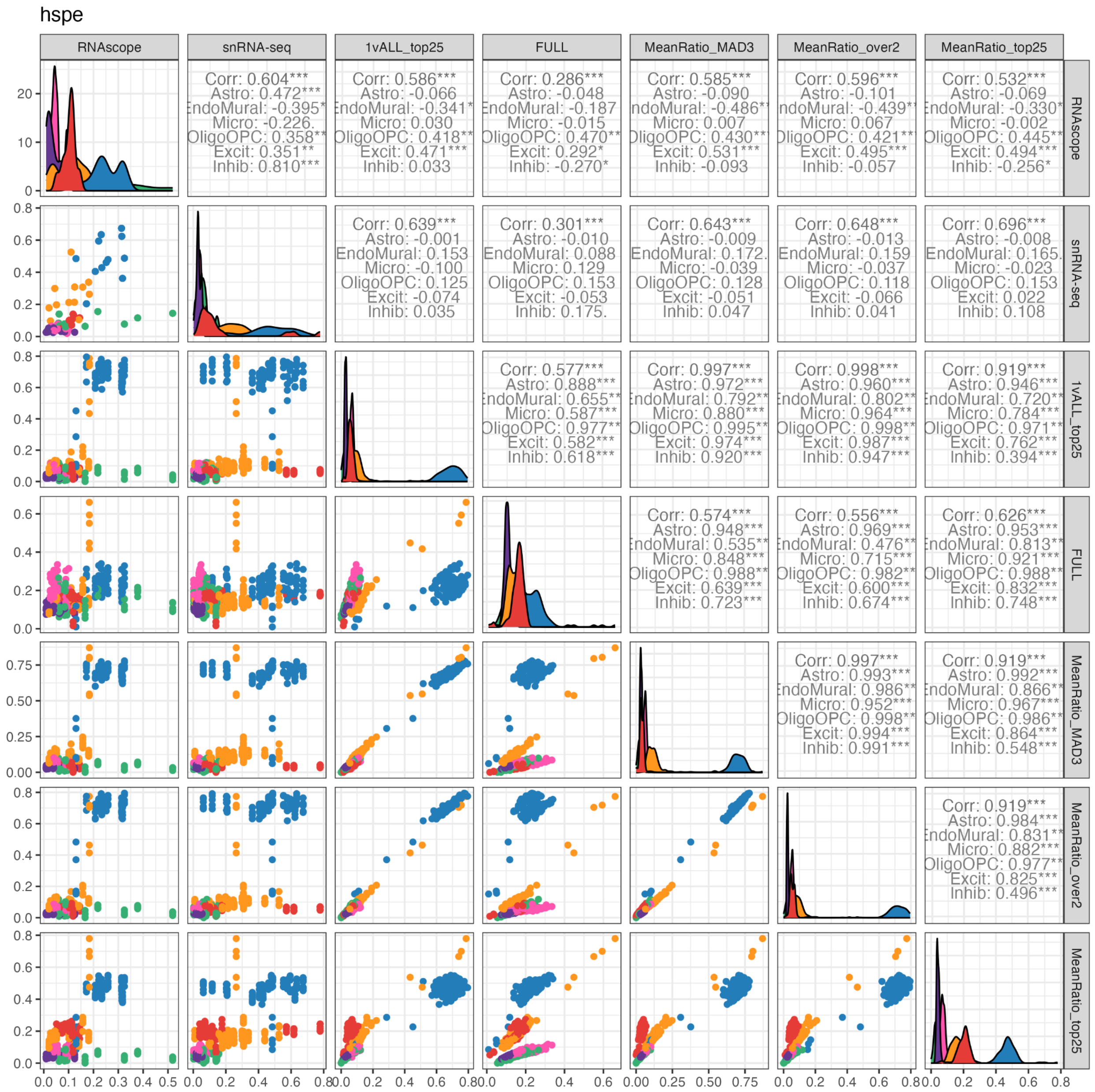

Fig S22: Cell composition comparison for *hspe* results across marker gene selection methods. Pairwise scatter plots of measured and estimated cell type proportions from the RNAScope/IF experiments, snRNA-seq data, and *hspe* across five marker gene sets**.** Cell type proportions are colored by cell type and shown in the lower triangle. Pearson correlation values (cor) calculated by `ggpairs()` from *GGally* [74] for each cell type are shown in the upper triangle. Density plots of the proportions are shown in the diagonal panels. Related to **Figure 5**.

bioRxiv preprint doi: https://doi.org/10.1101/2024.02.09.579665; this version posted April 7, 2024. The copyright holder for this preprint (which was not certified by peer review) is the author/funder, who has granted bioRxiv a license to display the preprint in perpetuity. It is made available under aCC-BY 4.0 International license.

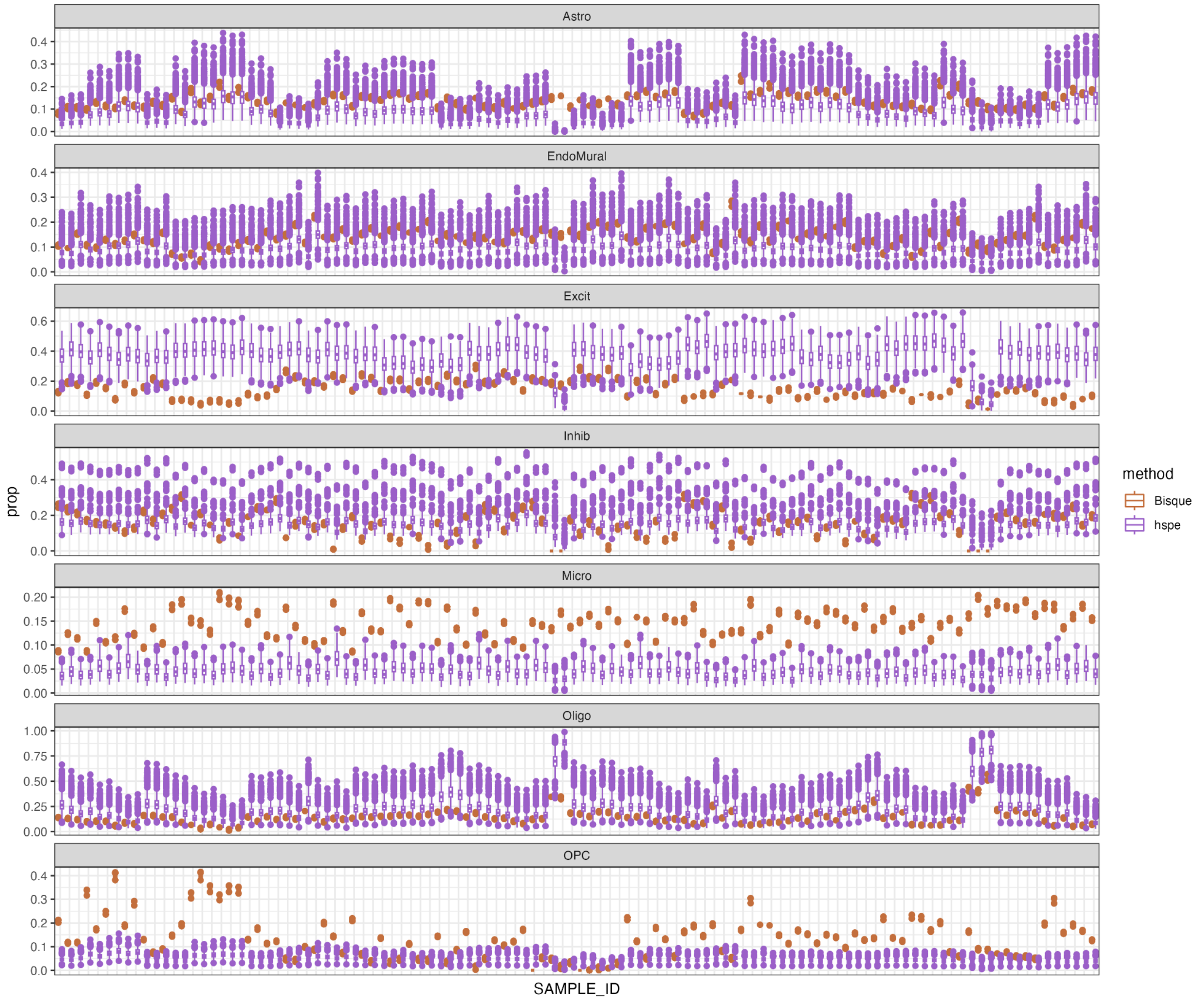

Fig S23: Simulation results from downsampling to equal input cell proportions for *Bisque* and *hspe*. Boxplots of estimated cell type proportions from simulated equal proportion subsets snRNA-seq reference data, repeated 1,000 times. Deconvolution results shown for all 110 bulk RNA-seq samples (X-axis).

bioRxiv preprint doi: https://doi.org/10.1101/2024.02.09.579665; this version posted April 7, 2024. The copyright holder for this preprint (which was not certified by peer review) is the author/funder, who has granted bioRxiv a license to display the preprint in perpetuity. It is made available under aCC-BY 4.0 International license.

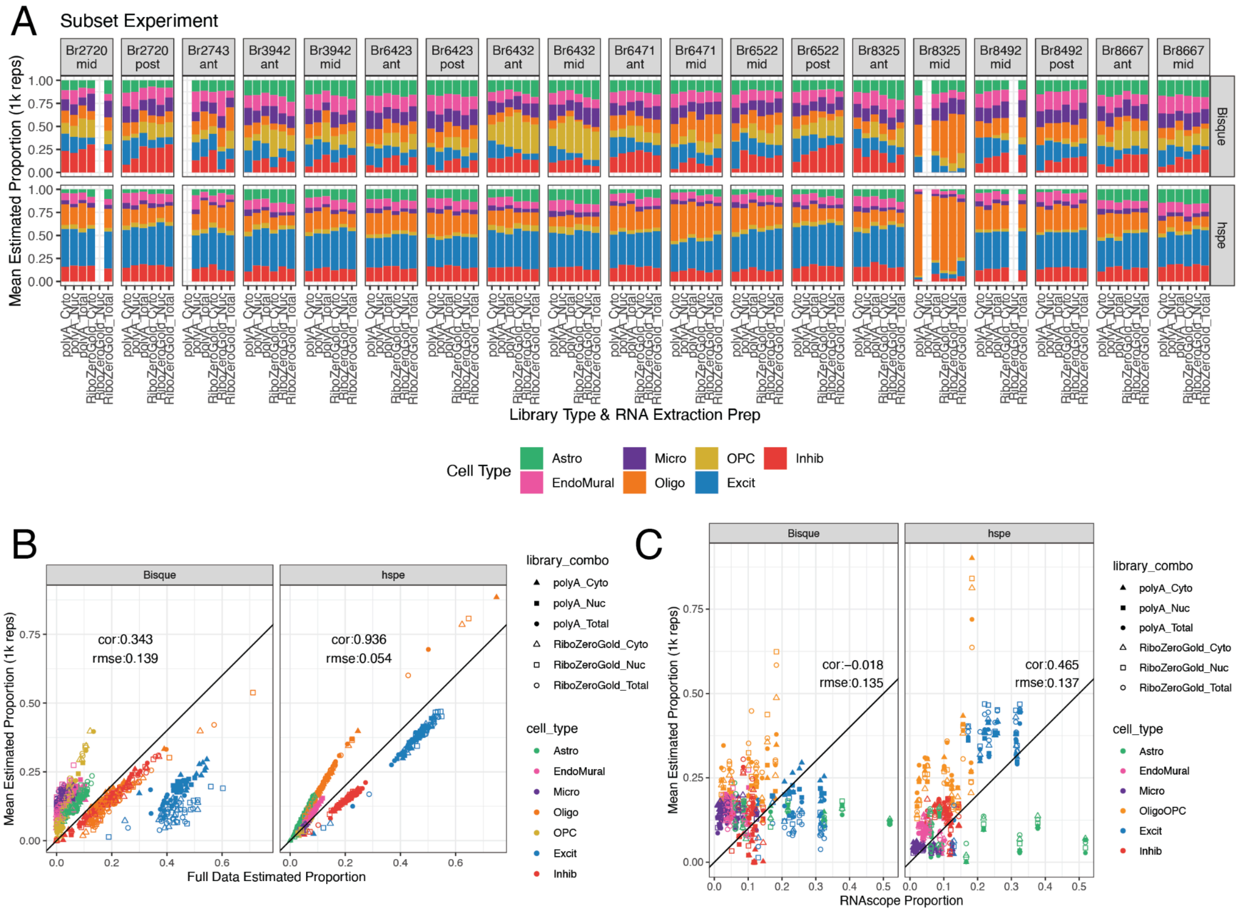

Fig S24: Performance of *Bisque* and *hspe* on downsampled equal proportion reference data. A. Composition bar plots displaying the mean estimated proportions from the 1,000 sampling replicates for each bulk RNA-seq sample for both methods tested. **B.** Scatter plot of cell type proportions estimated by *Bisque* and *hspe* with full input data vs. the mean predicted cell type proportions by the deconvolution methods under the 1,000 sampling replicates. Points are colored by the cell type and shaped by the combination of bulk RNA-seq RNA extraction method and library type. The annotation lists the overall Pearson's correlation (cor) and root mean squared error (rmse). **C.** Scatter plot of cell type proportions estimated by RNAScope/IF (X-axis) vs. the mean predicted cell type proportions by the deconvolution methods, similar to *B*. Related to **Fig S23**.

bioRxiv preprint doi: https://doi.org/10.1101/2024.02.09.579665; this version posted April 7, 2024. The copyright holder for this preprint (which was not certified by peer review) is the author/funder, who has granted bioRxiv a license to display the preprint in perpetuity. It is made available under aCC-BY 4.0 International license.

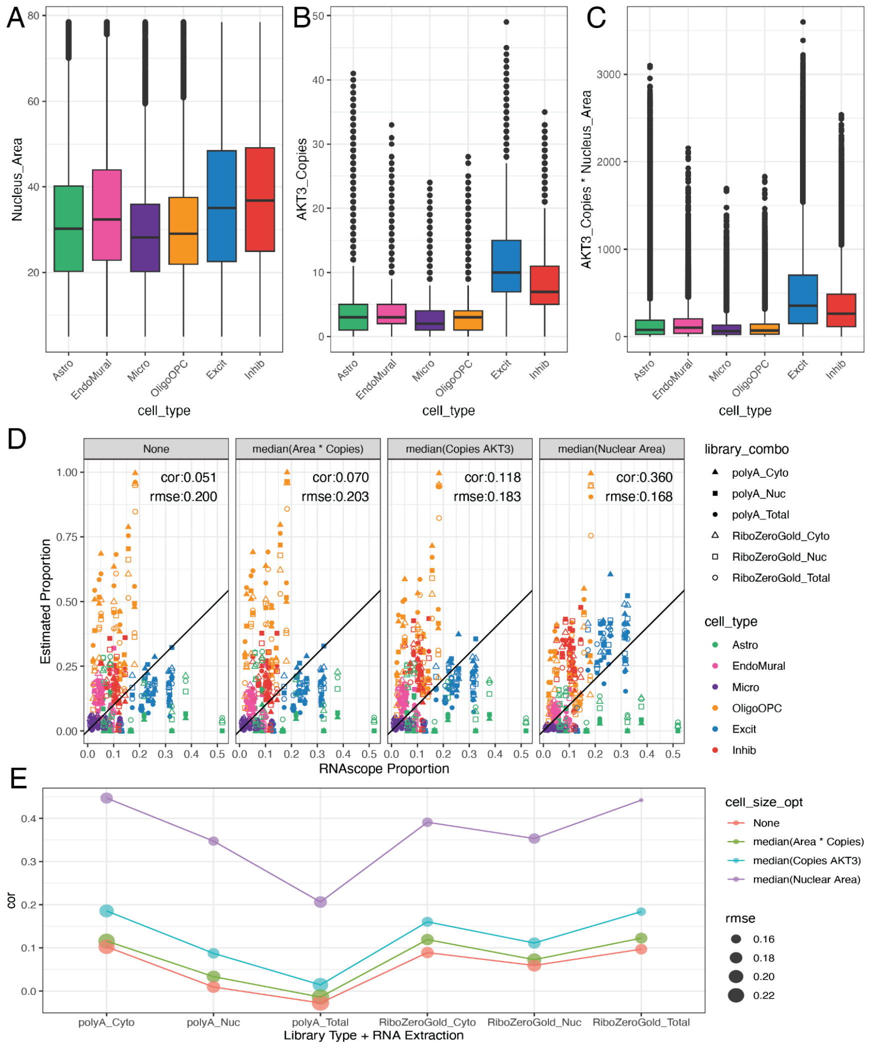

Fig S25: **Adjusting for cell size with *MuSiC***. Boxplot of the cell size metrics derived from the RNAScope/IF data **A.** nuclear area **B.** Copies of the total RNA expression gene *AKT3*, and **C.** the product of multiplying the nuclear area and number of *AKT3* copies. **D**. Scatter plot of cell type proportions estimated by RNAScope/IF (x-axis) vs. the predicted cell type proportions by *MuSiC* with various cell size metrics. Points are colored by the cell type and shaped by the combination of bulk RNA-seq RNA extraction method and library type. The annotation lists the overall Pearson’s correlation (cor) and root mean squared error (rmse). **E**. Correlation (cor) between the predicted proportions by *MuSiC* with cell size metrics and the estimated RNAScope/IF proportions across RNA extraction method and library type combinations, point size reflects the rmse value.

bioRxiv preprint doi: https://doi.org/10.1101/2024.02.09.579665; this version posted April 7, 2024. The copyright holder for this preprint (which was not certified by peer review) is the author/funder, who has granted bioRxiv a license to display the preprint in perpetuity. It is made available under aCC-BY 4.0 International license.

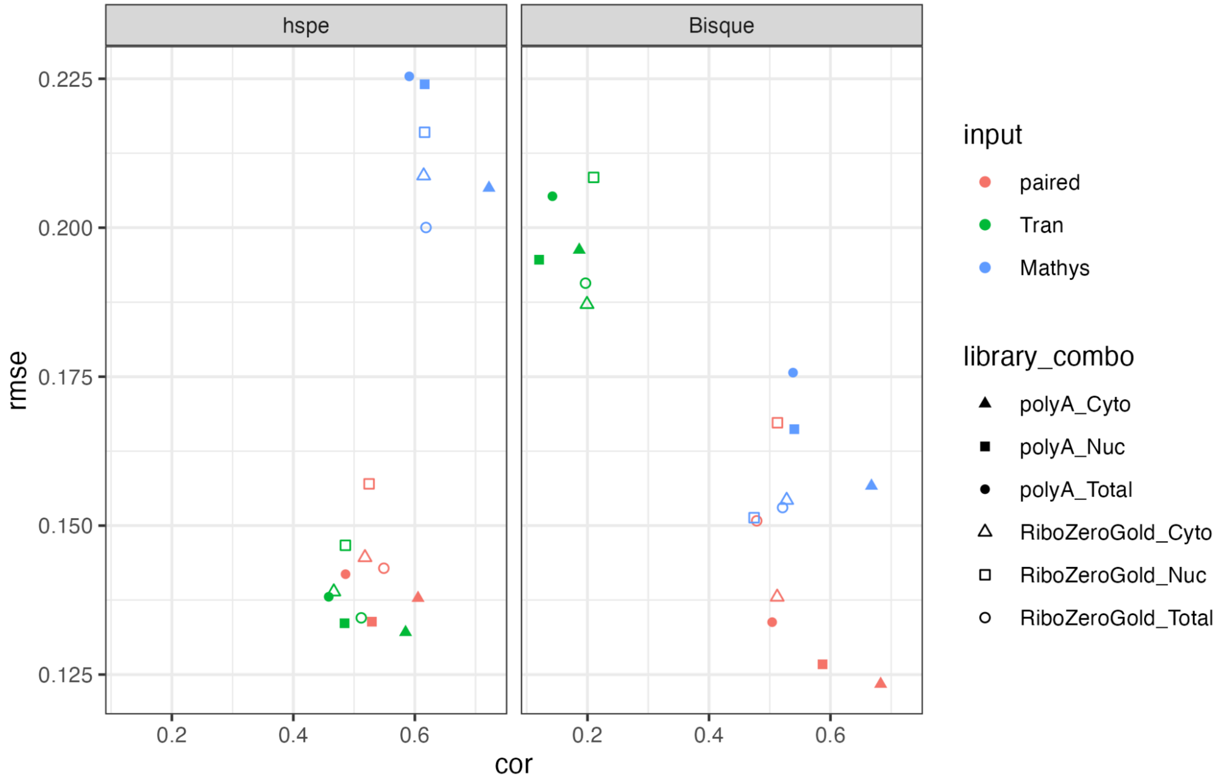

Fig S26: Scatter plot between the cor and rmse values for cell type proportion predictions across input datasets. Quality metrics for *hspe* and *Bisque* evaluated by bulk RNA-seq RNA extraction method and library type (shape), for the tree tested snRNA-seq input datasets (point color). Related to **Figure 6D-E**.

bioRxiv preprint doi: https://doi.org/10.1101/2024.02.09.579665; this version posted April 7, 2024. The copyright holder for this preprint (which was not certified by peer review) is the author/funder, who has granted bioRxiv a license to display the preprint in perpetuity. It is made available under aCC-BY 4.0 International license.

Supplemental Tables

Table S1: Donor demographics and bulk RNA-sequencing *SPEAQeasy* metrics. *SPEAQeasy* [61] metrics are documented at https://research.libd.org/SPEAQeasy/outputs.html#quality-metrics.

Table S2: Differential Gene Expression results between library types Output from *DREAM* and `limma::topTable` including the log fold change, average expression, *t*-statistic, *p*-value, adjusted *p*-value (FDR), B (log-odds), and *z*-statistic. Separated by RNA extraction. Related to **Figure 1E**.

Table S3: Differential Gene Expression results between RNA extractions. Similar to **Table S2.** Separated by library preparation. Related to **Fig S4**.

Table S4: **Differential Gene Expression results between bulk RNA-seq and snRNA-seq** Similar to **Table S2**. Separated by library preparation. Related to **Figure 1F**.

Table S5: RNAScope/IF Combination Summary. RNAScope/IF Circle and Star combinations of antibodies or probes used. Related to **Figure 2**.

RNAScope/IF Combination Summary							
Combination Circle				Combination Star			
Antibody/ Probe	Dilution Factor	Alexa/ Opal Dye	Dilution Factor	Antibody/ Probe	Dilution Factor	Alexa/ Opal Dye	Dilution Factor
GFAP	1:50	Alexa 594	1:400	TMEM119	1:9.5	Alexa 555	1:200
Claudin5 (CLDN5)	1:400	Alexa 488	1:400	OLIG2	1:19	Alexa 647	1:286
AKT3	None	Opal 570	1:1000	*AKT3*	None	Opal 620	1:1000
GAD1	1:50	Opal 690	1:1000	*SLC17A7*	1:50	Opal 520	1:1000

Table S6: RNAScope/IF and snRNA-seq cell type proportions for each sample. Image confidence, number of cells, and proportion for each cell type from RNAScope/IF, as well as cell count and proportion for snRNA-seq data (sn). Related to **Figure 2**.

Table S7: **Marker gene statistics from *Mean Ratio* & *1vALL* methods.** The table lists the

bioRxiv preprint doi: https://doi.org/10.1101/2024.02.09.579665; this version posted April 7, 2024. The copyright holder for this preprint (which was not certified by peer review) is the author/funder, who has granted bioRxiv a license to display the preprint in perpetuity. It is made available under aCC-BY 4.0 International license.

gene ENSEMBL ID, the target cell type and the mean expression of the target cell type, the highest non-target cell type and associated mean expression, *Mean Ratio*, and *Mean Ratio* rank. Statistics from *1vALL*, and membership in the four marker gene sets are also included. Related to **Figure 3**.

Table S8: **Estimated cell type proportions from the six deconvolution methods and five marker gene sets.** This table lists the details of the bulk RNA-seq sample, the deconvolution method and maker set, the estimated cell type proportion "prop", and the corresponding RNAScope/IF, or snRNA-seq cell type proportion. Related to **Figure 4** and **Figure 5**.

Table S9: Cell size arguments supplied to *MuSiC* from RNAScope/IF data. The median nuclear area, median copies of TREG *AKT3*, and the median of the product of the nuclear area and *AKT3* copies for each of the six cell types observed in RNAScope/IF. Related to **Fig S25A-C**.

Table S10: **Estimated cell type proportions from *MuSiC* adjusting for cell size.** This table lists the details of the bulk RNA-seq sample, and the cell size option used in *MuSiC* (from **Table S9**), the estimated cell type proportion "prop", and the corresponding RNAScope/IF, or snRNA-seq cell type proportion. Related to **Fig S25D-E**.

Table S11: Estimated cell type proportions from *hspe and Bisque* with other snRNA-seq input data. This table lists the details of the bulk RNA-seq sample, the deconvolution method and maker set, the input snRNA-seq reference dataset, the estimated cell type proportion "prop", and the corresponding RNAScope/IF, or snRNA-seq cell type proportion. Related to **Figure 6D-E**.
