# Supplementary material for: Benchmark of cellular deconvolution methods using a multi-assay reference dataset from postmortem human prefrontal cortex
Source: bioRxiv. 2024 Feb 12:2024.02.09.579665. Preprint. [Version 1] doi: 10.1101/2024.02.09.579665 (PMC10888823; doi:10.1101/2024.02.09.579665)
Supplement: Supplementary file 1 [file NIHPP2024.02.09.579665V1-supplement-1.pdf]

bioRxiv preprint doi: https://doi.org/10.1101/2024.02.09.579665; this version posted February 12, 2024. The copyright holder for this preprint (which was not certified by peer review) is the author/funder, who has granted bioRxiv a license to display the preprint in perpetuity. It is made available under aCC-BY 4.0 International license.

Supplemental Figures

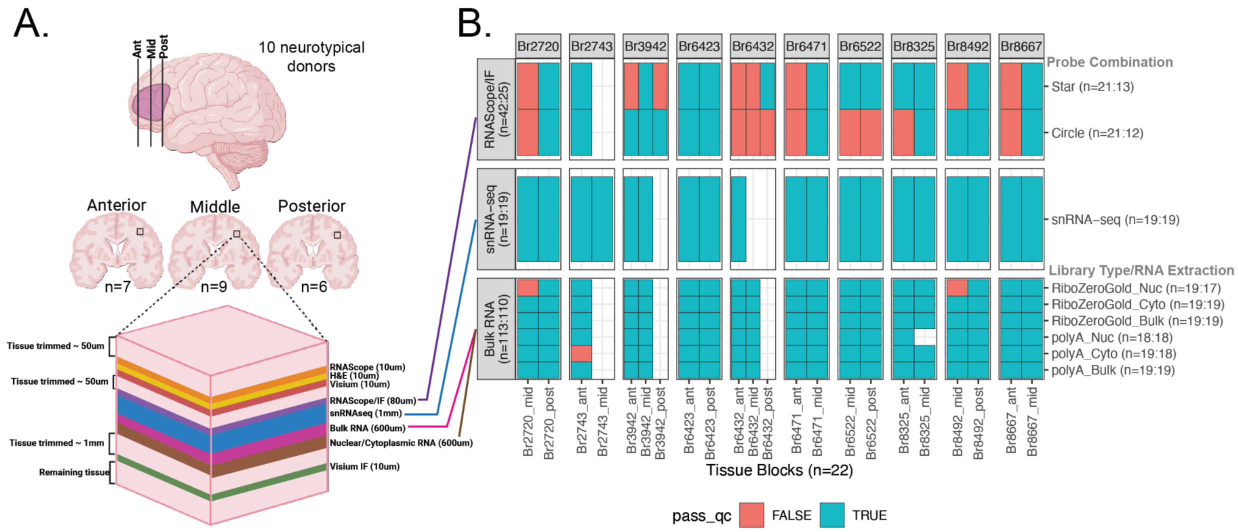

Fig S1: Schematic of assays performed on each tissue block. (**A**) Schematic of DLPFC dissections and DLPFC tissue block position depicting order of assays completed (n=10 donors; n=22 tissue blocks, including 7 Anterior, 9 Middle, 6 Posterior). Approximately 50 µm of tissue was trimmed to achieve a flat surface for cryosectioning. Next, several ~10 µm sections were collected for anatomical validation (RNAScope/IF, H&E) and Visium experiments [38]. Blocks were stored at -80℃ until completion of these assays. At the next cryostat session, blocks were trimmed and ~1 mm of tissue was collected for snRNA-seq (n=19) [38], ~600 µm of tissue was collected for total RNA extraction for bulk-RNAseq, and ~600 µm of tissue was collected for fractionated RNA extraction for nuclear and cytoplasmic RNA-seq. Finally, four tissue blocks were placed back on the cryostat and trimmed again to obtain a flat surface prior to collecting a ~10 µm Visium-spatial proteogenomics (SPG) tissue section [38]. (**B**) Tile plot illustrating which assays and configurations (probe/antibody combination for RNAScope/IF and library type/RNA extraction for RNA-seq) were performed on each tissue block and the sample size for that assay before and after quality control (qc) in the format "(n=before:after)". The tile is blank if an assay configuration was not performed on the tissue block. The tile is blue if the sample passed qc checks and was included in the analysis. Red tiles are not included in the study.

bioRxiv preprint doi: https://doi.org/10.1101/2024.02.09.579665; this version posted February 12, 2024. The copyright holder for this preprint (which was not certified by peer review) is the author/funder, who has granted bioRxiv a license to display the preprint in perpetuity. It is made available under aCC-BY 4.0 International license.

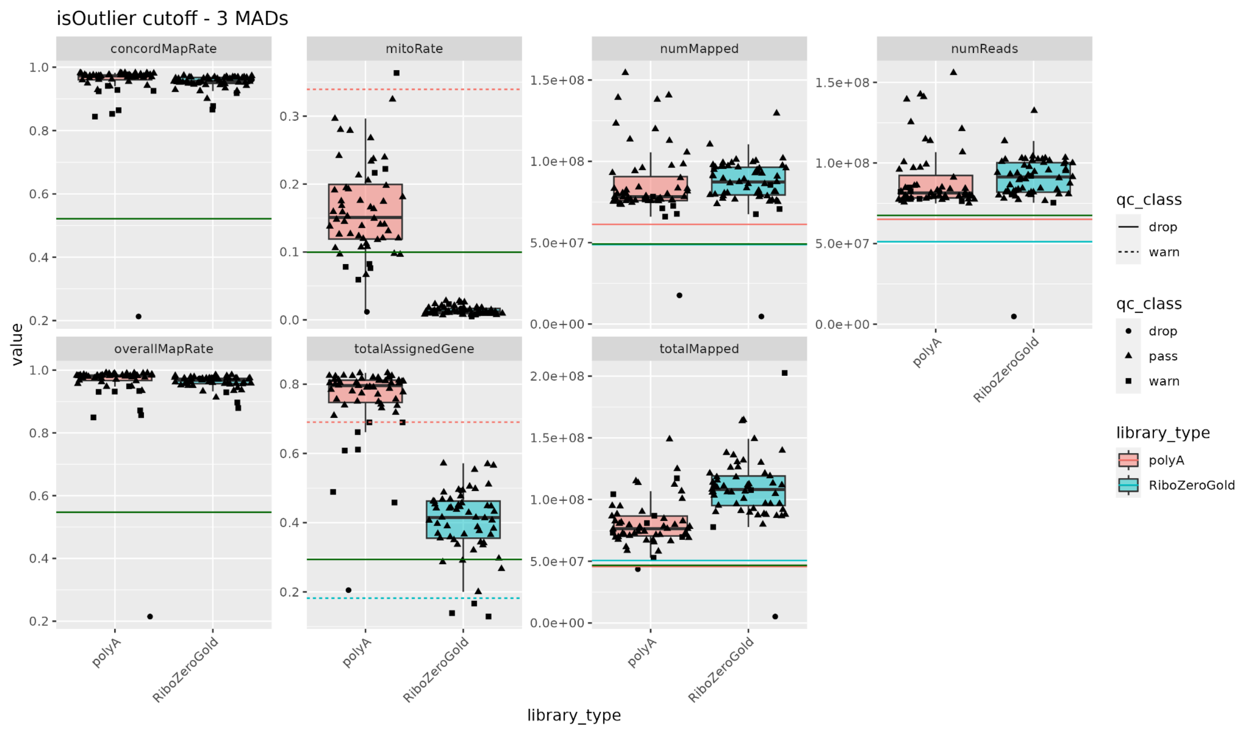

Fig S2: Bulk RNA-seq data Quality Control. Samples are evaluated for low `concordMapRate`, `numMapped`, `numReads`, `overallMapRate`, `totalAssignedGene`, and `totalMapped` or high `mitoRate`. See the *SPEAQeasy* [52] documentation at https://research.libd.org/SPEAQeasy/outputs.html#quality-metrics for the definition of these variables. Cutoffs (horizontal lines) were determined by a 3 median absolute deviations from the mean (3 MADs) from the distributions for the polyA or RiboZeroGold samples (line color) using isOutlier() from *scran* [39], as well as historic cutoffs from previous LIBD bulk RNA-seq projects (green lines). Based on the distribution of the values, and logic with the QC metrics some were “warning” cutoffs vs. “drop” cutoffs (line type). RNA-seq samples were classified as “drop”, “warn”, or “pass” based on their relationship to the cutoffs.

bioRxiv preprint doi: https://doi.org/10.1101/2024.02.09.579665; this version posted February 12, 2024. The copyright holder for this preprint (which was not certified by peer review) is the author/funder, who has granted bioRxiv a license to display the preprint in perpetuity. It is made available under aCC-BY 4.0 International license.

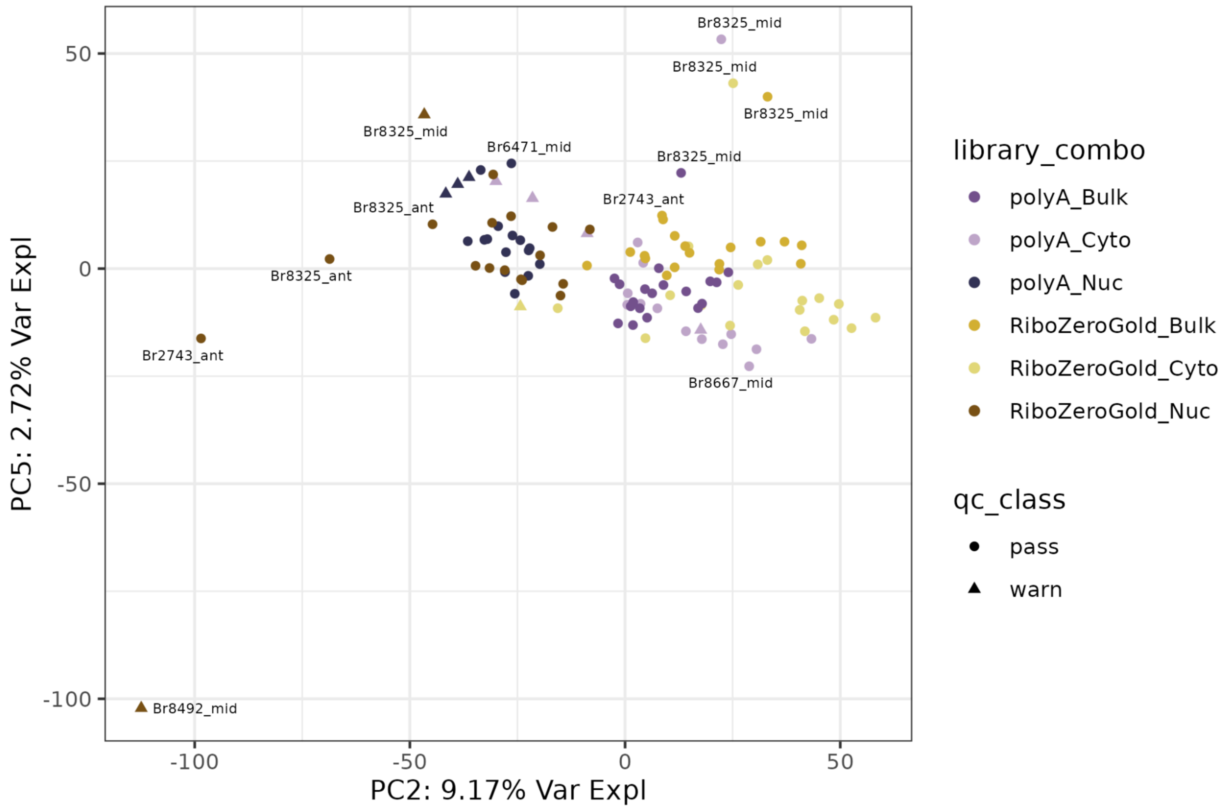

Fig S3: Bulk RNA-seq data Quality Control Principal Component Analysis (PCA). PC2 versus 5 colored by "library combo" (library type + RNA extraction). The sample `AN00000906_Br8492_Mid_Nuc` was identified as an outlier and removed from downstream analysis.

bioRxiv preprint doi: https://doi.org/10.1101/2024.02.09.579665; this version posted February 12, 2024. The copyright holder for this preprint (which was not certified by peer review) is the author/funder, who has granted bioRxiv a license to display the preprint in perpetuity. It is made available under aCC-BY 4.0 International license.

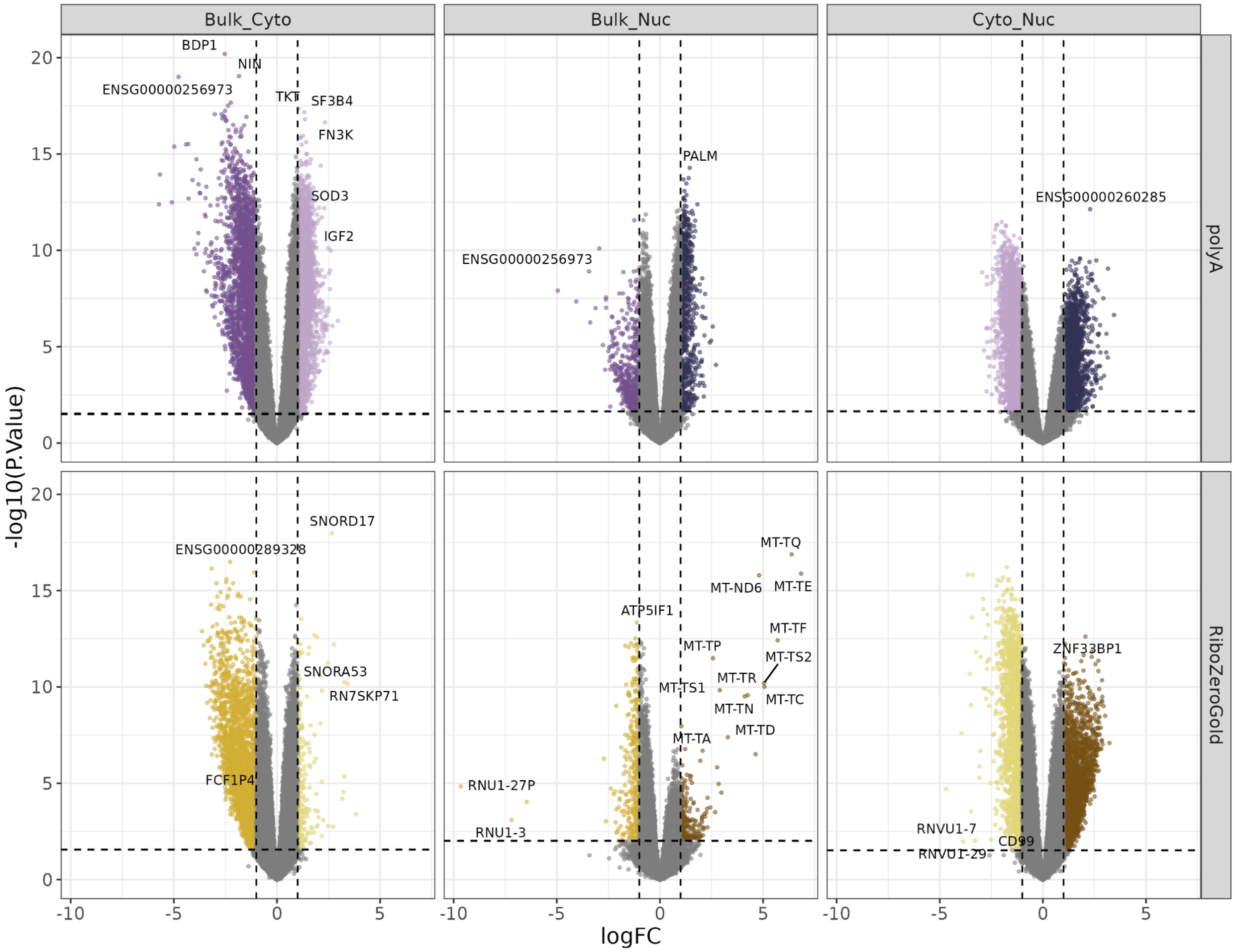

Fig S4: Volcano plots for RNA extraction Differential Gene Expression analysis. Samples were separated by library types (rows) and the RNA extractions: cytosolic (Cyto, light color), total cell (Bulk/Total, intermediate color), or nuclear (Nuc, dark color) samples were compared by differential expression in a pair-wise fashion (columns). Related to **Figure 1E.**

bioRxiv preprint doi: https://doi.org/10.1101/2024.02.09.579665; this version posted February 12, 2024. The copyright holder for this preprint (which was not certified by peer review) is the author/funder, who has granted bioRxiv a license to display the preprint in perpetuity. It is made available under aCC-BY 4.0 International license.

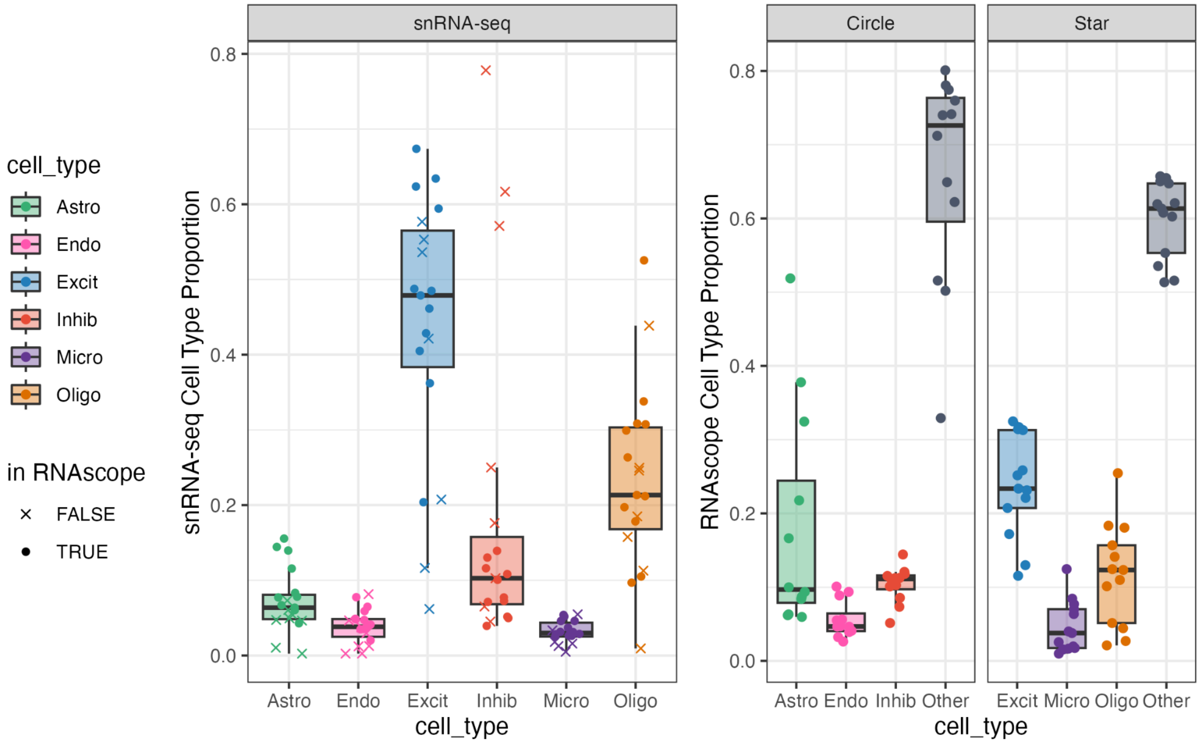

Fig S5: Boxplots of cell type proportions calculated from snRNA-seq and RNAScope/IF data. (**Left Side**) This side shows the snRNA-seq cell type proportions at the broad cell type resolution for all 19 tissue blocks for which snRNA-seq data was previously generated [38]. A filled dot marks tissue blocks for which RNAScope/IF data was also generated and passed quality control checks. Those absent are labeled with an x. (**Right Side**) This side shows the RNAScope/IF broad cell type proportions derived from the RNAScope/IF experiments for the Circle and Star combination of cell type markers. Cell type identities on the RNAScope/IF images were assigned using HALO (Indica Labs).

bioRxiv preprint doi: https://doi.org/10.1101/2024.02.09.579665; this version posted February 12, 2024. The copyright holder for this preprint (which was not certified by peer review) is the author/funder, who has granted bioRxiv a license to display the preprint in perpetuity. It is made available under aCC-BY 4.0 International license.

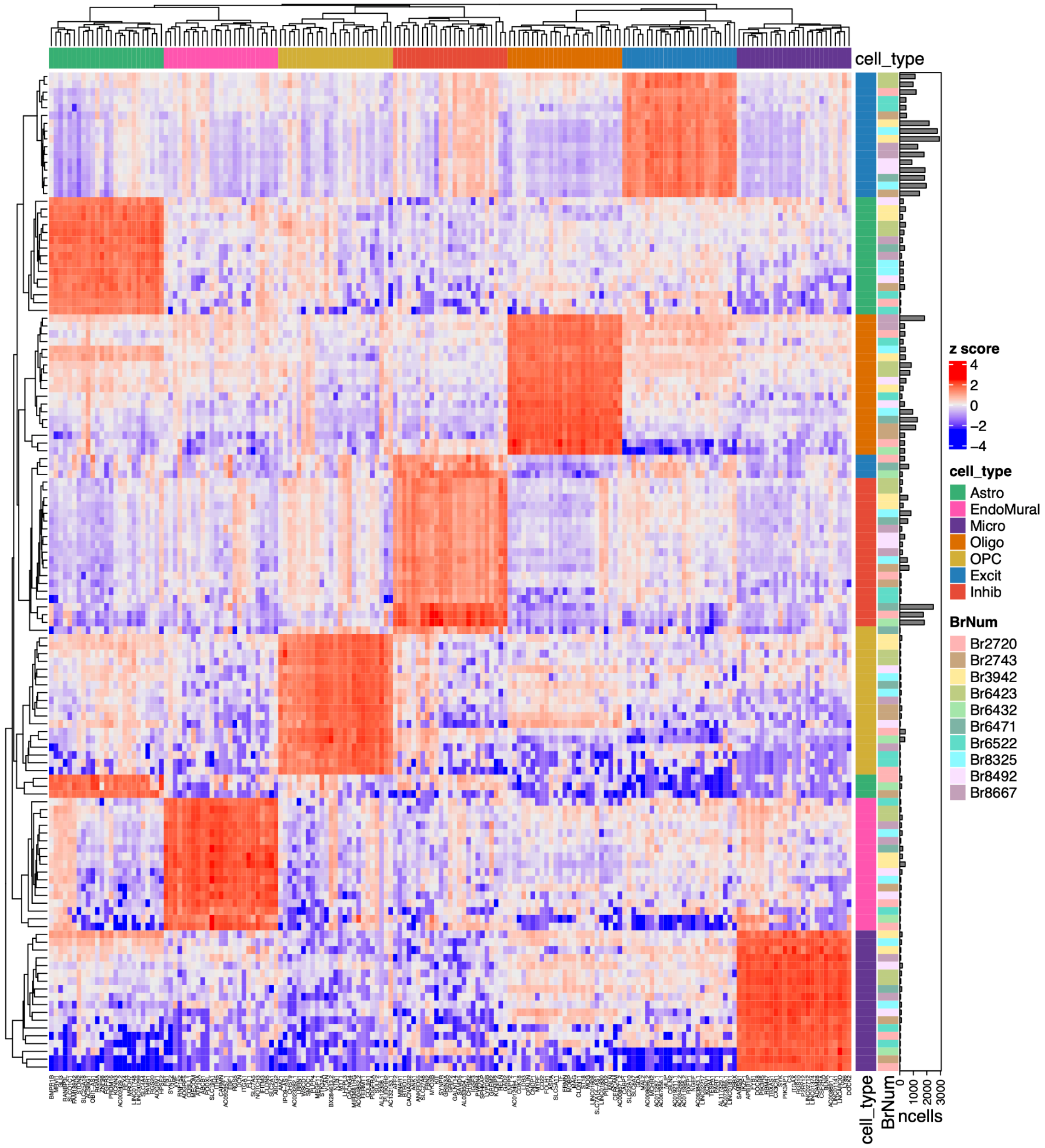

Fig S6: Heatmap of the top 25 *Mean Ratio* marker genes for deconvolution. Normalized snRNA-seq counts (logcounts) were centered and scaled by gene to compute Z scores. Brain donor identifiers (`BrNum`) and cell types were used to annotate this heatmap made with *ComplexHeatmap* [61]. Genes are shown in the columns and nuclei on the rows, with the total number of nuclei (ncells) visualized as side barplots.

bioRxiv preprint doi: https://doi.org/10.1101/2024.02.09.579665; this version posted February 12, 2024. The copyright holder for this preprint (which was not certified by peer review) is the author/funder, who has granted bioRxiv a license to display the preprint in perpetuity. It is made available under aCC-BY 4.0 International license.

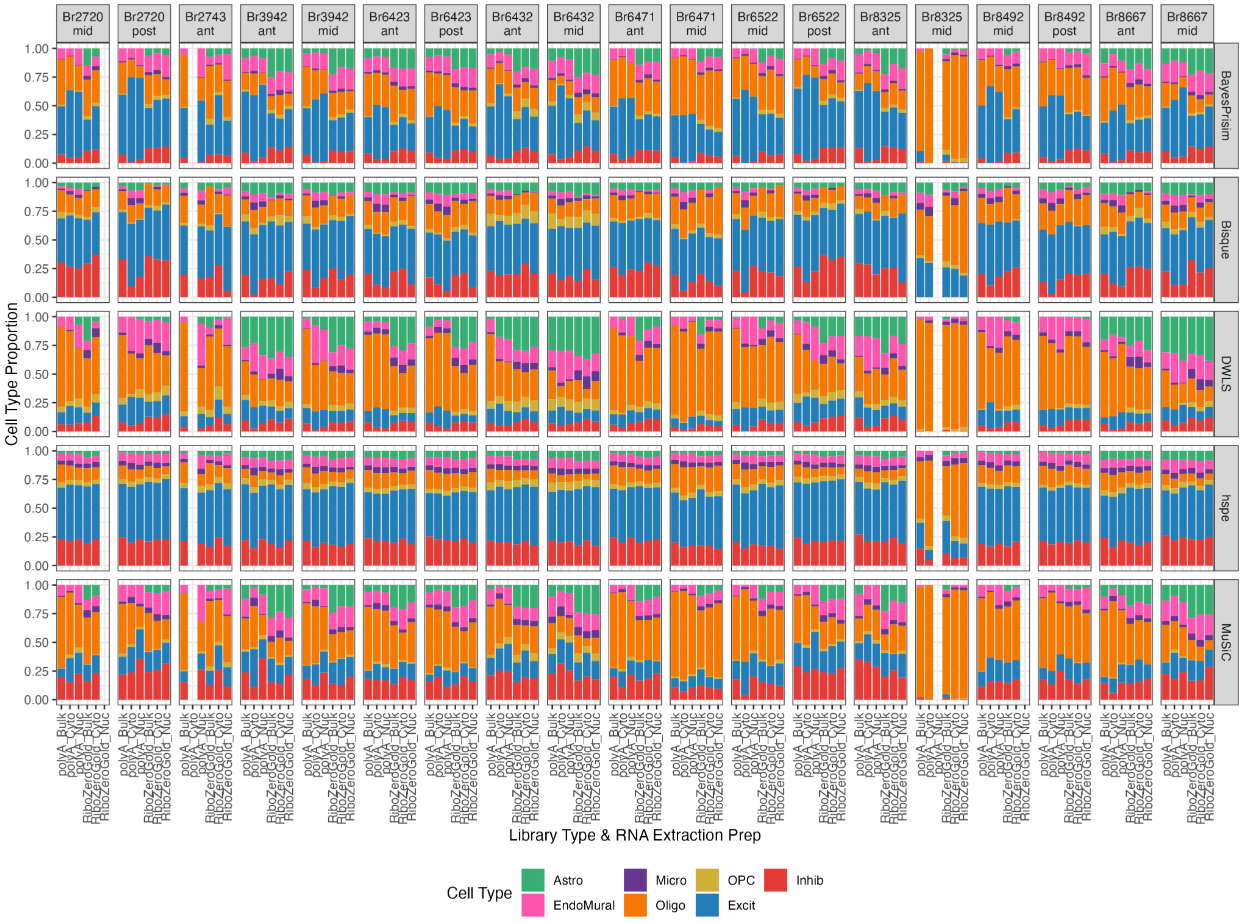

Fig S7: Barplots of estimated cell type proportions from deconvolution methods. Each tissue block is a column, the x-axis categories are the six RNA extraction and library type combinations for the 110 bulk RNA-seq samples. The rows are the predictions from each of the five deconvolution methods. Columns are labeled by the tissue block, which is a combination of the brain donor identifier (`BrNum`) and the anterior-posterior axis location of the tissue block (anterior: ant, middle: mid, or posterior: post).

bioRxiv preprint doi: https://doi.org/10.1101/2024.02.09.579665; this version posted February 12, 2024. The copyright holder for this preprint (which was not certified by peer review) is the author/funder, who has granted bioRxiv a license to display the preprint in perpetuity. It is made available under aCC-BY 4.0 International license.

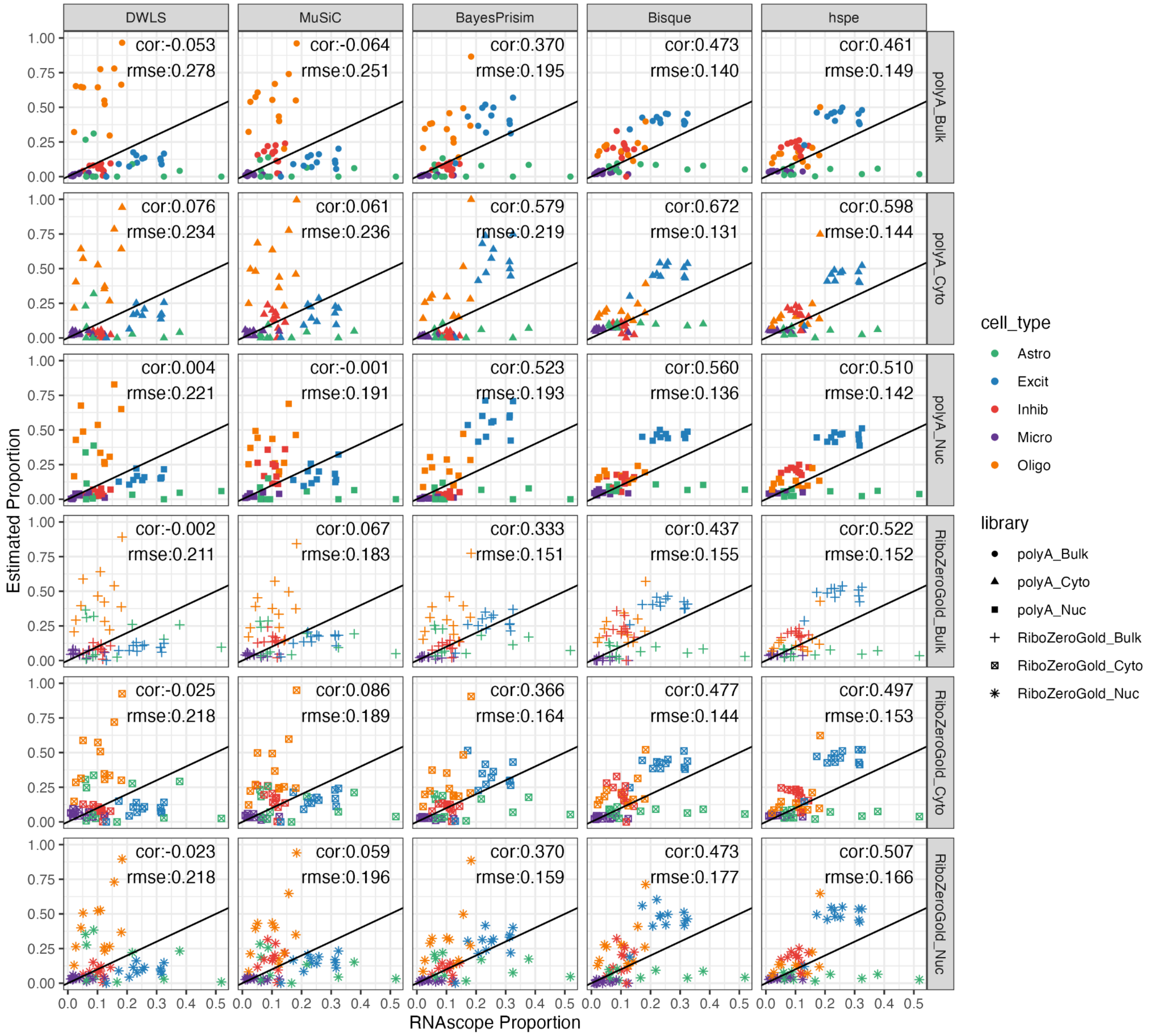

Fig S8: Scatter plots of cell type proportions measured by RNAScope/IF experiment vs. estimated proportions from deconvolution methods. Points are colored by cell type and shaped by the combination of the bulk RNA-seq sample's library type and RNA extraction. Pearson correlation (cor) and root mean squared error (rsme) values are shown for each panel. Related to **Figure 4.**

bioRxiv preprint doi: https://doi.org/10.1101/2024.02.09.579665; this version posted February 12, 2024. The copyright holder for this preprint (which was not certified by peer review) is the author/funder, who has granted bioRxiv a license to display the preprint in perpetuity. It is made available under aCC-BY 4.0 International license.

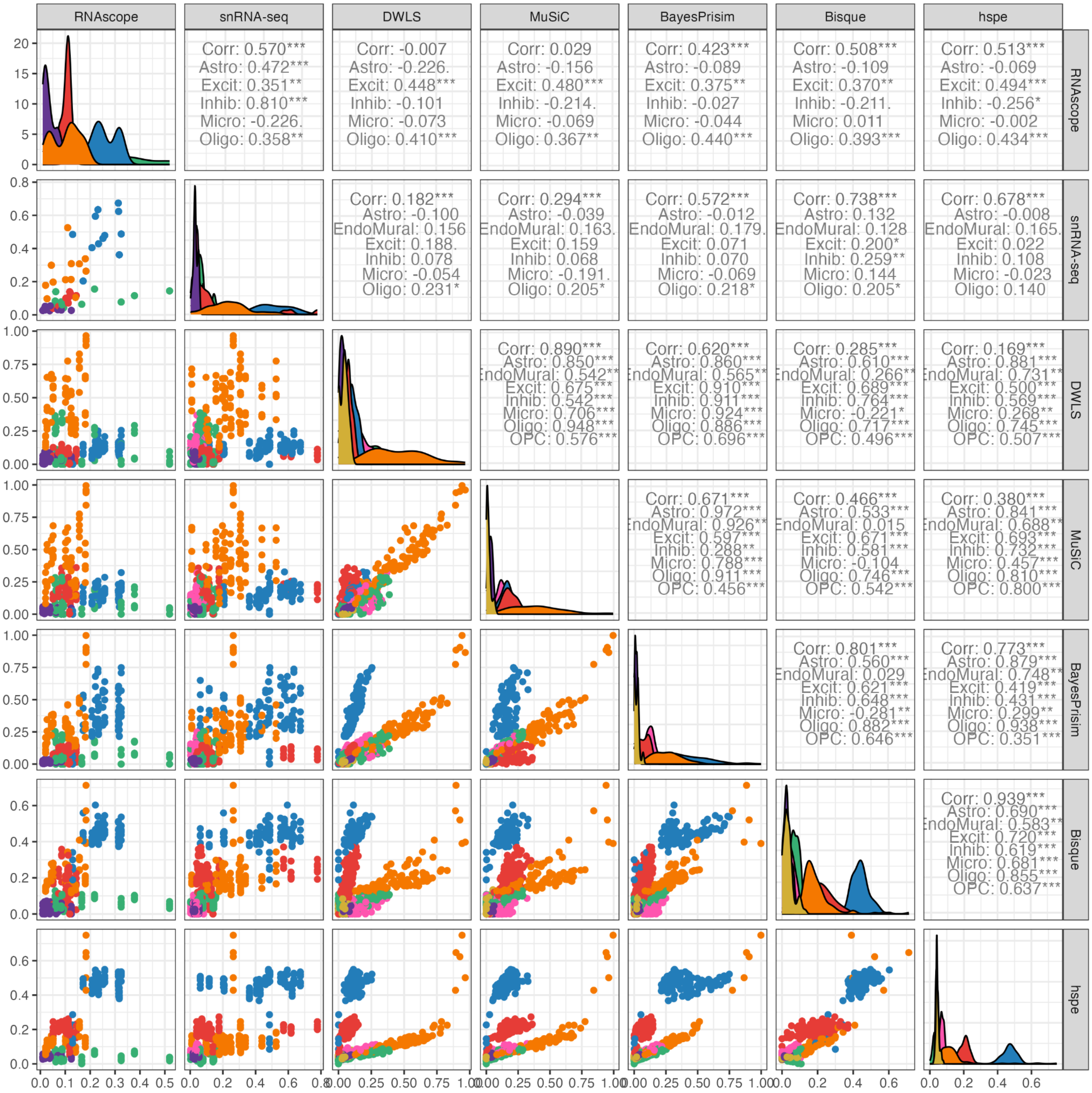

Fig S9: Pairwise scatter plots of measured and estimated cell type proportions from the RNAScope/IF experiments, snRNA-seq data, and deconvolution methods. Cell type proportions are colored by cell type are shown in the lower triangle. Pearson correlation values (cor) calculated by `ggpairs()` from *GGally* [63] for each cell type are shown in the upper triangle. Density plots of the proportions are shown in the diagonal panels.

bioRxiv preprint doi: https://doi.org/10.1101/2024.02.09.579665; this version posted February 12, 2024. The copyright holder for this preprint (which was not certified by peer review) is the author/funder, who has granted bioRxiv a license to display the preprint in perpetuity. It is made available under aCC-BY 4.0 International license.

Supplemental Tables

Table S1: Donor demographics and bulk RNA-sequencing *SPEAQeasy* metrics. *SPEAQeasy* [52] metrics are documented at https://research.libd.org/SPEAQeasy/outputs.html#quality-metrics.

Table S2: RNAScope/IF Combination Summary. RNAScope/IF Circle and Star combinations of antibodies or probes used.

RNAScope/IF Combination Summary							
Combination Circle				Combination Star			
Antibody/ Probe	Dilution Factor	Alexa/ Opal Dye	Dilution Factor	Antibody/ Probe	Dilution Factor	Alexa/ Opal Dye	Dilution Factor
GFAP	1:50	Alexa 594	1:400	TMEM119	1:9.5	Alexa 555	1:200
Claudin5 (CLDN5)	1:400	Alexa 488	1:400	OLIG2	1:19	Alexa 647	1:286
AKT3	None	Opal 570	1:1000	*AKT3*	None	Opal 620	1:1000
GAD1	1:50	Opal 690	1:1000	*SLC17A7*	1:50	Opal 520	1:1000

Table S3: RNAScope/IF and snRNA-seq cell type proportions for each sample. Image confidence, number of cells, and proportion for each cell type from RNAScope, as well as cell count and proportion for snRNA-seq data (sn).

Table S4: **Marker gene statistics from *Mean Ratio* & *1vALL* methods.** The top 25 genes ranked by *Mean Ratio* for each cell type. The table lists the gene ENSEMBL ID, the target cell type and the mean expression of the target cell type, the highest non-target cell type and associated mean expression, *Mean Ratio*, and *Mean Ratio* rank. Statistics from 1vALL are also included.
